# Inflammatory and Coagulation Biomarkers and Mortality in Patients with HIV Infection

**DOI:** 10.1371/journal.pmed.0050203

**Published:** 2008-10-21

**Authors:** Lewis H Kuller, Russell Tracy, Waldo Belloso, Stephane De Wit, Fraser Drummond, H. Clifford Lane, Bruno Ledergerber, Jens Lundgren, Jacqueline Neuhaus, Daniel Nixon, Nicholas I Paton, James D Neaton, for the INSIGHT SMART Study Group

**Affiliations:** 1 University of Pittsburgh, Pittsburgh, Pennsylvania, United States of America; 2 University of Vermont, Burlington, Vermont, United States of America; 3 Hospital Italiano de Buenos Aires, Buenos Aires, Argentina; 4 Saint-Pierre Hospital, Brussels, Belgium; 5 National Centre in HIV Epidemiology and Clinical Research, University of New South Wales, Sydney, Australia; 6 National Institute of Allergy and Infectious Diseases, National Institutes of Health, Bethesda, Maryland, United States of America; 7 University Hospital, Zurich, Switzerland; 8 University of Copenhagen, Copenhagen, Denmark; 9 University of Minnesota, Minneapolis, Minnesota, United States of America; 10 Virginia Commonwealth University, Richmond, Virginia, United States of America; 11 Medical Research Council Clinical Trials Unit, London, United Kingdom; San Francisco General Hospital, United States of America

## Abstract

**Background:**

In the Strategies for Management of Anti-Retroviral Therapy trial, all-cause mortality was higher for participants randomized to intermittent, CD4-guided antiretroviral treatment (ART) (drug conservation [DC]) than continuous ART (viral suppression [VS]).

We hypothesized that increased HIV-RNA levels following ART interruption induced activation of tissue factor pathways, thrombosis, and fibrinolysis.

**Methods and Findings:**

Stored samples were used to measure six biomarkers: high sensitivity C-reactive protein (hsCRP), interleukin-6 (IL-6), amyloid A, amyloid P, D-dimer, and prothrombin fragment 1+2. Two studies were conducted: (1) a nested case–control study for studying biomarker associations with mortality, and (2) a study to compare DC and VS participants for biomarker changes. For (1), markers were determined at study entry and before death (latest level) for 85 deaths and for two controls (*n* = 170) matched on country, age, sex, and date of randomization. Odds ratios (ORs) were estimated with logistic regression. For each biomarker, each of the three upper quartiles was compared to the lowest quartile. For (2), the biomarkers were assessed for 249 DC and 250 VS participants at study entry and 1 mo following randomization. Higher levels of hsCRP, IL-6, and D-dimer at study entry were significantly associated with an increased risk of all-cause mortality. Unadjusted ORs (highest versus lowest quartile) were 2.0 (95% confidence interval [CI], 1.0–4.1; *p* = 0.05), 8.3 (95% CI, 3.3–20.8; *p* < 0.0001), and 12.4 (95% CI, 4.2–37.0; *p* < 0.0001), respectively. Associations were significant after adjustment, when the DC and VS groups were analyzed separately, and when latest levels were assessed. IL-6 and D-dimer increased at 1 mo by 30% and 16% in the DC group and by 0% and 5% in the VS group (*p* < 0.0001 for treatment difference for both biomarkers); increases in the DC group were related to HIV-RNA levels at 1 mo (*p* < 0.0001). In an expanded case–control analysis (four controls per case), the OR (DC/VS) for mortality was reduced from 1.8 (95% CI, 1.1–3.1; *p* = 0.02) to 1.5 (95% CI, 0.8–2.8) and 1.4 (95% CI, 0.8–2.5) after adjustment for latest levels of IL-6 and D-dimer, respectively.

**Conclusions:**

IL-6 and D-dimer were strongly related to all-cause mortality. Interrupting ART may further increase the risk of death by raising IL-6 and D-dimer levels. Therapies that reduce the inflammatory response to HIV and decrease IL-6 and D-dimer levels may warrant investigation.

**Trial Registration**: ClinicalTrials.gov (NCT00027352).

## Abstract 2

Analyzing biomarker data from participants in a previous randomized controlled trial of continuous versus interrupted HIV treatment (the SMART trial), James Neaton and colleagues find that mortality was related to IL-6 and fibrin D-dimers.

## Editors' Summary

**Background.:**

Globally, more than 30 million people are infected with the human immunodeficiency virus (HIV), the virus that causes acquired immunodeficiency syndrome (AIDS). HIV infects and destroys immune system cells (including CD4 cells, a type of lymphocyte). The first stage of HIV infection can involve a short flu-like illness but in the second stage, which can last many years, HIV replicates in the lymph glands (small immune system organs throughout the body) without causing any symptoms. Eventually, however, the immune system becomes so damaged that HIV-infected individuals begin to succumb to “opportunistic” infections (for example, bacterial pneumonia) and cancers (in particular, Karposi sarcoma) that the immune system would normally prevent. AIDS itself is characterized by one or more severe opportunistic infections or cancers (so-called AIDS-related diseases) and by a low blood CD4 cell count. HIV infections cannot be cured but antiretroviral therapy (ART)—combinations of powerful antiretroviral drugs—can keep them in check, so many HIV-positive people now have substantially improved life expectancy.

**Why Was This Study Done?:**

Unfortunately, the effectiveness of ART sometimes wanes over time and prolonged ART can cause unpleasant side effects. Consequently, alternative ART regimens are continually being tested in clinical trials. In the Strategies for Management of Anti-Retroviral Therapy (SMART) trial, for example, HIV-positive patients received either continuous ART (the viral suppression or VS arm), or ART only when their CD4 cell counts were below 250 cells/mm^3^ (the drug conservation or DC arm; the normal adult CD4 cell count is about 1,000 cells/mm^3^). Unexpectedly, more people died in the DC arm than in the VS arm from non-AIDS diseases (including heart and circulation problems), a result that led to the trial being stopped early. One possible explanation for these excess deaths is that increased HIV levels following ART interruption might have induced an inflammatory response (a non-specific immune response that occurs with infection or wounding) and/or a hypercoagulable state (a condition in which blood clots form inside undamaged blood vessels) and that these changes increased the risk of death from non-AIDS diseases. In this study, the researchers test this hypothesis.

**What Did the Researchers Do and Find?:**

The researchers measured the levels of proteins that indicate the presence of inflammation or increased coagulation (biomarkers) in stored blood samples from the 85 people who died during the SMART trial (55 and 30 of the participants assigned to receive DC and VS, respectively) and from 170 survivors who served as comparison (control) participants. (Two control participants were “matched” to each participant who had died (cases). In this “case-control” study, an increased risk of death was associated with higher levels at study entry of the inflammation biomarkers high-sensitivity C-reactive protein (hsCRP) and interleukin 6 (IL-6) and of the coagulation biomarker D-dimer. The risk of death among people with hsCRP values in the highest quarter of measured values was twice that among people with hsCRP values in the lowest quarter (this is expressed as an odds ratio of 2). For IL-6 and D-dimer, the equivalent odds ratios were 8.3 and 12.4, respectively. Furthermore, increases in hsCRP, IL-6 and D-dimer after study entry were associated with an increased risk of death. The researchers also measured blood levels of the same biomarkers in 250 randomly chosen patients from each of the two treatment arms. IL-6 levels increased by 30% over the first month of the trial in the DC arm but were unchanged in the VS arm. Over the same period, D-dimer levels increased by 16% and 5% in the DC and VS arms, respectively. Increases in both markers in the DC arm were related to HIV RNA levels after one month.

**What Do These Findings Mean?:**

Taken together, these findings suggest that HIV-induced activation of inflammation and coagulation increases the risk of death among HIV-positive patients and that interrupting ART further increases this risk, possibly by increasing IL-6 and D-dimer levels. Because only a small number of people died in this study, the relationship between these biomarkers and death and illness among treated and untreated HIV-positive individuals needs to be confirmed in further studies. However, these findings suggest that the development of therapies that reduce the effect that HIV replication has on inflammation and blood coagulation, or that reduce IL-6 and D-dimer levels, might extend the life-expectancy of HIV-positive people.

**Additional Information.:**

Please access these Web sites via the online version of this summary at http://dx.doi.org/10.1371/journal.pmed.0050203.

- Information is available from the US National Institute of Allergy and Infectious Diseases on HIV infection and AIDS and about the SMART trial
- HIV InSite has comprehensive information on all aspects of HIV/AIDS
- Information is also available from Avert, an international AIDS charity, on HIV/AIDS
- More information about the SMART trial is available on ClinicalTrials.gov, a database of clinical trials maintained by the US National Institutes of Health

## Introduction

The Strategies for Management of Anti-Retroviral Therapy (SMART) trial compared episodic use of antiretroviral treatment (ART) guided by CD4+ count with the current practice of continuous ART. Risk of opportunistic disease (OD) or death was more than twice as great for those in the episodic compared to the continuous ART group (hazard ratio = 2.6; *p* < 0.001). The episodic ART strategy was also associated with an 84% (*p* = 0.007) increased risk of all-cause mortality. Most of the deaths that occurred were not attributable to AIDS-defining conditions [1].

Several studies have shown that HIV replication is an important determinant of endothelial dysfunction [2–5]. As a consequence of impaired endothelial function, HIV-infected patients may be in a hypercoagulable state [6]. In cross-sectional studies, higher levels of IL-6, an inflammatory cytokine, have been found for HIV-infected compared to uninfected individuals [7,8]. IL-6 has also been shown to be correlated with HIV-RNA levels among patients with advanced HIV [9]. C-reactive protein, a proinflammatory marker, increases over time for patients with HIV, and individuals who progress to AIDS have greater increases [10].

We hypothesized that increased HIV-RNA levels following ART interruption induced activation of tissue factor pathways, thrombosis, and fibrinolysis, and that these inflammatory changes were associated with an increased risk of all-cause mortality.

Prior studies of inflammatory and coagulation markers in HIV-infected patients have not compared those using ART and not using ART in a randomized trial, thereby controlling known and unknown confounding variables. To our knowledge, this is the first planned investigation of inflammatory and coagulation markers with all-cause mortality in HIV-infected patients.

## Methods

The methods and results of the SMART trial have been published [1].

### Study Population

5,472 HIV-infected participants were enrolled at 318 sites in 33 countries. Patients were eligible if they had a CD4+ count over 350 cells/mm^3^ and were willing to initiate, modify, or stop ART as per study guidelines [1]. Participants were asked to consent to storing blood for future research, and only samples from consenting participants were used. The SMART study, including the consent for stored specimens, was approved by the institutional review board at each site and at the University of Minnesota, which served as the Statistical and Data Management Center. The institutional review board at the University of Minnesota also approved plans for analysis of stored specimens for consenting participants.

### Study Treatments

In SMART, patients were randomized to one of two ART strategies. The viral suppression (VS) strategy aimed to maximally suppress viral replication by continuous use of ART. The drug conservation (DC) strategy entailed intermittent use of ART for periods defined by CD4+ count thresholds. Use of ART was stopped (or deferred) until CD4+ count dropped to less than 250 cells/mm^3^, at which time ART was to be (re-)initiated and continued until the CD4+ count rose above 350 cells/mm^3^. Upon confirmation that the CD4+ count was over 350 cells/mm^3^, ART was to be stopped and resumed again when CD4+ count was below 250 cells/mm^3^. During periods of ART use, the goal was to achieve maximal viral suppression.

### Clinical Outcomes

Underlying cause of death was classified using the CoDe system by an Endpoint Review Committee blinded to treatment group [11]. As previously reported, only four of 55 deaths in the DC group and three of 30 deaths in the VS group were attributable to opportunistic diseases (Table S1) [1]. Because no single cause of death dominated the treatment difference, we focus on all-cause mortality in this report.

### Biomarker Substudy

In SMART, follow-up study visits occurred at month 1, month 2, then every 2 mo for the first year, and every 4 mo thereafter. Of the 5,472 participants in SMART, 1,415 in the United States were asked to consent prior to randomization to have plasma stored at baseline (study entry) and at every follow-up visit beginning with the 1-mo visit; 1,361 of these participants consented. For the remaining 4,057 participants, consent was requested at the time of randomization for storing blood at either 4- or 12-mo intervals; 3,800 of these participants consented. Plasma specimens were collected using EDTA (lavender top) blood collection tubes and were shipped frozen to a central repository.

Only specimens obtained prior to 11 January 2006, were used for the analyses described in this paper. As previously reported, on that date investigators and ART-experienced participants were notified of a safety risk in the DC group, enrollment was stopped, and participants in the DC group were advised to restart ART [1].

Four inflammatory markers, high sensitivity C-reactive protein (hsCRP), IL-6, amyloid A, and amyloid P, and two coagulation markers, D-dimer and prothrombin fragment 1+2 (F1.2), were measured by the Laboratory for Clinical Biochemistry Research at the University of Vermont for cases and controls and for a random sample of participants in each treatment group. Biomarkers for the random sample and the case–control study were analyzed in the same batches blinded to treatment group (DC or VS) and case–control status. These six biomarkers were prospectively chosen because they have high laboratory and biological reproducibility [12] and have been associated with all-cause mortality and cardiovascular disease (CVD) in studies of the general population [13–25]. These were the only six biomarkers included in this investigation.

#### Nested case–control study.

**Figure 1:**
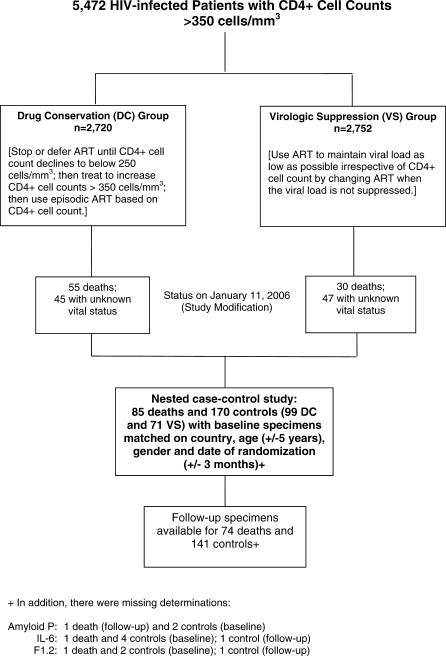
SMART Study Design and Flow Diagram for Case–Control Study

Plasma samples collected at baseline and during follow-up were identified for all who died (85 participants) prior to 11 January 2006, and for two matched controls for each death (170 patients) (Figure 1). Matching was on country, age (± 5 y), sex, and approximate date of randomization (± 3 mo). Age was chosen as a matching variable because of its known association with mortality; sex was chosen for consistency with other case–control studies being done with SMART participants, e.g., with CVD cases, in which sex is an established risk factor; country (site within country where possible) was chosen as a matching variable to control for possible differences in treatment patterns, demographics, and other factors that could vary by site or location; and date of randomization was chosen as a matching variable to ensure that latest levels for cases and controls were measured at approximately the same time following randomization. These matching factors were also chosen because they were clearly not on the causal pathway between the biomarkers and mortality. Controlling for other potential confounding factors was by regression adjustment.

The single follow-up specimen selected for each participant was from the visit before death (latest level). Specimens from the same follow-up visit were used for the matched controls. Follow-up specimens were available for 74 deaths and 141 controls. The median time between measurement of follow-up biomarkers and death was 69 d (25th and 75th percentiles were 40 d and 149 d, respectively).

Nested case–control studies with the same matching criteria were also carried out for all cases of AIDS and for cases of CVD, renal disease, and liver disease. These studies will be reported separately. In selected analyses, controls from these studies were used to supplement the controls chosen for deaths (Figure S1). Specifically, among the 561 controls chosen for deaths, AIDS, CVD, renal, and liver events, four controls randomized at approximately the same time as the death (± 3 mo) were chosen without replacement for each death (340 total).

#### Random sample.

**Figure 2:**
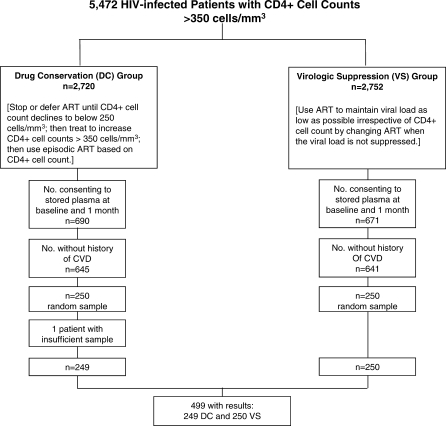
SMART Study Design and Flow Diagram for Random Sample

Specimens collected at study entry and 1 mo after randomization were identified for a random sample of 250 participants from each treatment group who reported no history of CVD at entry (Figure 2). One participant in the DC group who was chosen for analysis did not have an adequate study entry specimen. Thus, analyses are based on 499 participants.

### Statistical Methods

Summary statistics and illustrations appropriate for matched case–control studies were used to summarize key findings [26]. Conditional logistic regression analyses for matched case–control studies were carried out to study the associations of baseline and latest levels for each biomarker with all-cause mortality. Separate analyses by quartile of each biomarker (defined using the random sample) were performed and ORs for each of the three upper quartiles versus the lowest quartile (reference group) are cited along with 95% confidence intervals (CIs) and *p*-values. In addition to models that categorized biomarkers according to quartiles, models with log_10_ transformed biomarker levels were considered, and estimated parameters were used to determine the increase in risk associated with a difference in the biomarker corresponding to its interquartile range (IQR). The IQR was determined from the random sample of 499 study participants. Separate models were fit for each biomarker. The following covariates assessed at study entry were used for the adjusted analyses cited: age, self-reported race (categorized for this analysis as black versus other), use of ART and HIV-RNA level (no ART, versus ART and ≤400 copies/ml, versus ART and >400 copies/ml), CD4+ cell count, smoking status, body mass index (BMI), prior CVD, diabetes, use of blood pressure (BP) medication, use of lipid-lowering medication, total/HDL cholesterol, co-infection with hepatitis B or C, and treatment group.

For some biomarkers very large ORs for the associations with mortality were observed. To investigate the possibility that these large ORs were due to sparse data [27], the number of covariates considered [28], or outliers, four sensitivity analyses were performed: (1) conditional logistic models that included only baseline covariates that were significant at the 0.10 level in a multivariable analysis with mortality (age, prior CVD, co-infection with hepatitis B or C, smoking, and baseline CD4+ cell count); (2) unconditional logistic models; (3) conditional logistic models that excluded outliers, i.e., levels below the lower fence, defined as the lower quartile – 1.5 × IQR or above the upper fence (upper quartile + 1.5 × IQR) [29]; and (4) conditional logistic models that used four controls per case with controls only matched on date of randomization (± 3 mo).

The associations between baseline biomarker levels and mortality were considered for DC and VS participants separately. To assess whether associations between biomarker levels and mortality varied by treatment group, an interaction term (product of log_10_ transformed biomarker and treatment group) was included in the logistic models. Similar methods were used for studying subgroups defined by prior disease history, co-infection with hepatitis, and baseline CD4+ cell count.

For analyses of latest biomarker levels and mortality, log_10_ transformed levels were used, and the study entry level of the biomarker that was the focus of the analysis was included as a covariate with other previously cited covariates. To assess the effects of biomarker differences between the DC and VS groups on the DC/VS OR for mortality, conditional logistic models that included the latest level of the biomarker as well as the treatment indicator were considered. For these analyses, deaths and the four controls matched on date of randomization were used. This expanded case–control study (four instead of two controls) was used because there was a chance difference in the number of DC and VS controls with latest biomarker levels in the case–control study with 1:2 matching. This resulted in an unadjusted DC versus VS difference in mortality that was not as great as previously reported [1]. Because the focus was on the risk of death in the DC versus VS group, a comparison protected by randomization, matching was limited to date of randomization to ensure latest biomarker levels were measured at approximately the same time for each death and the four matched controls.

The association between each biomarker and other covariates at study entry was studied using linear regression analysis. Biomarker changes after 1 mo were compared for DC and VS participants using analysis of covariance. The association of biomarker change (after log_10_ transformation) and HIV-RNA change at 1 mo for DC participants with an HIV-RNA level ≤400 copies/ml at study entry was studied using linear regression analysis.

Statistical analyses were performed using SAS (Version 9.1) [30]. All reported *p*-values are two-sided.

## Results

### Case–Control Sample: Baseline Biomarkers and All-Cause Mortality

Most of the deaths (79 of 85) occurred in the US. Sites in the US began enrollment 2 to 3 y before most sites in other countries and accounted for 55% of the randomized participants. In univariate analyses, cases and controls differed with respect to age (*p* = 0.007), baseline CD4+ count (*p* = 0.03), co-infection with hepatitis B or C (*p* = 0.0008), smoking status (*p* = 0.0001), diabetes (*p* = 0.03), use of BP-lowering treatment (*p* = 0.02), and prior CVD (*p* = 0.04) (Table 1). In multivariate analyses that included the baseline covariates described in Methods for adjusted analyses, age (*p* = 0.02), smoking (*p* = 0.01), prior CVD (*p* = 0.04), co-infection with hepatitis B or C (*p* = 0.03), and baseline CD4+ cell count (*p* = 0.10) were associated (*p* = 0.10 or lower) with mortality.

**Table 1:**
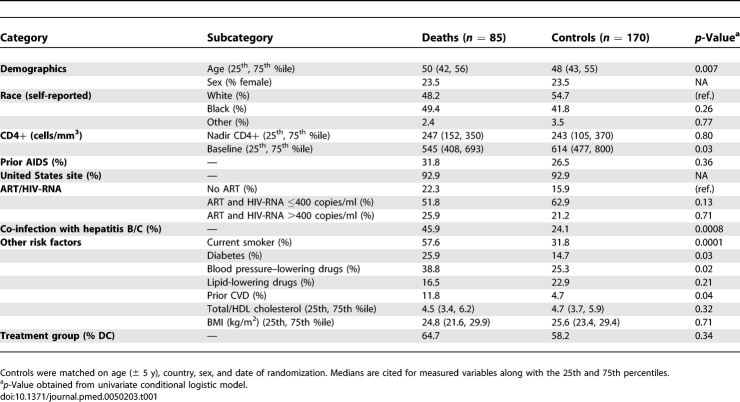
Characteristics of Deaths and Matched Controls at Study Entry

With the exception of amyloid P, levels of the inflammatory markers were higher in deaths than matched controls (Table 2, “study entry”). Differences between deaths and controls for IL-6 and D-dimer were highly significant (*p* < 0.0001); hsCRP was also significantly higher among deaths than controls (*p* = 0.005).

**Table 2:**
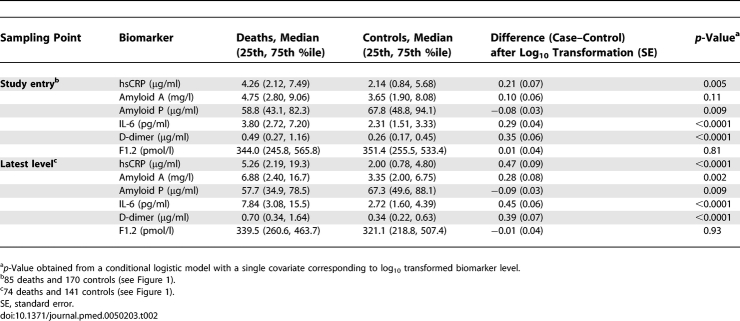
Study Entry and Latest Levels of Six Biomarkers for Deaths and Matched Controls

Figures S2–S7 display biomarker levels for each triad (death and two matched controls) ordered by the biomarker level for the death. For IL-6 (Figure S5) and D-dimer (Figure S6), and to a lesser extent hsCRP (Figure S2), there is a shift in the distribution for deaths (red circles) compared to controls (blue circles). For other biomarkers, control levels are lower than deaths with high levels and higher than deaths with low levels (in part, a reflection of the regression to the mean phenomenon).

For both IL-6 and D-dimer, the discordance of cases and matched controls in the lower quartile (<25th percentile) and upper quartile (>75th percentile) was striking. For D-dimer, there were 23 controls with levels in the lowest quartile (<0.18 μg/ml) that were matched with deaths that had levels in the upper quartile. In contrast, there were only two controls with D-dimer levels in the upper quartile matched with deaths in the lowest quartile. For IL-6, there were 25 controls with levels in the lowest quartile (<1.6 pg/ml) matched with deaths that had levels in the upper quartile, and three controls with IL-6 in the upper quartile matched with deaths in the lowest quartile.

Strong risk gradients with mortality were evident for both IL-6 and D-dimer (Table 3). For IL-6, unadjusted ORs (for each of the three upper quartiles versus lowest) were 8.3 (95% CI, 3.3–20.8), 3.2 (95% CI, 1.3–7.9), and 1.3 (95% CI, 0.5–3.6). For D-dimer corresponding ORs were 12.4 (95% CI, 4.2–37.0), 4.0 (95% CI, 1.3–12.3), and 3.2 (95% CI, 1.1–9.0). In models that considered these biomarkers as continuous variables after log_10_ transformation, a difference corresponding to the IQR was associated with an OR of 3.4 (95% CI, 2.2–5.4) for IL-6 and 3.9 (95% CI, 2.3–6.6) for D-dimer. Covariate adjustment tended to strengthen these associations (Table 3), and sensitivity analyses yielded consistent findings (Table S2). Similar analyses with the 1:4 matching yielded results with reduced, but still very large, ORs for IL-6 and D-dimer (Table S3). For example, unadjusted ORs (upper quartile versus lowest) were 6.1 (95% CI, 2.7–13.6) and 6.6 (95% CI, 2.9–14.9) for IL-6 and D-dimer, respectively.

**Table 3:**
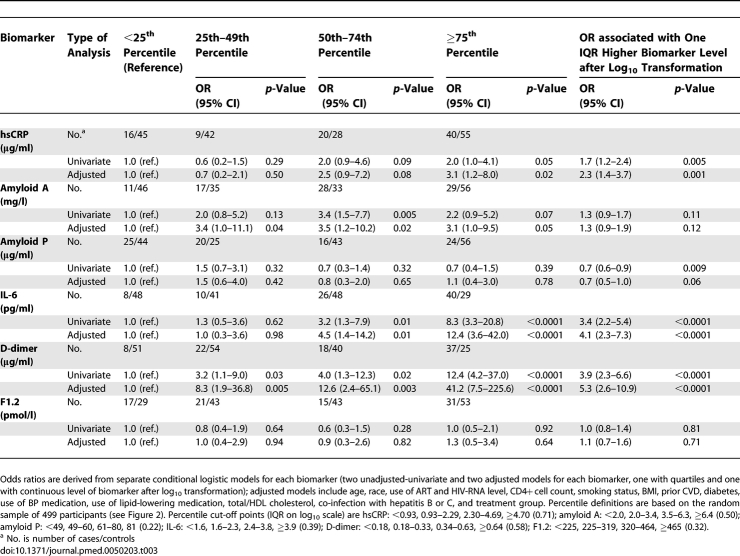
Risk of Death Associated with Biomarker Levels at Study Entry

Significant associations between study entry levels of hsCRP and amyloid P and mortality were also evident. Risk of death increased with increasing levels of hsCRP but not as strongly as for IL-6 and D-dimer. The unadjusted OR (upper versus lower quartile of hsCRP) was 2.0 (95% CI, 1.0–4.1). Higher levels of amyloid P were associated with a lower risk of death in analyses based on the continuous biomarker level, but no apparent trend by quartile was evident. This association was only of borderline significance after covariate adjustment and may be due in part to outliers. After excluding outliers (ten deaths and seven controls), the OR associated with a one IQR higher level of amyloid P was 1.1 (95% CI, 0.7–1.7) (Table S2).

When IL-6 and D-dimer were considered together in the same model they each remained significantly associated with all-cause mortality. Unadjusted ORs for the highest versus lowest quartile were 4.7 (95% CI, 1.7–12.7; *p* = 0.002) for IL-6 and 6.1 (95% CI, 2.0–18.6; *p* = 0.001) for D-dimer. ORs for a difference corresponding to the IQR were 2.7 (95% CI, 1.6–4.4; *p* < 0.0001) for IL-6 and 2.6 (95% CI, 1.5–4.6; *p* = 0.001) for D-dimer.

In the analyses described above, the DC and VS groups were combined. In separate analyses for each group (Table 4), associations between biomarker levels at baseline and mortality were similar (Table 3, note last two columns for comparison to models that considered both treatment groups combined). For DC participants, median levels (expressed as deaths, controls) were 4.49, 1.78 μg/ml for hsCRP; 3.85, 2.24 pg/ml for IL-6; and 0.63, 0.22 μg/ml for D-dimer. For VS participants, these median levels were 3.60, 3.07 μg/ml for hsCRP; 3.78, 2.43 pg/ml for IL-6; and 0.37, 0.29 μg/ml for D-dimer.

**Table 4:**
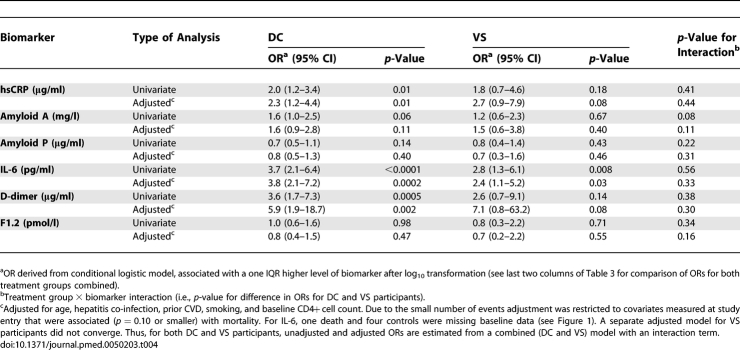
Risk of Death Associated with Biomarker Levels at Study Entry for the Drug Conservation (DC) and Viral Suppression (VS) Treatment Groups

Likewise, associations were similar for those with prior cancer, CVD, renal or liver disease (31% of deaths and 16% of controls), and those with no history of these conditions (*p* = 0.33 for hsCRP, *p* = 0.88 for IL-6, and *p* = 0.74 for D-dimer interactions); for those co-infected with hepatitis or not (*p* = 0.37 for hsCRP, *p* = 0.41 for IL-6, and *p* = 0.76 for D-dimer interactions); and for those with a baseline CD4+ cell less than 600 cells/mm^3^ (approximate median) and 600 cells/mm^3^ or more(*p* = 0.85 for hsCRP, *p* = 0.21 for IL-6, and *p* = 0.41 for D-dimer interactions).

Associations with hsCRP, Il-6, and D-dimer were also similar for deaths in the first year (38 deaths) and after the first year (47 deaths). During the first year, the OR corresponding to the IQR for hsCRP, IL-6, and D-dimer were 1.5 (95% CI, 0.9–2.4; *p* = 0.16), 2.5 (95% CI, 1.4–4.3; *p* = 0.002), and 3.9 (95% CI, 1.9–8.0; *p* = 0.0003), respectively. For deaths occurring after the first year, ORs for hsCRP, IL-6, and D-dimer were 1.9 (95% CI, 1.1–3.2; *p* = 0.01), 5.0 (95% CI, 2.4–10.4; *p* < 0.0001), and 3.9 (95% CI, 1.8–8.4; *p* = 0.0005).

Deaths due to substance abuse (eight deaths) or to accidents, violence, or suicide (seven deaths) could attenuate the associations between the biomarkers and mortality. Thus, analyses were carried out excluding these 15 deaths. Unadjusted ORs corresponding to a difference equal to the IQR were 1.9 (95% CI, 1.2–2.8) for hsCRP, 3.7 (95% CI, 2.2–6.3) for IL-6, and 4.0 (95% CI, 2.1–7.4) for D-dimer. Each of these ORs is larger by a small amount compared to the corresponding estimates in Table 3. Twenty-one deaths were classified as CVD or were unwitnessed. Unadjusted ORs of CVD or unwitnessed death corresponding to a difference equal to the IQR were 2.3 (95% CI, 1.0–5.0; *p* = 0.04) for hsCRP, 3.2 (95% CI, 1.2–8.4; *p* = 0.02) for IL-6, and 3.2 (95% CI, 1.1–9.3; *p* = 0.04) for D-dimer.

### Case–Control Sample: Change in Biomarkers and All-Cause Mortality

Table 2 (“latest level”) compares latest levels of each biomarker for deaths and matched controls (Table S4 gives similar results for the 1:4 matching). In these univariate analyses, significant differences were observed for all of the biomarkers except F1.2. Deaths had higher latest levels than controls, except amyloid P for which latest levels were lower for deaths than controls. Findings were strongest for hsCRP, IL-6, and D-dimer. Average differences between deaths and controls were greater for latest levels than for study entry levels (Table 2 “study entry” and Table S4).

Table 5 gives adjusted ORs for latest levels of each biomarker. Adjusted ORs corresponding to a difference equal to the IQR were 2.4 (95% CI, 1.4–4.2) for hsCRP, 2.0 (95% CI, 1.2–3.1) for IL-6, and 2.2 (95% CI, 1.1–4.1) for D-dimer. These models also included study entry levels of each biomarker. Study entry levels of IL-6 and D-dimer remained significantly associated with all-cause mortality after consideration of latest levels (*p* = 0.0008 for IL-6 and *p* = 0.003 for D-dimer). After considering latest level of hsCRP, the study entry level was not significant (*p* = 0.07). In models that also included latest levels of HIV-RNA and CD4+ cell count, neither of which were significantly associated with all-cause mortality, these associations were diminished slightly but remained significant. For latest levels of hsCRP, IL-6, and D-dimer, the ORs were 2.2 (*p* = 0.009), 1.7 (*p* = 0.04), and 2.0 (*p* = 0.04).

**Table 5:**
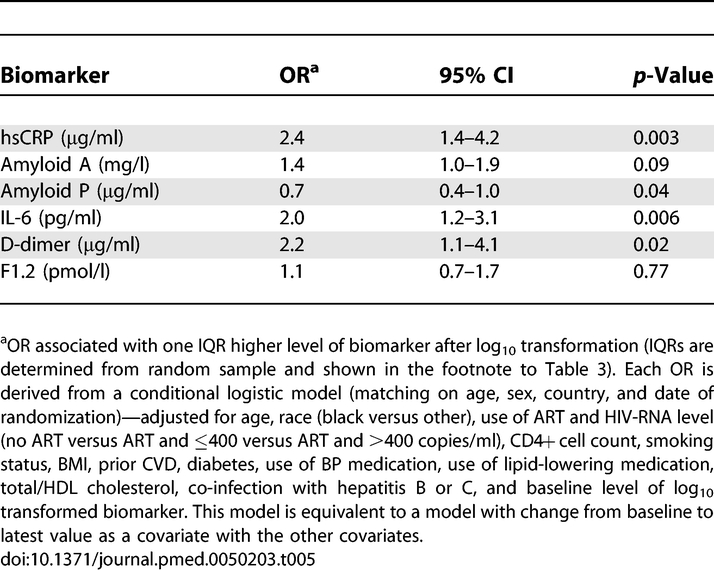
Risk of Death Associated with Latest Level of Each Biomarker

### Random Sample: Associations at Baseline

As previously reported, the majority of patients in SMART were using ART at entry [1]. In the random sample, 74% were using ART; among those using ART, 71% had an HIV-RNA level 400 copies/ml or lower. Approximately 6% of patients had not previously used ART; the remainder of those not using ART had discontinued it prior to enrolling in SMART. In the random sample, treatment groups were well balanced (Table 6).

**Table 6:**
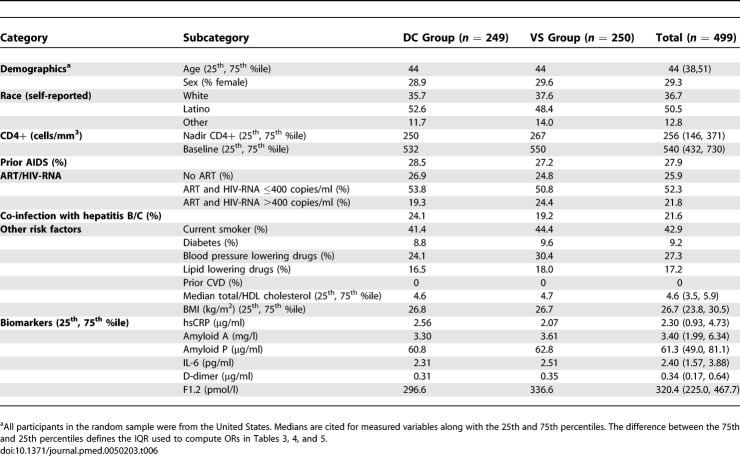
Characteristics of Random Sample of DC and VS Participants at Study Entry

Multiple regression analyses of each biomarker (after log_10_ transformation) on baseline covariates were performed. The covariates used in the regression analyses were the same as those used in the adjusted case–control analysis. An exception was history of CVD since no one in the random sample had a history of CVD (Figure 2). D-dimer was significantly higher for those not on ART than for those on ART with an HIV-RNA level 400 copies/ml or lower (0.15 on log_10_ scale; *p* = 0.0007) and for those on ART with an HIV-RNA over 400 copies/ml (0.12; *p* = 0.02). Other biomarkers did not vary significantly according to use of ART and HIV-RNA level at study entry.

Smoking and co-infection with hepatitis B or C, which were both significantly related to mortality, were not significantly associated with either IL-6 (*p* = 0.19 for smoking and *p* = 0.16 for co-infection) or D-dimer (*p* = 0.64 for smoking and *p* = 0.39 for co-infection) at baseline. Smoking was not significantly associated with any of the biomarkers. hsCRP was lower by 0.217 μg/ml after log_10_ transformation (*p* = 0.0001) and amyloid P was lower by 0.068 (*p* = 0.0005) for those who were co-infected with hepatitis; co-infection was not associated with the other biomarkers.

Significant predictors of log_10_ IL-6 were age (0.069 higher with each 10 y in age; *p* < 0.0001) and BMI (0.009 higher with each kg/m^2^ greater BMI; *p* = 0.0004). For log_10_ D-dimer, in addition to ART and HIV-RNA level, levels were greater among older participants (0.062 higher with each 10 y; *p* = 0.002), for black participants (0.123; *p* = 0.0009), for participants with diabetes (0.161; *p* = 0.01), and for those with greater BMI (0.008 per unit higher; *p* = 0.02). D-dimer levels were lower for men (−0.151; *p* = 0.0003) and for those with higher CD4+ cell counts (−0.023 per 100 cells/mm^3^ higher; *p* = 0.002). To put these changes in perspective, the IQRs on the log_10_ scale for IL-6 and D-dimer were 0.39 pg/ml and 0.58 μg/ml, respectively. Differences as large as the IQR for each marker were associated with 3- to 4-fold greater risks of all-cause mortality.

### Random Sample: Treatment Differences at 1 mo

Table 7 shows average changes in log_10_ transformed biomarker levels 1 mo after randomization. IL-6 and D-dimer increased significantly (*p* = 0.0005 and *p* < 0.0001, respectively) from study entry to 1 mo in the DC group compared to the VS group (*p* < 0.0001). Considering the non-transformed levels, the median increase in D-dimer for DC patients was 0.05 μg/ml (a 16% increase); IL-6 increased by 0.60 pg/ml in the DC group (a 30% increase). For VS patients, the median increases were 0.0 μg/ml and 0.12 pg/ml (a 5% increase) for D-dimer and IL-6, respectively. Changes in hsCRP and amyloid A were in the same direction—greater increases for DC compared to VS patients—but did not differ significantly between treatment groups.

**Table 7:**
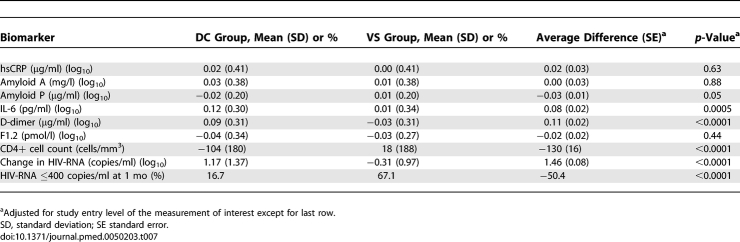
Biomarker, CD4+ Cell Count Change, and HIV-RNA Level Change 1 mo after Randomization

For both IL-6 and D-dimer, treatment differences were greater for patients who were on ART at entry and had HIV-RNA levels 400 copies/ml or below. For this subgroup (52% of patients), D-dimer increased in the DC group by 0.07 μg/ml (a 27% increase) and declined in the VS group by −0.02 μg/ml (*p* < 0.0001 for treatment difference). Similarly, for IL-6, the median increases for DC and VS patients were 0.98 (a 43% increase) and 0.08 pg/ml, respectively (*p* < 0.0001 for difference).

**Figure 3:**
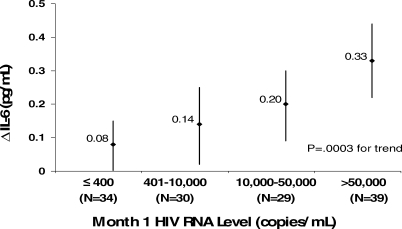
Change in Log_10_ IL-6 (pg/ml) from Baseline to 1 mo According to HIV-RNA Level at 1 mo for Participants in the Drug Conservation (DC) Group (CD4+ Guided Intermittent ART) with an HIV-RNA level 400 Copies/ml or Less at Baseline

**Figure 4:**
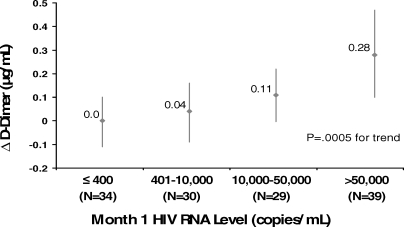
Change in Log_10_ D-dimer (μg/ml) from Baseline to 1 mo According to HIV-RNA Level at 1 mo for Participants in the Drug Conservation (DC) Group (CD4+ Guided Intermittent ART) with an HIV-RNA level 400 Copies/ml or less at Baseline

The changes in IL-6 and D-dimer for the subgroup of DC patients with HIV-RNA levels 400 copies/ml or below were further examined according to HIV-RNA levels at 1 mo. Following ART interruption, biomarker increases were greater for those with higher HIV-RNA levels at 1 mo (Figures 3 and 4).

Results in Table 5 were used to estimate the potential impact of treatment differences on mortality. For IL-6, the DC/VS difference after 1 mo on the log_10_ scale was 0.08 pg/ml. Based on the regression analysis cited in Table 5, a difference of this magnitude is associated with a 16% increased risk of death (95% CI, 10%–25%). Similarly, the 0.11 log_10_ higher level of D-dimer for DC compared to VS participants is associated with a 24% (95% CI, 13%–46%) increased risk of death.

### Impact of Adjustment for Latest Levels of IL-6 and D-dimer on OR for DC Versus VS for All-Cause Mortality

Matched logistic models were used to assess the effect of adjusting for latest levels of IL-6 and D-dimer on the DC/VS OR for death. In the model with two controls per case, the unadjusted OR for the DC versus the VS group was 1.3 (95% CI, 0.8–2.2). Because this OR was considerably lower than the hazard ratio previously reported for all-cause mortality [1], we explored reasons for it and created an expanded case–control data set. A chance imbalance in the number of DC and VS participants selected as controls is the reason the OR was lower. Among the 170 controls, 99 were in the DC group and 71 were in the VS group. The expected number was 85 in each group. With the expanded case–control study (four controls for each death), the unadjusted DC/VS OR for all-cause mortality was 1.8 (95% CI, 1.1–3.1). This estimate is identical to that previously reported [1]. With adjustment for latest level of IL-6, the OR was 1.5 (95% CI, 0.8–2.8); with adjustment for latest level of D-dimer the OR was 1.4 (95% CI, 0.8–2.5).

We also considered the effect of adjusting for both study entry and latest levels of IL-6 and D-dimer and of adjusting for HIV- RNA and CD4+ cell count on the DC/VS OR (Table S5). Similar to an earlier report, adjustment for CD4+ cell count had a greater effect on the OR (DC/VS) for mortality (OR = 1.2; 95% CI, 0.7–2.2) than adjustment for latest HIV-RNA levels (OR = 1.6; 95% CI, 0.9–2.9) [1]. With adjustment for latest levels of IL-6, D-dimer, CD4+ cell count, and HIV-RNA, the OR (DC/VS) was 1.3 (95% CI, 0.6–2.6).

## Discussion

Elevated levels of either IL-6 or D-dimer at study entry were strongly related to all-cause mortality in the case–control study. In the random sample, both D-dimer and IL-6 increased in the DC group compared to the VS group, particularly in the large subgroup on ART at entry with a suppressed HIV-RNA level. Increases in both markers in the DC group were related to the level of HIV-RNA after 1 mo. Finally, increases in these markers following randomization were associated with mortality. Taken together, these findings suggest that HIV-induced activation of inflammatory and coagulation pathways has an adverse effect on all-cause mortality among patients with relatively preserved CD4+ counts, and that interrupting ART may further increase this risk by raising IL-6 and D-dimer levels. Further research on the relationship of these biomarkers with mortality and morbidity in treated and untreated HIV-infected individuals is warranted.

The associations between IL-6 and D-dimer levels at study entry with all-cause mortality were much stronger than in previous studies of non-HIV-infected populations that usually focused on CVD morbidity and mortality [13,14,16–18,20–22,31]. While these strong associations persisted in a number of different analyses, including subgroups defined by hepatitis co-infection, baseline CD4+ cell count, and prior disease history, the relatively small number of deaths considered here compared to studies in the general population suggest that the results should be interpreted with caution and require confirmation. Findings for deaths attributed to CVD or unwitnessed deaths were consistent with those for all-cause mortality, although the number of these deaths was small. The relationship of these biomarkers with CVD morbidity and mortality will be the subject of a separate report.

Patients in SMART were relatively healthy and did not have advanced HIV disease. Deaths were attributed to a variety of causes and were largely not due to AIDS, as would be expected in a cohort of patients with CD4+ counts that averaged about 600 cells/mm^3^ at entry (Table S1) [32]. Associations of D-dimer and IL-6 with all-cause mortality have been reported in other studies that included non-HIV-infected participants [15,23,33].

Relationships between elevated IL-6 and D-dimer levels and all-cause mortality were strong in both DC and VS patients. However, our findings for patients on continuous ART (VS group) require validation, as mortality rates were low. However, if substantiated, and because most patients in the VS group had HIV-RNA levels of 400 copies/ml or less at study entry, ongoing activation of these pathways may exist even in the presence of effective ART, consistent with data demonstrating ongoing viral replication, even in patients with HIV-RNA of less than 50 copies/ml [34]. Considering our finding that ART is associated with lower levels of these biomarkers, more aggressive ART that might lower IL-6 and D-dimer may warrant further investigation.

Increases in hsCRP, IL-6, and D-dimer from study entry to the visit preceding the death were associated with an increased risk of death. Whether the activation of tissue factors secondary to inflammation is the key event is likely but unproven in this study. Several alternative hypotheses are possible. One study showed that tissue factor, an initiator of coagulation, is generated by HIV-related proteins and could have pathologic effects [35]. Another study found that HIV-infected leukocytes transmigrate across the endothelium [36], and this dissemination of virus could result in damage to multiple organs. Alternatively, circulating lipopolysaccharide, which has been shown to be higher in HIV-infected compared to uninfected individuals [37], induces tissue factor transcription, which in turn decreases F1.2 and soluble fibrin, resulting in fibrin split products such as D-dimer [38]. Lipopolysaccharide also activates monocytes to produce inflammatory cytokines, including IL-6 [39]. It is also possible that elevations of inflammatory markers, such as hsCRP and IL-6, and of D-dimer are independent events. In analyses that considered the joint influence of IL-6 and D-dimer, each remained strongly associated with all-cause mortality. Specific therapies that reduce the inflammatory response to HIV and decrease hsCRP, IL-6, and D-dimer levels may warrant investigation as an approach for reducing risk of death among HIV-infected individuals [40–42].

ART interruption resulted in increases in IL-6 and D-dimer, therefore the effect of these treatment differences on the DC/VS OR for mortality was explored. Adjustment for follow-up (latest) levels of IL-6 and D-dimer resulted in a modest reduction of the DC/VS OR for mortality, supporting the hypothesis that the excess risk of death in the ART interruption group may be explained by biological mechanisms for which IL-6 and D-dimer are markers.

There are some limitations in our work. Confidence intervals for ORs were wide due to the small number of deaths. While we selected six biomarkers from a large number of possible markers based on previous work in non-HIV-infected populations, some associations may have resulted from chance. This is less likely for the associations of IL-6 and D-dimer levels at study entry with all-cause mortality, which were highly significant. Cost considerations limited the number of controls for each death for which we could assess biomarkers. This reduced the statistical power to detect associations with mortality. The limited matching carried out, particularly for the expanded case–control study with four controls, may have resulted in incomplete control of confounding factors. However, regression adjustment for a number of covariates did not alter our overall conclusions. We measured latest levels at the visit immediately prior to the event of interest. For biomarker levels proximal to death, it is possible that reverse causality (i.e., an already present disease process caused the increase in the biomarker instead of vice versa) may explain these findings. In addition, follow-up specimens were not available for 11 deaths and 29 controls, and this limited our ability to assess the prognostic importance of biomarker changes on mortality. Finally, while we did not find evidence for different associations with mortality for DC compared to VS patients, the number of events experienced by VS patients was small, limiting the power for those comparisons. Similarly, power for other subgroup analyses considered was also low.

In summary, elevated levels of D-dimer and IL-6 identify HIV-infected patients at high risk of death. The magnitude of the association is clinically relevant and reasons for it require further study.

## Supporting Information

Figure S1 SMART Study Design and Flow Diagram for Case–Control Study with 1:4 Matching for Deaths and Controls Matching was on date of randomization to ensure latest levels for deaths, and controls were determined at similar time point following randomizations.

Figure S2 Baseline Level of hsCRP (μg/ml) for Each of 85 Death–Control Triads Ordered by hsCRP Level of Deaths Plotted on Logarithmic Scale (Log_10_) Solid lines give median levels at baseline for deaths (4.3 μg/ml) and controls (2.1 μg/ml). Red circles and lines are for deaths, and blue circles and lines are for controls.

Figure S3 Baseline Level of Amyloid A (mg/l) for Each of 85 Death–Control Triads Ordered by Amyloid A Level of Deaths Plotted on Logarithmic Scale (Log_10_) Solid lines give median levels at baseline for deaths (4.75 mg/l) and controls (3.65 mg/l). Red circles and lines are for deaths, and blue circles and lines are for controls.

Figure S4 Baseline Level of Amyloid P (μg/l) for Each of 85 Death–Control Triads Ordered by Amyloid P Level of Deaths Plotted on Logarithmic Scale (Log_10_) Solid lines give median levels at baseline for deaths (58.8 μg/l) and controls (67.8 μg/l). Red circles and lines are for deaths, and blue circles and lines are for controls.

Figure S5 Baseline Level of IL-6 (pg/ml) for Each of 84 Death–Control Triads Ordered by IL-6 Level of Deaths Plotted on Logarithmic Scale (log_10_) IL-6 level was missing for one death. Solid lines give median levels at baseline for deaths (3.8 pg/ml) and controls (2.3 pg/ml). Red circles and lines are for deaths, and blue circles and lines are for controls.

Figure S6 Baseline Level of D-dimer (μg/ml) for Each of 85 Death–Control Triads Ordered by D-dimer Level of Deaths Plotted on Logarithmic Scale (log_10_) Solid lines give median levels at baseline for deaths (0.49 μg/ml) and controls (0.26 μg/ml). Red circles and lines are for deaths, and blue circles and lines are for controls.

Figure S7 Baseline Level of F1.2 (pmol/l) for Each of 84 Death–Control Triads Ordered by F1.2 Level of Deaths Plotted on Logarithmic Scale (Log_10_) F1.2 level was missing for one death. Solid lines give median levels at baseline for deaths (344.0 pmol/l) and controls (351.4 pmol/l). Red circles and lines are for deaths, and blue circles and lines are for controls.

Table S1 Underlying Cause of Death for Participants in SMART

Table S2 Odds Ratio Associated with a One IQR Higher Level of Biomarker at Study Entry: Three Models with Log_10_-Transformed Biomarker

Table S3 Risk of Death Associated with Biomarker Levels at Baseline—Four Controls Per Death

Table S4 Study Entry and Latest Levels of Six Biomarkers for Deaths and Matched Controls (Four Matched Controls for Each Death)

Table S5 Estimates of the Odds Ratio for DC Versus VS Participants for All-Cause Mortality After Adjustment for Study Entry and Latest Levels of IL-6, D-dimer, CD4+ Cell Count, and HIV RNA Level

Text S1 CONSORT Checklist

Text S2 Protocol

## Abbreviations

ART: antiretroviral treatment
BP: blood pressure
CVD: cardiovascular disease
DC: drug conservation
F1.2: prothrombin fragment 1+2
hsCRP: high sensitivity C-reactive protein
IL-6: interleukin-6
IQR: interquartile range
OD: opportunistic disease
OR: odds ratio
SMART: Strategies for Management of Anti-Retroviral Therapy
VS: viral suppression
