# Supplementary material for: Inflammatory and Coagulation Biomarkers and Mortality in Patients with HIV Infection
Source: PLoS Med. 2008 Oct 21;5(10):e203. doi: 10.1371/journal.pmed.0050203 (PMC2570418; doi:10.1371/journal.pmed.0050203)
Supplement: Supplementary file 4 [file pmed.0050203.sg005.pdf]

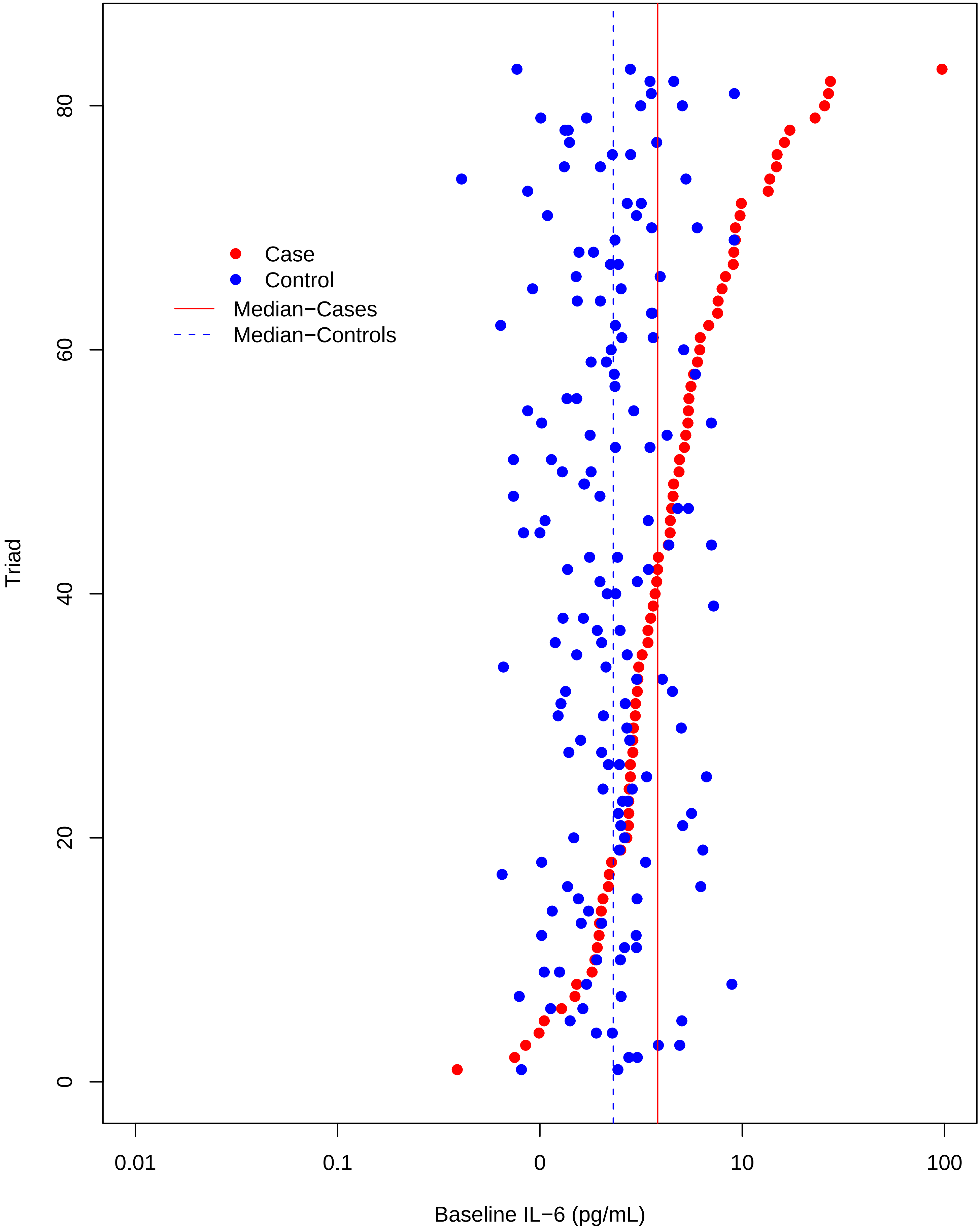

Case
Control
Median−Cases
Median−Controls
Triad
0
20
40
60
80
0.01
0.1
0
10
100
Baseline IL−6 (pg/mL)
