# Supplementary material for: Inflammatory and Coagulation Biomarkers and Mortality in Patients with HIV Infection
Source: PLoS Med. 2008 Oct 21;5(10):e203. doi: 10.1371/journal.pmed.0050203 (PMC2570418; doi:10.1371/journal.pmed.0050203)
Supplement: Supplementary file 8 [file pmed.0050203.sd002.doc]

A Large, Simple Trial Comparing Two

**S**trategies for **M**anagement of **A**nti-**R**etroviral **T**herapy

(The SMART Study)

A Multicenter Study of the

Terry Beirn Community Programs for Clinical Research on AIDS

Sponsored by:

The National Institute of Allergy and Infectious Diseases

Division of AIDS

Protocol Team:	Wafaa El-Sadr, M.D., M.P.H., Co-Chair

James Neaton, Ph.D., Co-Chair

Philip Andrew, R.N., B.S.

Abdel Babiker, Ph.D.

William Burman, M.D.

Calvin Cohen, M.D., M.Sc.

David Cohn, M.D.

David Cooper, AO, M.D., D.Sc.

Fraser Drummond, MBChB MRCA DA (UK)

Fred Gordin, M.D.

Birgit Grund, Ph.D., Senior Statistician

Richard Hafner, M.D., DAIDS Clinical Representative

Carlton Hogan

Jennifer Hoy, M.B.B.S. F.R.A.C.P.

Karin L. Klingman, M.D., DAIDS Clinical Representative

Jay Kostman, M.D.

Jens Lundgren, M.D., DMSc

Ana Martinez, R.Ph., Protocol Pharmacist

Carol Miller, M.P.H., Protocol Manager

Christopher Mullin, M.S., Protocol Manager

Jacqueline Neuhaus, M.S., Protocol Manager

Mollie Poehlman, M.S., Protocol Manager

Ronald J. Prineas, M.D., Ph.D.

Claire Rappoport, M.A., Community Representative

Barry Schmetter, B.S., Clinical Trials Specialist

Janis Stewart, R.N., CSMG Representative

**Other Contributors**

Body Composition

Andrew Carr, M.D., Co-Chair, University of New South Wales, Sydney, Australia

Fehmida Visnegarvala, M.D., Co-Chair, Houston AIDS Research Team, Houston, TX

Glenn Bartsch, Sc.D., School of Public Health, University of Minnesota, Minneapolis, MN

Kenneth Ellis, Ph.D., Baylor College of Medicine, Houston, Texas

Cynthia Gibert, M.D., Washington Regional AIDS Program, Washington, DC

Subha Raghavan, Ph.D., Harlem AIDS Treatment Group, New York, NY

Judy Shlay, M.D., Denver Public Health Department, Denver, CO

Quality of Life

William Burman, M.D., Co-Chair, Denver Public Health Department, Denver, CO

Albert Wu, M.D., M.P.H., Co-Chair, Johns Hopkins School of Medicine, Baltimore, MD

Cost-Effectiveness

William Burman, M.D., Co-Chair, Denver Public Health Department, Denver, CO

Sherry Glied, Ph.D., Co-Chair, Mailman School of Public Health, Columbia Univ., New York, NY

David Bloom, Ph.D., School of Public Health, Harvard University, Boston, MA

Joshua Zivin, Ph.D., Mailman School of Public Health, Columbia Univ., New York, NY

HIV Transmission Risk Behavior

William Burman, M.D., Chair, Denver Public Health Department, Denver, CO

John Douglas, M.D., Denver Public Health Department, Denver, CO

Gerald Friedland, M.D., School of Medicine, Yale University, New Haven, CT

Kees Rietmeijer, M.D., Denver Public Health Department, Denver, CO

HIV Drug Resistance

Jay Kostman, M.D., Co-Chair, Philadelphia FIGHT, Philadelphia, PA

Tom Merigan, M.D., Co-Chair, Stanford, Palo Alto, CA

John Baxter, M.D., Southern New Jersey AIDS Clinical Trials, Camden, NJ

Mark Wainberg, M.D., CTN/Lady Davis Research Institute, Montreal, Canada

Adherence

Gerald Friedland, M.D., Co-Chair, School of Medicine, Yale University, New Haven, CT

Sharon Mannheimer, M.D., Co-Chair, Harlem AIDS Treatment Group, New York, NY

Table of Contents

1.	Synopsis	1

2.	Background and Rationale	4

2.1.	Treatment Guidelines	4

2.2.	The Strategies for Management of Antiretroviral Therapy (SMART) Study	5

2.3.	Rationale for a Drug Conservation (DC) Treatment Strategy	6

2.3.1.	HIV Disease Progression Is Rare among Patients with High CD4+ Cell Counts	6

2.3.2.	Proximal CD4+ Cell Count Is a Better Predictor of Disease Progression than Proximal HIV RNA Level	7

2.3.3.	Symptoms May Precede Immunologic Decline	10

2.3.4.	Immunologic Competency Can Be Recovered in Patients with CD4+ Cell Count Rebounds	10

2.3.5.	Durability of Viral Suppression with HAART Is Similar Among Patients with CD4+ Cell Counts 200 to 349 cells/mm^3^ and CD4+ Cell Counts ≥ 350 cells/mm^3^	11

2.3.6.	Durability of Viral Suppression with Currently Available HAART Is Limited	11

2.3.7.	Risk of Resistance Increases with Drug Pressure and May Even Occur in the Presence of Viral Suppression	11

2.3.8.	Long-Term Use of Antiretroviral Drugs is Associated with Unintended Side Effects	12

2.3.9.	Adherence to Antiretroviral Therapy Is Difficult	13

2.4.	Rationale for a Virologic Suppression (VS) Strategy	13

2.4.1.	Baseline HIV RNA Level Predicts Progression to AIDS in Untreated Participants	13

2.4.2.	Regimens that Provide Maximal Suppression Are Associated with a Lower Risk of Resistance	14

2.4.3.	Changing Regimens at Lower Viral Loads Is More Likely to Result in Viral Suppression	15

2.4.4.	Treatment Interruptions Result in Increases in Viral Load and CD4+ Cell Count Declines	15

2.4.5.	Viral Suppression Is More Durable among Patients Who Start Therapy at Higher CD4+ Cell Counts	16

2.4.6.	Many Antiretroviral Drugs Are Available	17

2.4.7.	Nadir of CD4+ Cell Count Predicts Risk of Opportunistic Diseases	17

2.5.	Benefits and Risks of Each Strategy	18

2.5.1.	Benefits and Risks of the Drug Conservation (DC) Strategy	18

2.5.2.	Benefits and Risks of the Virologic Suppression (VS) Strategy	18

2.6.	Rationale for Selected Study Design	19

2.6.1.	The Need for Two Distinct and Sufficiently Different Treatment Strategies	19

2.6.2.	The Need for Primary and Secondary Clinical Endpoints	20

2.6.3.	The Need for Long-Term Follow-up	20

2.6.4.	The Need for a Trial to Detect Moderate Differences in Clinical Outcomes between the Treatment Strategies	20

2.6.5.	The Need for a Study with Broad Eligibility Criteria	21

2.6.6.	The Need for Simple, Focused Data Collection and for Substudies to Aid in Understanding Treatment Differences	21

2.6.7.	The Need for the Trial to Accommodate New Agents and New Classes of Antiretroviral Agents during the Study	23

2.7.	Preliminary Assessment of Percentage of Patients with HIV that Are Potentially Eligible	24

2.8.	Summary of Rationale and Primary Hypothesis of the SMART Study	24

2.9.	Agents Used in the SMART Study	24

2.10.	Purpose	25

3.	Methodology	26

3.1.	Study Design	26

3.2.	Study Objectives	27

3.2.1.	Primary Objective	27

3.2.2.	Secondary Objectives	27

3.2.3.	Tertiary Objectives	28

3.3.	Study Endpoints	29

3.3.1.	Primary Endpoint	29

3.3.2.	Other Major Endpoints	29

3.4.	Randomization	31

3.5.	Sample Size and Statistical Considerations	31

3.6.	Patient Selection	33

3.6.1.	Inclusion Criteria	33

3.6.2.	Exclusion Criteria	34

3.7.	Study Plan	34

3.7.1.	Treatment Management Guidelines at Randomization	34

3.7.2.	Treatment Management Guidelines During Follow-up	35

3.7.3.	Baseline Screening and Enrollment	38

3.7.4.	Patient Follow-up	40

4.	Clinical Management Issues	44

4.1.	Choice of Antiretroviral and Other Treatments	44

4.2.	Resistance Testing to Guide Antiretroviral Therapy Changes	44

4.3.	CD4+ Cell Count and Viral Load Level Monitoring	44

4.4.	Confirmation of CD4+ Cell Count and Viral Load Levels Prior to Making a Change in Therapy	45

4.5.	Development of Opportunistic Infections, Bacterial Infections, or Malignancy	45

4.6.	Study Drug Toxicity and Grading	45

4.7.	Pregnancy and Breastfeeding	45

4.8.	HIV Transmission Counseling	46

4.9.	Coenrollable Studies	46

4.10.	Event Reporting	46

4.10.1.	Initiation and Discontinuation of Treatment in the DC Group	46

4.10.2.	Retroviral Rebound Syndrome	47

4.10.3.	Disease Progression Events	47

4.10.4.	Grade 4 Events, Deaths and Major Cardiovascular and Metabolic Complications	47

4.10.5.	Serious and Unexpected Adverse Experiences	47

5.	Evaluation	49

5.1.	Data Analysis	49

5.1.1.	Plans for Describing the DC and VS Strategies	49

5.1.2.	Plans for Addressing Primary and Secondary Objectives	50

5.2.	Data Monitoring	51

5.2.1.	Monitoring the Implementation of the Protocol by the DSMB and the Protocol Team	51

5.2.2.	Monitoring of Treatment Differences in Clinical Outcomes by the DSMB	51

6.	Procedures	53

6.1.	Reference Documents	53

6.2.	Data Collection and Monitoring	53

6.3.	Publications and Presentations	53

6.4.	Human Subjects	53

7.	Substudies	55

7.1.	Quality of Life and Healthcare Utilization Substudy (CPCRA 065A)	55

7.1.1.	Rationale	55

7.1.2.	Patient Selection	56

7.1.3.	Objectives	56

7.1.4.	Measurements	56

7.1.5.	Selected outcome measures	57

7.1.6.	Subsampling Plan and Data Analysis	59

7.2.	HIV Transmission Risk Behavior Substudy (CPCRA 065B)	61

7.2.1.	Rationale	61

7.2.2.	Patient selection	62

7.2.3.	Objectives	62

7.2.4.	Measurements	63

7.2.5.	Subsampling plan and statistical methods	64

7.3.	Body Composition Substudy (CPCRA 065C)	65

7.3.1.	Rationale	65

7.3.2.	Objectives	68

7.3.3.	Selected Outcome Measures	68

7.3.4.	Patient Selection	69

7.3.5.	Measurements	69

7.3.6.	Subsampling Plan and Statistical Analysis	70

Appendix A. Hypothetical CD4+ Cell Count Patterns Over Follow-up	A-1

Appendix B.	Sample Informed Consent	B-1

Appendix C.	Sample Informed Consent for the Storage of Specimens	C-1

Appendix D.	Sample Informed Consent for Women who Become Pregnant	D-1

Appendix E.	Time-and-Events Schedule	E-1

Appendix F.	SMART Protocol Team	F-1

References	R-1

# 1. Synopsis

**Rationale:**	Implementation of antiretroviral treatment guidelines, which emphasize maximal and durable suppression of viral load for the majority of individuals infected with HIV, has resulted in a substantial decline in morbidity and mortality. However, many asymptomatic patients are not at immediate risk of serious opportunistic diseases, the effectiveness of antiretroviral therapy wanes over time due to HIV drug resistance, and there are both short- and long-term toxicities of treatment. This motivates a comparison of two strategies, one which conserves treatments by deferring their use while the risk of opportunistic disease is low and one which aims for sustained virologic suppression irrespective of disease risk.

**Purpose:**	The purpose of this study is to compare the long-term clinical consequences of two strategies of antiretroviral (AR) management:

- the drug conservation (DC) strategy, a strategy aimed at conserving drugs through episodic use of antiretroviral treatment for the minimum time to maintain CD4+ cell count ≥ 250 cells/mm^3^

versus

- the viral suppression (VS) strategy, a strategy aimed at suppressing viral load as much as possible, immediately following randomization and throughout follow-up, irrespective of CD4+ cell count.

**Study**

**6,000 HIV-infected patients with CD4+ cell counts > 350 cells/mm^3^**

**Schematic:**

**Drug Conservation (DC) Group**

(n = 3,000)	(n=3,000)

[Stop or defer AR therapy until CD4+ cell count declines to below 250 cells/mm^3^; then treat to increase CD4+ cell counts

> 350 cells/mm³; then use episodic AR treatment based on CD4+ cell count.]

**Virologic Suppression (VS) Group**

(n = 3,000)	(n=3,000)

[Use AR treatment to maintain viral load as low as possible irrespective of CD4+ cell count by changing AR treatment when the viral load is not suppressed]

**Design:**	A large, long-term, randomized trial comparing two antiretroviral management strategies. Patients will be randomized in a 1:1 allocation ratio to either the DC or VS group. The primary endpoint is clinical disease progression or death. Follow-up will continue until 910 primary events are observed, a follow-up duration estimated to be 6-9 years. To achieve this required number of events, 6,000 patients will be enrolled and followed for an average of 7.5 years.

Patients will be enrolled over a 3.5-year period. During the first year, referred to as the “pilot phase”, the goal was to enroll 1,000 patients. This target was met, the treatment interventions were adhered to and appeared sufficiently distinct, and there were no safety concerns identified by an external review by the Data and Safety Monitoring Board (see section 5.2). Thus, additional sites are to be added to ensure that the enrollment goal of 6,000 patients is met.

Selected subsamples of patients enrolling into the SMART study will be followed with more intensive data collection for secondary outcomes relating to cost and health care utilization, quality of life, HIV transmission risk behaviors, and metabolic complications of treatment.

**Target**

**Population:**	This study will include HIV-infected patients with a CD4+ cell count > 350 cells/mm³ who are either taking or not taking AR therapy.

**Inclusion**

**Criteria:**	1.	Signed informed consent

2.	Evidence of HIV infection

3.	Age > 13 years

4. CD4+ cell count > 350 cells/mm^3^
5. Willingness to initiate, modify, or stop antiretroviral therapy, in accordance with the randomized assignment
6. If participating in sexual activity that could lead to pregnancy, willingness to use acceptable contraception methods.

**Exclusion**

**Criteria:**	1.	Current participation in the CPCRA FIRST, MDR-HIV or another study which is not consistent with one of the treatment groups in the SMART study (e.g., ESPRIT, SILCAAT)

2.	Current pregnancy or breastfeeding

**Procedures:**	Patients will be seen 1, 2, 4, 6, 8, 10 and 12 months following randomization and every 4 months thereafter for follow-up data collection visits. All available antiretroviral treatments, including immunomodulators, and resistance testing may be used by patients in both treatment groups.

**For patients assigned to the DC group:** Following randomization, none of the patients will take antiretroviral therapy. If they were taking therapy prior to randomization, they will stop. If they were not taking therapy prior to randomization, they will not begin. Use of antiretroviral treatments will be deferred until the CD4+ cell count falls below 250 cells/mm^3^. When AR therapy is initiated, the goal of therapy will be virologic suppression to maximize CD4+ cell count response. If the CD4+ cell count increases to > 350 cells/mm³ on two consecutive occasions at least two months apart, AR therapy should be discontinued.

**For patients assigned to the VS group:** Following randomization, all patients will take antiretroviral therapy. If they were taking therapy prior to randomization, they will continue it and modify it as necessary to suppress the viral load. If they were not taking therapy prior to randomization, they will initiate therapy aimed at suppressing the viral load. The antiretroviral treatment regimen should be changed whenever the viral load is not suppressed, in an attempt to achieve virologic control. In general, the goal is to maintain the virus below the level of detection or as low as possible throughout follow-up.

Both treatment groups will be managed as described above throughout the course of the trial.

**Data**

**Collection:	Most data collection will occur at regularly scheduled follow-up visits.** Selected events described in section 4.10 will be reported as they occur. More detailed measures of quality of life, healthcare utilization, metabolic consequences of treatment, and HIV transmission risk behavior will be collected in targeted substudies.

**Data**

**Monitoring:	Data will be monitored by the Division of AIDS Data and Safety Monitoring Board (DSMB).** During the first year of the study, the study was reviewed by the DSMB and by the Division of AIDS on two occasions, after 6 and 12 months. These evaluations focused on safety comparisons between the DC and VS groups, adherence to the planned interventions, and the achievement of enrollment targets. After completion of the “pilot phase”, the DSMB will continue to review the protocol at least once per year.

# 2. Background and Rationale

Two developments in the 1990s dramatically changed the treatment of HIV infection. Quantitation of HIV in plasma proved to be a potent predictor of disease progression among persons not on antiretroviral therapy, independent of immunological monitoring using the CD4+ cell count. Shortly thereafter, combination antiretroviral treatment regimens were introduced with the capacity to decrease the HIV below the limits of detection in the majority of adherent patients. The introduction of viral load monitoring and combination antiretroviral therapy was followed by unprecedented decreases in the morbidity and mortality of HIV infection.

Despite these successes, there are increasing concerns about the strategy of basing treatment decisions on viral load measurements and attempting complete viral suppression in all patients on treatment. Prolonged, complete suppression of plasma viral load does not eradicate HIV infection; discontinuation of therapy is followed by recurrent viremia. In clinic cohorts, approximately one-fourth of patients starting treatment either do not achieve virologic suppression or lose it in 2-3 years. Furthermore, patients with virological failure of an initial regimen have low rates of complete viral suppression with subsequent regimens. Finally, there is increasing evidence of significant metabolic and cardiovascular side effects with long-term antiretroviral therapy using the currently available agents.

Thus, there is considerable uncertainty in antiretroviral therapeutics. Viral load measurements have strong prognostic value in untreated HIV infection, but it is not clear that viral load monitoring is an ideal tool for decisions about changing therapy. Potent antiretroviral regimens are available, but may not achieve durable suppression in the majority of patients, and are complicated by short-term and long-term toxicity. These considerations led a group of experts in 1996 to recommend that “trials addressing the long-term effectiveness of therapies and strategies be undertaken.”^[1]^ More recently, the National Institutes of Health called for “study designs that evaluate strategic approaches to antiretroviral therapy, including when to initiate therapy, which agents to use, when to switch therapy, and how to maximize immune reconstitution.”^[2]^

Treatment guidelines in the late 1990s were based on the belief that viral suppression, as achieved by potent antiretroviral regimens, would prevent the development of HIV resistance and would result in better HIV outcomes. In the short-term, such guidelines, which have permeated clinical practice, have had the desired effect. However, the guidelines, as currently written, may not have the desired long-term effects; and that is the impetus for this investigation.

## 2.1. Treatment Guidelines

Treatment guidelines from three groups, the Department of Health and Human Services (DHHS)^[3]^, the International AIDS Society – USA Panel (IAS-USA),^[4]^ and the British HIV Association,^[5]^ have been developed, regularly updated, and widely disseminated.

All of the three guidelines emphasize the importance of virologic suppression and recommend the use of potent antiretroviral regimens. The main uncertainty in these guidelines is related to the question of when to start antiretroviral treatment. Over the past several years, the recommended criteria for initiation of antiretroviral therapy have been modified with recognition of the paucity of data supporting treatment in patients with less advanced HIV disease and with the appreciation of the risk of development of adverse events and the difficulty of adhering with treatment regimens. While initially the DHHS guidelines, for example, recommended initiation of therapy among patients with CD4<500 cells/mm^3^, by 2001, the guidelines were modified to utilize a CD4+ threshold of < 350 cells/mm^3^, and the 2003 guidelines are only definitive about starting therapy for those CD4+ ≤ 200 cells/mm^3^.^[6]^ Thus, for patients with CD4+ cell count ≥ 200 cells/mm^3^ current guidelines indicated that there remained substantial uncertainty about the benefits and risk of starting treatment. Another example of this uncertainty is the 2001 British Guidelines which recommend that antiretroviral treatment be considered for patients between 200 to 350 cells/mm^3^, rather than requiring treatment in this CD4+ stratum. These evolving thresholds for treatment reflect the paucity of data that directly address the issue of when is the optimal time to initiate antiretroviral treatment.

In general, once therapy is initiated, the various guidelines indicate that the goal should be achievement of maximal and durable suppression of viral load. However, there is some equivocation of this argument, with the suggestion that a rational sequencing of drugs should be considered with a goal of preserving future treatment options as long as possible.

With regard to changing therapy, the available guidelines stress that failure of virologic response or virologic failure are major indications for change in antiretroviral treatment. Additionally, declining CD4+ cell count or clinical deterioration are also criteria for changing treatment. In some circumstances the guidelines indicate that it may be rational to continue the current regimen if partial suppression is obtained and there are limited treatment options. In addition, the guidelines indicate that it is inadvisable to add or change a single drug; and that it is important to use at least two new drugs and preferably an entirely new regimen.

## 2.2. The Strategies for Management of Antiretroviral Therapy (SMART) Study

In order to obtain evidence to develop treatment recommendations, randomized trials are needed. Implementation of antiretroviral treatment guidelines, which emphasize maximal and durable suppression of viral load among the majority of individuals infected with HIV, have resulted in substantial declines in morbidity and mortality. However, many asymptomatic patients are not in immediate risk of serious opportunistic diseases, the effectiveness of antiretroviral therapy wanes over time due to HIV drug resistance, and there are both short- and long-term toxicities of treatment. Increasing evidence of long-term toxicities associated with antiretroviral treatment is the primary reason that the DHHS guidelines were changed in 2000 to take a more conservative approach to initiating treatment and express uncertainty about initiating treatment when the CD4+ cell count is > 350 cells/mm^3^.Error: Reference source not found This uncertainty logically extends to individuals already on treatment with elevated CD4+ cell counts, in particular if they began treatment when their CD4+ cell count was greater than 350 cells/mm^3^. Current guidelines do not address this issue. This motivates a comparison of two strategies, one of which conserves treatments by deferring their use while the risk of opportunistic disease is low based on CD4+ cell count and one based on sustained virologic suppression irrespective of disease risk.

This protocol describes a large trial to compare the long-term consequences of two very different strategies of antiretroviral management. Each strategy seeks to minimize the long-term complications of HIV disease, and each also seeks to preserve treatment options by minimizing the risk of drug resistance.

One strategy, the drug conservation (DC) strategy, aims to preserve as many treatment options as possible by drug deferral. Antiretroviral drugs are either stopped or not started until risk of opportunistic events begins to increase (i.e., the CD4+ cell count declines to < 250 cells/mm^3^). This is around the CD4+ level that current guidelines recommend treatment be started.Error: Reference source not found Treatment is then initiated, with a goal of suppressing viral load as much as possible in order to increase CD4+ cell counts to above 350 cells/mm^3^. Once counts have increased again to levels where the development of opportunistic diseases is unlikely, therapy should be discontinued.

The other treatment group (the control group), the viral suppression (VS) strategy, seeks to preserve and even enhance immune function by suppressing the viral load as much as possible and as long as possible throughout follow-up with available drugs, even when risk of opportunistic events is low (i.e., CD4+ cell counts > 250 cells/mm³). This strategy is currently standard-of-care in most places.

The names of the two groups, DC and VS, reflect the central tenet of each strategy. The comparison of these two strategies will provide critical information on the risks and benefits of a strategy of treatment deferral, and then episodic treatment, based on CD4+ cell count as compared with a strategy based on earlier and continuous treatment. As such, this study will address, in part, the broader questions of “when to start,” “when to change,” and “when to stop” therapy.

## 2.3. Rationale for a Drug Conservation (DC) Treatment Strategy

### 2.3.1. HIV Disease Progression Is Rare among Patients with High CD4+ Cell Counts

Opportunistic complications of HIV infection are rare when individuals have CD4+ cell counts greater than 200 cells/mm^3^.^[7]^ This appears to be the case even after a CD4+ cell count rebound from a low CD4+ nadir (see section 2.3.4).

In the EuroSIDA study, the incidence of AIDS-defining illnesses among patients whose latest CD4+ cell counts were > 200 cells/mm^3^ was 3.6 events per 100 patient-years of follow-up. For those with CD4+ cell counts 51-200 and ≤ 50 cells/mm^3^ the rates were 20.5 and 98.0 per 100 patient-years, respectively. Among patients prescribed highly active antiretroviral treatment (HAART), in each CD4+ stratum the rates were lower. Notably, for those with CD4+ cell counts > 200 cells/mm^3^, the rate was 1.4 per 100 patient-years of follow-up for those taking HAART.Error: Reference source not found

In the CPCRA NvR study (CPCRA 042), antiretroviral-naïve or nRTI- experienced patients with entry CD4+ cell counts < 200 cells/mm^3^ were randomized to different PIs as initial HAART therapy. CD4+ cell counts and HIV RNA levels (by RT-PCR) were collected during follow-up on a substudy of 610 patients (median baseline CD4+ cell count = 42 cells/mm^3^, median baseline HIV RNA level = 119,000 copies/mL). CD4+ cell counts increased to a median of 137 cells/mm^3^ after 12 months and to 154 cells/mm^3^ after 24 months. Over an average follow-up of 2.5 years, 172 patients (28.2%) developed a disease progression event or died. Only five (2.9%) patients who developed an event had proximal CD4+ cell counts of 250 cells/mm^3^ or higher (see figure 1 below). Among these five patients who developed an AIDS event or died, one died from end-stage renal disease and sepsis, one died from diabetes and sepsis, one developed lymphoma, one developed *Pneumocystis carinii* pneumonia (PCP), and one developed esophageal candidiasis.^Error: Reference source not found^

Figure 1	Distribution of Proximal CD4+ Cell Counts at Time of First Disease Progression Event

Thus, if therapeutic success is measured by the lack of clinical disease progression, the timing of therapy modification should be based more on risk of clinical progression than on virologic changes alone. A strategy of conserving drugs until the time that risk of opportunistic diseases begins to increase may lead to the use of fewer drugs over time, the availability of more potent regimens when the risk of disease is the highest, and fewer cumulative drug toxicities.

### 2.3.2. Proximal CD4+ Cell Count Is a Better Predictor of Disease Progression than Proximal HIV RNA Level

While among untreated individuals baseline plasma viral load appears to be a better long-term predictor of AIDS and death than baseline CD4+ cell count,^[8]^ for patients taking HAART, current CD4+ cell count is a better measure of short-term risk of disease progression than viral load. ^Error: Reference source not found,Error: Reference source not found^ Lundgren et al found that the additional prognostic value of recent viral load measurements was small after adjustment for CD4+ cell count and hemoglobin. Patients with a latest CD4+ cell count ≤ 50 cells/mm^3^ were at 9 fold risk of clinical progression compared to those with a CD4+ > 200 cells/mm^3^. Results from the CPCRA NvR study are consistent and the tables below illustrate the relative prognostic importance of CD4+ cell counts and HIV RNA levels on disease progression following the initiation of HAART.

Tables 1 and 2 below display the relationship of disease progression with follow-up CD4+ cell counts (table 1) and follow-up HIV RNA (table 2) in the NvR study^[9]^. Tertiles were defined by considering all CD4+ cell counts and HIV RNA levels measured during follow-up at 4-month intervals. For patients who experienced an event, the levels used are from the follow-up visit immediately preceding the event. The regression coefficient cited (Coeff.) is from a model that includes HIV RNA level and/or CD4+ cell count as a continuous variable.

Table 1

| **Relative Risk of Disease Progression or Death**  **According to Proximal CD4+ Cell Count Tertile** | | | | | | | | | | |
| --- | --- | --- | --- | --- | --- | --- | --- | --- | --- | --- |
|  |  | **Unadjusted** | | | | |  | **Adjusted for proximal HIV RNA** | | |
| **CD4+ Tertile**  **(cells/mm^3^)** |  | **Relative**  **Risk** | |  | | **95%**  **Confidence Interval** |  | **Relative Risk** |  | **95%**  **Confidence Interval** |
| 1 (< 80) |  | 12.8 | |  | | 6.2 to 26.6 |  | 7.0 |  | 3.3 to 15.0 |
| 2 (80-190) |  | 3.2 | |  | | 1.4 to 7.0 |  | 2.4 |  | 1.1 to 5.3 |
| 3 (>190) |  | 1.0 | |  | | (ref. group) |  | 1.0 |  | (ref. group) |
|  |  | Coeff* (SE): -.0131 (.0015) | | | | |  | Coeff* (SE): -.0104 (.0016) | | |
|  |  |  |  | |  | |  |  |  |  |
| * coefficient corresponding to proximal CD4+ (cells/mm^3^) | | | | | | | | | | |

Table 2

| **Relative Risk of Disease Progression or Death**  **According to Proximal HIV RNA Level Tertile** | | | | | | | | | | |
| --- | --- | --- | --- | --- | --- | --- | --- | --- | --- | --- |
|  |  | **Unadjusted** | | | | |  | **Adjusted for proximal CD4+** | | |
| **HIV RNA Tertile**  **(copies/mL)** |  | **Risk**  **Relative** | | |  | **95%**  **Confidence Interval** |  | **Relative Risk** |  | **95%**  **Confidence Interval** |
| 3 (> 50,000) |  | 6.6 | | |  | 3.8 to 11.4 |  | 2.2 |  | 1.2 to 4.1 |
| 2 (400-50,000) |  | 2.2 | | |  | 1.2 to 4.0 |  | 1.4 |  | 0.8 to 2.5 |
| 1 (< 400) |  | 1.0 | | |  | (ref. group) |  | 1.0 |  | (ref. group) |
|  |  | Coeff* (SE): .6366 (.0740) | | | | |  | Coeff* (SE): .3261 (.0841) | | |
|  |  |  |  |  | | |  |  |  |  |
| * coefficient corresponding to proximal HIV RNA (log 10) | | | | | | | | | | |

Follow-up CD4+ cell count was a more important determinant of risk of progression than follow-up HIV RNA level, both before and after adjustment for the alternate laboratory marker of HIV disease. A 7-fold difference in risk was evident between the highest and lowest tertiles of CD4+ cell count, while there was only a 2-fold difference in risk between the highest and lowest tertiles of HIV RNA level.

The important effect of CD4+ cell count on risk of disease is further evidenced in tables 3 and 4. These tables display the joint relationship of CD4+ cell count and HIV RNA level with risk of disease progression in this same NvR cohort.

Table 3

| **Relative Risk of Disease Progression or Death According to Proximal CD4+ Cell Count (Tertile) and Proximal HIV RNA Level (Median)** | | | | | | |
| --- | --- | --- | --- | --- | --- | --- |
| **Proximal CD4+** |  | **Proximal HIV RNA** |  | **Relative Risk** |  | **95% Confidence Interval** |
| Tertile 1 < 80 |  | ≥11,200 |  | 20.1 |  | 8.1 to 49.6 |
| < 80 |  | <11,200 |  | 5.6 |  | 1.9 to 16.0 |
| Tertile 2 80-190 |  | ≥11,200 |  | 3.9 |  | 1.3 to 11.3 |
| 80-190 |  | <11,200 |  | 3.8 |  | 1.4 to 10.3 |
| Tertile 3 > 190 |  | ≥11,200 |  | 2.5 |  | 0.6 to 10.4 |
| > 190 |  | <11,200 |  | 1.0 |  | (ref. group) |

Table 4

| **Relative Risk of Disease Progression or Death According to Proximal HIV RNA Level (Tertile) and Proximal CD4+ Cell Count (Median)** | | | | | | |
| --- | --- | --- | --- | --- | --- | --- |
| **Proximal HIV RNA** |  | **Proximal CD4+** |  | **Relative Risk** |  | **95% Confidence Interval** |
| Tertile 3 > 50,000 |  | < 110 |  | 17.3 |  | 7.5 to 40.0 |
| > 50,000 |  | ≥ 110 |  | 2.0 |  | 0.6 to 7.1 |
| Tertile 2 400-50,000 |  | < 110 |  | 8.3 |  | 3.4 to 20.1 |
| 400-50,000 |  | ≥ 110 |  | 1.9 |  | 0.7 to 5.3 |
| Tertile 1 < 400 |  | < 110 |  | 5.3 |  | 1.8 to 15.1 |
| < 400 |  | ≥ 110 |  | 1.0 |  | (ref. group) |

Both markers provide information on disease prognosis; however, the differences in relative risk estimates for those above and below the median CD4+ cell count for each HIV RNA level tertile (table 4) were more extreme than the difference in relative risk estimates above and below median HIV RNA levels for each CD4+ cell count tertile (table 3). Once CD4+ cell count was fixed, the additional information on short-term risk provided by viral load was not large, except in the lowest CD4+ cell count tertile. However, for each tertile of HIV RNA level, the additional information on short-term risk provided by the CD4+ cell count was substantial.

The results from an observational study of a large cohort of French HIV-infected patients who were placed on a protease inhibitor during between July 1996 and March 1997 also illustrate the importance of CD4+ cell count as a predictor of disease progression among patients on therapy.^[10]^ After 6 months on HAART, patients were classified as immunologic and virologic responders, immunologic responders only, virologic responders only, and non-responders (both virologically and immunologically). Risks of disease progression over the subsequent median follow-up of 18 months compared to complete responders were 1.55 (95% CI: 0.96 to 2.50), 1.98 (95% CI: 1.26 to 3.10), and 3.38 (95% CI: 2.28 to 5.02) for immunologic responders only, virologic responders only, and non-responders, respectively. In the absence of an immunologic response, risk of progression was significantly higher, irrespective of the virologic response.

### 2.3.3. Symptoms May Precede Immunologic Decline

Many patients who develop opportunistic diseases at higher CD4+ cell counts experience symptoms before onset. Two precursors of AIDS are oropharyngeal candidiasis and unexplained fever. These signs of immunologic decline are risk factors for the development of PCP and have been used for several years to guide the initiation of PCP prophylaxis^[11]^. In addition to CD4+ cell count, signs and symptoms can be monitored to help guide the initiation or reinitiation of therapy. If monitoring is done, the low risk of opportunistic disease at CD4+ cell counts > 250 cells/mm^3^ may be further reduced.

### 2.3.4. Immunologic Competency Can Be Recovered in Patients with CD4+ Cell Count Rebounds

Patients who have CD4+ cell count increases following initiation of HAART are at low risk of opportunistic infections, as evidenced by recent studies that have shown it is safe to discontinue opportunistic infection prophylaxis for disseminated *Mycobacterium avium* complex (MAC) and PCP in patients who have had CD4+ cell count rebounds.^[12],^^[13],^^[14],^^[15],^^[16],^^[17],^^[18]^ Immune restoration with potent antiretroviral therapy was the subject of a recent review, and the authors concluded that even partial immune restoration was sufficient to protect against most opportunistic diseases and that CD4+ cell count was an accurate predictor of risk.^[19]^

The CPCRA CR-MAC study (CPCRA 048) demonstrated that, in a group of patients with CD4+ cell counts < 50 cells/mm^3^ prior to HAART, there was broad-based protection against opportunistic events.Error: Reference source not found Among 520 patients enrolled in that study (prior median nadir CD4+ cell count = 23 cells/mm^3^) who had experienced rebound in their CD4+cell counts to >100 cells/mm^3^, none developed MAC or cytomegalovirus disease (CMV), only six developed PCP, and one developed toxoplasmosis, over an average period of follow-up of 1 year. The low rates of all opportunistic events in that study demonstrate that effective immunity can be restored with antiretroviral therapy, even in patients with nadir CD4+ cell counts below 50 cells/mm^3^.

### 2.3.5. Durability of Viral Suppression with HAART Is Similar Among Patients with CD4+ Cell Counts 200 to 349 cells/mm^3^ and CD4+ Cell Counts ≥ 350 cells/mm^3^

In the largest report to date, virologic response after starting therapy was similar for patients with baseline CD4+ cell counts 200 to 349 cells/mm^3^ compared to patients with CD4+cell counts ≥ 350 cells/mm^3^.^[20]^ Data from three European cohorts were combined, and the response of 2,742 patients after starting HAART therapy was evaluated. The relative hazard of a viral load < 500 copies/mL was 1.07 for those with baseline CD4+ cell counts 200 to 349 cells/mm^3^ versus those with CD4+ cell counts ≥ 350 cells/mm^3^ (p=0.43). Those with CD4+ cell counts < 200 cells/mm^3^ were significantly less likely to achieve viral loads < 500 copies/mL compared to those with CD4+ cell counts ≥ 350 cells/mm^3^ (relative hazard=0.75; p<0.0001). The magnitude of the differences between the three CD4+ cell count groups was not appreciably altered after adjustment for baseline viral load, age, gender, exposure category, previous AIDS, number of drugs prescribed, or calendar year of starting therapy. These data suggest that virologic response may not be compromised by deferring therapy until the CD4+ cell count declines to < 250 cells/mm^3^.

### 2.3.6. Durability of Viral Suppression with Currently Available HAART Is Limited

It is difficult to achieve and maintain viral suppression with available agents for a long period of time. Therefore, a long-term strategy aimed at enhancing immune function and minimizing drug resistance by means of viral suppression may not be as successful as other approaches. While some studies indicate that 70-80% of patients achieve a viral load ≤ 500 copies/mL, ^Error: Reference source not found,^^[21]^ other studies of responses to first-line HAART regimens in clinic populations reveal that within 24-48 weeks of initiating therapy, 50-60% of individuals will have evidence of virologic failure as defined by current treatment guidelines.^[22]^ Even in trials, an average of only 65% of patients had documented viral loads < 400 copies/mL after 6 months on their initial regimens, although in some trials those who discontinued randomized treatment were considered failures.^[23]^ Responses to second-and third-line regimens are even poorer.^[24],^^[25],^^[26]^ For example, in the CPCRA GART study (CPCRA 046), among patients failing their first PI-containing regimens and placed on second-line regimens based on results of genotypic resistance testing and expert opinion, 58% had HIV RNA levels > 500 copies/mL after 12 weeks. This percentage was 75% for patients who were placed on salvage regimens following multiple PI-containing regimens. Corresponding percentages for patients assigned to the control group, who did not have their salvage regimens determined by genotypic testing and expert advice, were 73% and 82% for the second-line and salvage regimens, respectively. In the VIRADAPT study, a similarly large percentage of patients (68%), whose salvage regimens were determined in part by results of genotypic resistance testing, had viral loads above assay detection limits (200 copies/mL) 6 months following a treatment change.Error: Reference source not found Two other larger observational studies have confirmed these findings.^[27],^^[28]^ Thus, even with new tools for decision-making concerning salvage regimens, virologic response is poor.

### 2.3.7. Risk of Resistance Increases with Drug Pressure and May Even Occur in the Presence of Viral Suppression

If antiretroviral therapy is discontinued in a patient, the selective pressure for the development of resistance strains associated with the treatment regimen will be removed. Similarly, in patients for whom initiation of therapy is deferred, risk of resistance due to selective pressure will also be deferred. Thus, a strategy that conserves antiretroviral drugs until the risk of opportunistic diseases begins to increase may result in a shorter duration of time during which there is a risk of developing resistance.

The risk of resistance increases the longer one is on therapy. Since the ability to maintain virologic suppression diminishes with each successive HAART regimen,Error: Reference source not found^,Error: Reference source not found,Error: Reference source not found^ changes in therapy are likely to lead to more drug resistance. The accumulated mutations frequently lead to cross-resistance among agents, with the end result being the development of multidrug resistance.

Even in the setting of persistent plasma viremia less than 50 copies/mL while on HAART, low-level virus replication may occur. Initial studies suggested that replication-competent virus arising from these latent reservoirs of viral replication did not contain new drug resistance mutations.^[29],^^[30]^ However, recent work has suggested that, over time, new resistance mutations can be detected in replication-competent virus recovered from latently infected peripheral blood mononuclear cells (PBMCs).^[31]^ Thus, even while a patient is using drugs that appear very effective, risk of resistance is present.

### 2.3.8. Long-Term Use of Antiretroviral Drugs is Associated with Unintended Side Effects

The use of a multitude of antiretroviral agents has the potential risk of development of adverse events, with some having substantial morbidity and long-term impact. Most adverse events are mild to moderate in nature, and patients may be able to tolerate continued therapy. However, other adverse events have a substantial impact on quality of life and require the use of other medications to mitigate them, e.g., the need for continued use of antidiarrheal agents in conjunction with the use of nelfinavir. More severe treatment limiting side effects have resulted in the need to permanently discontinue some medications, e.g., the development of recurrent renal calculi in association with indinavir^[32]^ or peripheral neuropathy in association with certain nucleosides.^[33]^ Another example of a severe and potentially life-threatening adverse event is the reported occurrence of pancreatitis and some deaths in association with the use of didanosine and/or hydroxyurea.^[34]^

Metabolic complications and changes in body habitus have also been reported among patients on antiretroviral therapy.^[35]^ These complications have resulted in distressing psychological effects, have had a major adverse impact on quality of life, and, in some cases, have necessitated switching medications. Anecdotal reports suggest a possible association of the use of potent antiretroviral therapy with other complications, e.g., cardiovascular conditions,^[36]^ osteonecrosis^[37]^, lactic acidosis,^[38]^ and mitochondrial toxicity syndromes.^[39]^ A large prospectively planned observational study recently reported that the risk of myocardial infarction increased by 26% with each year of exposure to combination antiretroviral therapy.^[40]^

The use of several antiretroviral agents, the cumulative number of agents used, and the duration of antiretroviral therapy may all contribute to the occurrence of adverse effects experienced by patients. Thus, a strategy that treats when disease risk begins to increase may be associated with fewer adverse events.

### 2.3.9. Adherence to Antiretroviral Therapy Is Difficult

Complete viral suppression appears to require a very high level of adherence; in one study using electronic monitoring of adherence with protease inhibitor-based therapy, virologic failure (viral load > 400 copies/mL) occurred among 22% of patients who had more than 95% adherence, 61% of patients with adherence between 80 and 95%, and in 80% of patients with less than 80% of adherence.^[41]^ A very high degree of adherence requires an extraordinary commitment by patients, a level of commitment that may be extremely difficult to sustain for life. Adherence with self-administered tuberculosis treatment, for example, declines markedly after 6 months of therapy.^[42]^ A decline is likely also the case for adherence to antiretroviral therapy, as evidenced from a recent CPCRA investigation of 633 patients in protocols studying first- and second-line antiretroviral regimens. In that study there was a significant change in adherence over time. The percentage of patients reporting 100% adherence was 70% at 1 month, 63% at 4 months and 58% at 8 months (p < 0.05).^[43]^

Side effects are a commonly reported reason for nonadherence to treatment.^[44]^ The occurrence of bothersome short-term side effects and worrisome long-term metabolic side effects and the need for very high-level adherence are leading to patient “burn-out.” An advantage of deferred therapy may be improved motivation and better adherence with therapy at a time when the risk of disease progression begins to increase.

## 2.4. Rationale for a Virologic Suppression (VS) Strategy

### 2.4.1. Baseline HIV RNA Level Predicts Progression to AIDS in Untreated Participants

Plasma HIV RNA levels indicate the magnitude of HIV replication. If left unchecked, replication of HIV leads to progressive immune system damage and to the development of

AIDS-defining illnesses. It has been clearly established that, among untreated individuals, baseline plasma viral load levels are an important determinant of risk of disease.Error: Reference source not found^,^^[45]^ While this may be less relevant for assessing risk of disease among treated individuals, these data nevertheless formed the basis for early treatment guidelines. The relationship of viral load with risk of disease in untreated individuals is illustrated in Table 5 with data abstracted from the DHHS report on principles of HIV therapy.^[46]^ Results are shown for two cohorts of homosexual men, stratified by baseline CD4+ cell count, that correspond to the inclusion criterion for the SMART study. This investigation was conducted prior to the availability of HAART, and only 41% of the participants received antiretroviral therapy at any time during follow-up. These data indicate that over a 6-year follow-up period, the approximate follow-up period of the SMART study, there is a high risk of progression that increases with HIV RNA level in each CD4+ cell count-defined stratum. These data also indicate, and more detailed analyses have confirmed, that baseline HIV RNA level is a more important determinant of long-term risk than baseline CD4+ cell count. The two markers together provide an even better assessment of risk. ^Error: Reference source not found^ Since HIV RNA level is an excellent predictor of rate of immunologic decline, as measured by the CD4+ cell count, and of clinical progression, it should be the primary focus of antiretroviral treatment management.

Table 5

| **Risk for Progression to AIDS-Defining Illness after 6 Years in a Cohort of Men, the Majority of Whom Did Not Receive Treatment, by Baseline CD4+ Cell Count and Viral Load** | | | |
| --- | --- | --- | --- |
| **CD4+ Cell Count**  **(cells/mm^3^)** | **Viral Load**  **(copies/mL, by bDNA)** | **No. Men** | **6-Year Risk (%)** |
| 351-500 | ≤ 500 | N/A | N/A |
|  | 501-3000 | 47 | 22.1 |
|  | 3001-10,000 | 105 | 39.8 |
|  | 10,001-30,000 | 121 | 57.2 |
|  | >30,000 | 121 | 77.7 |
| >500 | ≤500 | 110 | 5.0 |
|  | 501-3000 | 180 | 14.9 |
|  | 3001-10,000 | 237 | 25.9 |
|  | 10,001-30,000 | 202 | 47.7 |
|  | >30,000 | 141 | 66.8 |

### 2.4.2. Regimens that Provide Maximal Suppression Are Associated with a Lower Risk of Resistance

The best approach to obtain a durable virologic response is to use a combination antiretroviral regimen that suppresses replication to the maximal extent possible, currently defined as a viral burden below 50 copies/mL on standard assays.^[47]^ While there are differences among individual drugs in the rate at which resistance will occur, it is assumed that all antiretroviral drugs will lose effectiveness eventually if HIV is allowed to replicate in the presence of drug.^[48]^ Common mutations in HIV-1 genes that confer drug resistance have recently been summarized with guidelines for drug resistance testing.^[49]^ Persistent viral replication in the presence of antiretroviral drug treatment allows pre-existing mutations, as well as new ones, to occur and grow.^[50],^^[51]^ This selective pressure permits replication of mutations that have a growth advantage in the presence of the chosen medications. It has been noted that, once mutations occur, medications will be less successful from that point forward, although experimental interventions are underway to better assess what options there might be to minimize the future impact of resistance in those situations.^[52]^

The goal of achieving a durable response increases in importance due to the nature of cross- resistance with currently available medications. Durable suppression would be less necessary if there were sufficient numbers of HIV treatments that did not lose efficacy due to cross-resistance. However, if resistance occurs to one drug, there is some risk of decreased potency from other drugs that have never been used.^[53]^ Further, cross-resistance has been noted to be progressive over time. Initial mutations can sometimes be specific to one agent and not result in cross-resistance or be associated with only one of the drugs in a combination regimen.^[54]^ However, as HIV replicates in the presence of the agent, broader cross-resistance can occur, with increasing numbers of mutations. These additional mutations not only allow for more resistance to the initial drug but may also result in more cross-resistance to other drugs in the same class. It is these principles of cross-resistance that increase the necessity to suppress HIV replication and to move quickly to another, more suppressive regimen in cases of viral escape.

### 2.4.3. Changing Regimens at Lower Viral Loads Is More Likely to Result in Viral Suppression

As expected, patients with low viral loads at the time of treatment initiation or a regimen change are easier to suppress on a new regimen than those with higher viral loads. The largest cohort study of virologic response following initial therapy indicates that patients with viral loads ≥ 100,000 copies/mL are 35% less likely to achieve viral loads < 500 copies/mL after 32 weeks than those with lower viral loads.Error: Reference source not found Other studies have shown that when a PI-based regimen is changed due to lack of virologic suppression, the next potent regimen is more likely to result in complete suppression if it is initiated at a lower viral load.Error: Reference source not found^,Error: Reference source not found,Error: Reference source not found^ A small retrospective study examined 29 individuals with virologic failure, defined as detectable viral load after 16 weeks of a regimen containing indinavir or nelfinavir, who were switched to ritonavir plus saquinavir.^[55]^ Of the patients who had previously received nelfinavir, three responded to ritonavir plus saquinavir for an average of 27 weeks**.** There was a trend toward the responders being switched earlier (i.e., after less time on the failing regimen), although this trend was not statistically significant.

An open-label study investigated the use of ritonavir plus saquinavir with d4T plus 3TC in patients who were failing a regimen containing nelfinavir and who had participated in other trials of nelfinavir.^[56],^^[57]^ One of the predictive factors for nonresponse was higher viral load at the time of switch.

Another study (ACTG 372B) examined a group of individuals with HIV RNA levels above 500 copies/mL on an indinavir-containing regimen (mostly rollovers from another ACTG trial of indinavir – ACTG 320). This study compared abacavir versus approved nRTIs and nelfinavir versus placebo, in combination with a background of efavirenz and adefovir. All of the patients were NNRTI-naïve. Ninety-two patients were enrolled and followed for 16 weeks. Patients had a average CD4+ cell count and HIV RNA level at baseline of 196 cells/mm^3^ and 39,102 copies/mL, respectively; and results were stratified by baseline HIV RNA level of > or ≤ 15,000 copies/mL. Overall, 81% of individuals with HIV RNA > 15,000 copies/mL had viral load levels of > 500 copies/mL at 16 weeks; the corresponding result for those with HIV RNA ≤ 15,000 copies/mL was 43%.^[58]^

### 2.4.4. Treatment Interruptions Result in Increases in Viral Load and CD4+ Cell Count Declines

In studies of patients failing virologically who have undergone subsequent interruption of antiretroviral therapy, viral load levels have risen rapidly.^[59],^^[60],^^[61],^^[62],^^[63]^ This increase in viral load is associated with a sharp decline in the CD4+ cell count, which may take many months to recover after a new antiviral regimen is initiated. In addition, there are reports of symptoms consistent with seroconversion in those undergoing an interruption. Thus, there are risks in stopping HIV treatment after it has been initiated. The results of the CPCRA MDR-HIV study (CPCRA 064) highlight those risks. ^Error: Reference source not found^ It was stopped early based on an interim analysis that indicated that the 4-month treatment interruption was associated with an increased risk of AIDS or death and poorer immunological recovery compared to the control group. However, the results of that study have little relevance to the SMART study because of design and population differences. For example, in the SMART study, treatment is reinitiated for patients in the DC group when the CD4+ cell count declines below 250 cells/mm^3^. This may occur at any time, i.e. after one month or after several months or years. In the MDR-HIV study, by design, all patients assigned to the STI group were to interrupt antiretroviral therapy for a set period of time, i.e. 4 months, and antiretroviral therapy was reinitiated before 4 months only if the patient's CD4+ cell declined by 50% from baseline or if they experienced an opportunistic event. Also, the average CD4+ cell count at baseline in the MDR-HIV study was 180 cells/mm^3^; while in the SMART study it must be greater than 350 cells/mm^3^ at entry. Furthermore, patients in the MDR-HIV study had few treatment options. All had viral loads at entry > 5,000 copies/mL and multidrug resistant virus. Of most importance, for patients in the DC group in the SMART study, treatment is to be reinitiated when the CD4+ cell count declines to below 250 cells/mm^3^, a CD4+ cell count associated with low risk of opportunistic disease.

The relative safety of interruption at higher versus lower CD4+ cell counts was recently examined in large observational study.^[64]^ In that study interruptions were associated with an increased risk of disease when the CD4+ cell count at interruption was < 200 cells/mm^3^ but not when the CD4+ cell count was higher. Taken together, these data suggest that a better strategy for patients with low CD4+ cell count is to switch to a new regimen that is reasonably anticipated to be successful in reestablishing viral suppression.

Fewer studies have been carried out among patients with suppressed viral loads who stop therapy. One small study suggests that viral loads may return to pre-treatment levels even after several years of treatment.^[65]^ Another small study in patients with a median CD4+ cell count of 554 cell/mm^3^ at the time of interruption reported a decline in CD4+ cell count of 16 cells/mm^3^ per month (IQR: 6 to 34 cells per month) following interruption.^[66]^ These authors concluded that discontinuing therapy in patients whose viral loads are suppressed was safe if CD4+ cell counts were properly monitored.

Case reports indicate that interruption of therapy may result in a “retroviral rebound” syndrome, an acute illness that resembles primary HIV syndrome.^[67],^^[68]^ Another potential risk of treatment interruptions is the expansion of pools of infected memory T cells. Douek et al found in a study of 12 patients that following treatment interruption the frequency of HIV viral DNA in the HIV-specific pool of memory CD4+ T cells increased more so than in memory CD4+ T cells of other specificities.^[69]^

Thus, treatment interruptions may be risky both among patients failing on their HAART regimens, and among patients with adequate viral load suppression. For that reason, it is important that strategies like that employed for the DC group be evaluated in randomized trials.

### 2.4.5. Viral Suppression Is More Durable among Patients Who Start Therapy at Higher CD4+ Cell Counts

While there is only limited evidence from randomized trials on the optimal time to initiate therapy, observational data indicate that better suppression of viral load and greater CD4+ cell count increases are observed when therapy is initiated at higher CD4+ cell counts.Error: Reference source not found^,^^[70],^^[71]^ A large observational study of initial therapy found in univariate analyses that patients with CD4+ cell counts ≤ 200 cells/mm^3^ were 25% (p=0.01) less likely to achieve viral loads < 500 copies/mL after 32 weeks than patients with higher CD4+ cell counts.Error: Reference source not found In multivaritate analyses, patients with lower CD4+ counts were 19% less likely to achieve suppression (p=0.14) Another study of initial HAART therapy, involving at least three antiretroviral drugs including a PI, compared virologic response by baseline CD4+ cell count.^[72]^ There was a significant difference according to baseline CD4+ cell count, with 43% of those who had CD4+ cell counts greater than 350 cells/mm^3^ achieving durable viral loads < 400 copies/mL compared with 34% of those with CD4+ cell counts 200-350 cells/mm^3^, and 28% of those with baseline CD4+ cell counts < 200 cells/mm^3^. These results were corroborated by a second report that described the durability of viral suppression among patients taking three- drug regimens including an NNRTI. In that study, baseline CD4+ cell count predicted durability of suppression and baseline viral load did not.^[73]^

Immune reconstitution will be optimized when HIV replication is maximally suppressed. Partially suppressive regimens result in less substantial increases in CD4+ cell count.^[74],^^[75]^ In addition, other studies indicate that there appear to be potential risks in letting the CD4+ cell count drop to below 350 cells/mm^3^ in terms of reestablishing viral suppression.^[76]^

### 2.4.6. Many Antiretroviral Drugs Are Available

By the summer of 2001, there were 15 approved antiretroviral medications in the US. While there are some overlapping toxicities to these drugs, there are also sufficient differences in these compounds to allow consideration of substitutions of one treatment for another in the case of toxicity. These substitutions should be equally successful in maintaining suppression if there is no underlying resistance to the new medication. Furthermore, there are a number of new compounds in development to treat HIV. Among them are several novel compounds that could be beneficial for treatment-experienced patients.

### 2.4.7. Nadir of CD4+ Cell Count Predicts Risk of Opportunistic Diseases

Studies have explored the extent of immune reconstitution associated with antiretroviral therapy. Recent cohort studies have also explored the significance of the CD4+ cell count nadir and the long-term implications of this nadir on risk of opportunistic diseases, even after CD4+ cell count reconstitution.Error: Reference source not found These cohort studies have reported a prolonged impact of the nadir on risk of disease, suggesting that, while there is significant immune reconstitution with antiretroviral therapy, it may not be complete, justifying treatment before there is a significant CD4+ cell count decline.^[77]^ However, it remains undefined when in the course of HIV infection there is irreversible damage to the immune system.

Other support for early therapy comes from observational data indicating that CD4+ cell count nadir < 150 cells/mm^3^ is associated with increased risk of progression among patients with current CD4+ cell counts of at least 200 cells/mm^3^.^[78]^ This association appears to be independent of antiretroviral treatment, age, prior AIDS, and CD4+ cell count at the beginning of follow-up.

## 2.5. Benefits and Risks of Each Strategy

The SMART study compares a strategy of drug conservation while the risk of opportunistic diseases is low with a strategy of virologic suppression. The goal of each treatment approach is to prolong disease-free survival and to maintain a good quality of life for HIV-infected patients. There are clear benefits and risks of each strategy; and, based on available data, the optimal approach is not clear. The only way to reliably establish which strategy is more effective is to conduct a randomized trial. Risks and benefits of each strategy are summarized below.

### 2.5.1. Benefits and Risks of the Drug Conservation (DC) Strategy

For prolonging disease-free survival, potential benefits of a drug-conservation strategy, for which a substantial amount of follow-up time is spent off antiretroviral drugs, compared with one based on virologic suppression are:

- Potent drugs available when risk of disease increases
- Lower risk of drug resistance due to shorter duration on therapy
- Fewer side effects
- Better adherence

Potential risks of this strategy include:

- Greater risk of drug resistance at the time therapy is stopped and when it is reinitiated
- Greater risk of irreversible damage to immune system
- Increased risk of transmission

### 2.5.2. Benefits and Risks of the Virologic Suppression (VS) Strategy

For prolonging disease-free survival, potential benefits of a strategy based on virologic suppression compared with one that conserves drugs until the risk of disease begins to increase are:

- A more durable virologic response
- Lower risk of drug resistance if virologically suppressed
- Lower risk of irreversible CD4+ cell count depletion
- Lower risk of transmission

Potential risks of the VS strategy are:

- More side effects
- Poorer adherence to drug regimens due to complicated regimens and patient “burnout”
- Fewer drug options when risk of disease is high
- Resistance to more drugs upon virologic failure

## 2.6. Rationale for Selected Study Design

The SMART study has been developed to address a clinically relevant management question for which there is substantial uncertainty. The study design takes into account various clinical needs, management practices, and design issues. There are, nevertheless, some developments that could either obviate the need for a trial like the SMART study or could compromise the study question. For example, if a new treatment became available that could eradicate the HIV virus, there would be no need for a trial like the SMART study. Alternatively, if a treatment became available that was very easy to take, was very effective in suppressing viral load for many years, was associated with a low risk of resistance, and had minimal side effects, and if both treatment groups in the SMART study began taking the treatment, the trial question could be compromised, depending on the maturity of the SMART study when the drug became available. These are not likely possibilities over the next decade. Thus, the question addressed by the SMART study will remain relevant. The introduction of new and hopefully better treatment options will be incorporated into the SMART study and these treatments may further reduce morbidity and mortality from HIV. Further reduction in rates of morbidity and mortality may require the trial to be extended to obtain the required number of events, but such advances in treatment will not compromise the study question.

Key elements in the study design are summarized below prior to the presentation of the design of the study.

### 2.6.1. The Need for Two Distinct and Sufficiently Different Treatment Strategies

The two treatment strategies need to be sufficiently different in their definition so that a difference in clinical outcomes between them is plausible. Thus, the two strategies were selected to result in substantially different treatment groups.

The DC strategy uses CD4+ cell count as the primary clinical marker for guiding therapy initiation or changes. Since risk of opportunistic diseases is very low for patients with CD4+ cell counts ≥ 250 cells/mm^3^, this CD4+ cell count was chosen as the threshold to initiate or re-initiate therapy in the DC group. This CD4+ cell count threshold, together with the minimum CD4+ cell count required for entry (350 cells/mm³), will ensure a different form of antiretroviral management from the VS group for much of the follow-up period.

The VS group uses viral load as the primary laboratory marker for guiding therapy. Therapy should be initiated or changed if viral load is not suppressed, irrespective of the CD4+ cell count. For the VS group, the goal of therapy throughout follow-up should be virologic suppression in order to maximize the immunologic response and to reduce the risk of drug resistance.

These two very different strategies of management should result in treatment groups with substantially different CD4+ cell counts and HIV RNA levels during the first several years of follow-up, a time, as a consequence of the entry criteria, during which risk of opportunistic disease will be very low. CD4+ cell count averages are initially expected to be lower in the DC than the VS group, and HIV RNA levels are expected to be higher. Later in the follow-up period, when risk of opportunistic disease is greater, these disease markers are expected to converge and possibly cross (see appendix A). Figure A.1 in appendix A illustrates different hypothetical trajectories for CD4+ cell counts during the conduct of the study.

### 2.6.2. The Need for Primary and Secondary Clinical Endpoints

The ultimate goal of the treatment of HIV disease is to prevent clinical morbidity and mortality. Thus, the primary and secondary endpoints of this study are clinical outcomes rather than surrogate markers.

The primary endpoint of the SMART study is HIV disease progression or death because the principal goal is to determine which of these two strategies is superior in maintaining adequately elevated CD4+ cell counts in the long-term, thereby delaying HIV disease progression. It is recognized that these two very different strategic approaches to therapy could result in a different pattern and incidence of major toxicities. It is also recognized that some of the major toxicities may have substantial associated morbidity. For this reason, toxicities are major secondary endpoints. All major toxicities will be collected through the CPCRA adverse event collection system and the metabolic/cardiovascular complications through the CPCRA supplemental event reporting system. Therefore, throughout the study clinical disease progression as well as toxicity data will be collected, which will allow for the careful weighing of the benefits and risks of each treatment strategy at interim and final analyses.

### 2.6.3. The Need for Long-Term Follow-up

In order to optimize HIV therapy, information concerning the long-term risks and benefits of different treatment strategies is needed. While much is known about the short-term effects of different antiretroviral treatments, little is known about their long-term effects, which may or may not be consistent with the results of short-term studies. Thus, the duration of follow-up of the proposed study is long to arrive at the needed answers.

The planned minimum follow-up of the SMART study is 6 years; the average is expected to be 7.5 years. This period of follow-up and the planned sample size are based on a number of assumptions outlined in Section 3.5 (Sample Size and Statistical Considerations). During the conduct of the study, information on relevant event rates from other studies will be evaluated and the event rate in the SMART study will also be closely monitored by the Division of AIDS Data Safety and Monitoring Board (DSMB). This monitoring may necessitate change in the duration of follow-up. For example, longer follow-up may be required if the planned number of events is not obtained as projected by the design.

### 2.6.4. The Need for a Trial to Detect Moderate Differences in Clinical Outcomes between the Treatment Strategies

The SMART study requires a large sample size to reliably assess the effects of the two different antiretroviral strategies on clinical outcomes, both the opportunistic effects that antiretroviral treatment aims to prevent and those that are unintended, deleterious effects of the treatment used. The incidence of opportunistic diseases over 6 to 9 years is expected to be low among patients with entry CD4+ cell counts > 350 cells/mm^3^. Also, unlike the dramatic declines in morbidity and mortality that were observed among advanced patients when protease inhibitors were introduced,^[79],^^[80]^ large treatment differences for major clinical outcomes are not expected when comparing different strategic approaches for using antiretroviral therapy over a long time period. Thus, even though two treatment strategies have been defined that differ substantially in their approach to management of patients with HIV, a difference of greater than 15-25% in progression to AIDS or death over the planned follow-up appears unlikely. Nevertheless, a difference of 15-25%, if observed, would have a substantial influence on the management of patients and would provide the clinical evidence that is notably absent from the current guidelines. Thus, the SMART study is designed to detect a 17% difference in clinical disease progression between treatment groups.

### 2.6.5. The Need for a Study with Broad Eligibility Criteria

The trial is broadly inclusive, with no restrictions on types of antiretroviral or concomitant treatments, to ensure rapid enrollment of a large number of patients, to ensure acceptance by patients and clinicians, and to ensure broad generalizability of the findings.

Essentially all HIV-infected individuals with CD4+ cell counts > 350 cells/mm^3^ are eligible. A CD4+ cell count of greater than 350 cells/mm^3^ for inclusion was specified to ensure an adequate time of treatment deferral in the DC group prior to reaching the CD4+ cell count threshold of 250 cells/mm³, below which initiation/change in therapy is recommended.

By design there are no exclusion criteria based on antiretroviral treatment history. The SMART study includes those who are antiretroviral-naïve, those previously on therapy and those currently on therapy. For both naïve and treatment-experienced patients the optimal approach to the conservation of treatment options until the time when risk of clinical disease begins to increase is an important management issue. For those who are antiretroviral naïve, the two SMART study treatment strategies are relevant to the “when to start therapy” question. In both naïve and experienced patients, there are potential risks and benefits to allowing ongoing viral replication to be maintained until the CD4+ cell count declines to < 250 cells/mm^3^, as planned in the DC group. For both treatment-naïve and -experienced patients, there are also potential risks and benefits of suppressing the virus irrespective of CD4+ cell count, as planned in the VS group. Thus, the strategic approaches to be studied are currently applicable to all HIV-infected patients.

### 2.6.6. The Need for Simple, Focused Data Collection and for Substudies to Aid in Understanding Treatment Differences

The primary focus of the proposed study is on the long-term clinical consequences of therapy. Many short-term studies on the virologic efficacy and safety of specific treatments have already been conducted, and during the course of the SMART study many more will be. These studies will provide useful information on the virologic and immunologic efficacy of different regimens; however, even collectively, they will not provide information on the long-term clinical consequences of treatment strategies as planned in the SMART study. In order to maintain this focus, data collection is fairly limited in the main study, and primarily involves 1) clinical outcomes to establish the risk/benefit profile of the two treatment strategies; and 2) intermediate measurements that will be used to aid the understanding and interpretation of the difference in clinical outcomes, or lack thereof, which is observed. The data collection plan for the SMART study is detailed in section 3.7 and the SMART *Protocol Manual of Operations.* A rationale for the stated plan follows.

Baseline data collection is aimed at documenting stage of HIV disease, co-infection with hepatitis B and C, treatment history, nadir and baseline CD4+ cell count, HIV RNA level, and genotypic resistance profile. These data along with patient demographics and information on possible HIV transmission categories will be used to characterize the study cohort, to form subgroups for making treatment comparisons, and to study predictors of response to the assigned treatment. Other measurements based on the medical history, resting electrocardiogram (ECG), body cell mass by bioelectrical impedance analysis (BIA), blood lipids, and self-reported body appearance and adherence are recorded to establish a baseline for each patient for measuring change. For a subsample of consenting patients, blood samples are stored for future CPCRA-approved research. It is expected that these valuable samples will be used for conducting nested case-control studies in which samples are analyzed for patients who experience a specific event (e.g., an AIDS event) and for controls.

During the first year, data collection is more intense than during subsequent years so that immunologic and virologic changes following randomization can be carefully monitored and so that any safety issues related to the DC strategy are quickly identified. CD4+ cell count and HIV RNA levels at each follow-up visit will be used to characterize the rate of change in these markers following discontinuation of antiretroviral treatment in the DC group. These data will also be used to monitor adherence to the VS strategy.

Currently, minimal information is available to characterize the CD4+ cell count decline following discontinuation. Based on one small study of patients who achieved virologic suppression

< 500 copies/mL and discontinued therapy, it is expected that the decline following treatment interruption Error: Reference source not found will be much more rapid (16 cells/mm^3^ per month) than declines observed in untreated individuals following seroconversion.^[81]^ However, it is unclear whether the decline to nadir is more rapid than the subsequent decline and whether the decline varies by degree of virologic suppression, genotypic profile and treatment history at baseline. In the study by Tebas et al the decline in CD4+ cell count was not related to nadir CD4+ cell count but was related to the rate at which CD4+ cell count increased when antiretroviral therapy was initiated.Error: Reference source not found During the first year of the study, predictors of CD4+ cell count decline, HIV RNA level increase and time to re-initiation of antiretroviral therapy will be closely monitored by the Data and Safety Monitoring Board. Since the rate of reversion of drug resistant virus to wild type among patients in the DC group with baseline drug resistance is also of interest because it may predict the patient’s response to antiretroviral therapy when it is re-initiated, specimens for future HIV resistance testing will be stored at each follow-up visit for a subsample of patients.

Following the first year, patients will be seen every 4 months for data collection purposes. These regular data collection visits will be used to monitor CD4+ cell count and HIV RNA levels, adherence to the assigned strategy and to antiretroviral therapy for those prescribed it, to record changes in antiretroviral treatment, and to collect blood samples for storage. Annual data collection will be more comprehensive than at 4 and 8 month visits. Annual visits will be used to monitor changes in the resting ECG, self-reported body appearance, body cell mass, and blood lipids. Resting ECGs are being recorded and centrally analyzed primarily as a means of identifying patients with unrecognized myocardial infarction and major electrophysiological dysfunction that can only be diagnosed from the ECG, such as major arrhythmias, which increase in incidence from HIV infection, treatment or opportunistic infection.^[82],^^[83]^ Based on previous studies, the incidence of myocardial infarction will increase substantially by also considering “silent myocardial infarctions”.^[84],^^[85]^ The resting ECG will be also used to estimate the incidence of other ECG abnormalities such as left ventricular hypertrophy which is a consequence of elevated blood pressure and possibly of antiretroviral treatment itself and is associated with an increased risk of coronary heart disease.^[86],^^[87]^

In addition to the aforementioned data which are collected on all participating patients (or, in case of ECG and BIA, on all participants enrolled at sites that have access to study-certified ECG and BIA equipment, respectively), additional data will be collected in specific substudies of the SMART study. Substudies address issues of body composition and metabolic complications, HIV transmission risk behaviors, quality of life, and health care utilization. In the Body Composition substudy, fasting blood lipids, glucose, insulin, C-peptide, hemoglobin A1c, and venous lactate will be measured at baseline and during followup. These measurements will be used to monitor changes in cardiovascular risk factors and the development of type II diabetes. Fasting serum will be stored for future metabolic research at baseline and annually. Dual Energy X-ray Absorptiometry (DEXA) and 3-cut abdominal CT scans will be performed at baseline and annually to determine body composition. Body habitus will be assessed at baseline and annually. In the HIV Transmission Risk Behavior substudy, a self-administered questionnaire will be obtained at baseline, month 4, and annually to determine whether either treatment strategy is associated with a change in participation in behaviors that would place the participant at risk for transmitting HIV. Participants are tested for sexually transmitted diseases. In the Quality of Life and Healthcare Utilization substudy, healthcare utilization data will be collected at baseline and annually for patients at selected sites. The sites were chosen to achieve a demographically diverse group of patients who obtain healthcare in different types of settings. Quality of life will be measured through a self-administered quality of life instrument. These data will be used to compare the DC and VS strategies for health care utilization and for quality of life changes and will be combined to measure the cost-effectiveness of each strategy.

### 2.6.7. The Need for the Trial to Accommodate New Agents and New Classes of Antiretroviral Agents during the Study

A long-term strategy trial like the SMART study cannot impose restrictions on existing or new therapies that may benefit patients. Therefore, all available antiretroviral drugs may be utilized for patients in both treatment groups. New drugs and new classes of drugs that become available may also be immediately incorporated into the study. There are also no restrictions on ancillary treatments. Patients in trials of immunomodulatory treatments that require concurrent use of antiretroviral treatment should not be enrolled in SMART because such trials are inconsistent with the DC strategy. New treatments could be used immediately in the VS group for viral suppression and could be used in the DC group when risk of disease progression increases, i.e., when CD4+ cell count declines to < 250 cells/mm^3^.

## 2.7. Preliminary Assessment of Percentage of Patients with HIV that Are Potentially Eligible

In preparation for the SMART study, a census of patients was conducted by selected sites at CPCRA units. Over a 2-day time period, 1,118 patients were seen. The patients were broadly representative of persons under care at CPCRA units: 26% were female, 19% were Hispanic, and 51% were African American. Based on their most recent CD4+ cell counts, 475 (42%) would be eligible for the SMART study. The treatment histories for these 475 patients are summarized below:

- 100 (21%) were not taking antiretroviral drugs. Half of these patients were antiretroviral naïve.
- 48 (10%) were taking nRTIs only, and 40% of these patients had HIV RNA levels below the level of detection.
- 183 (39%) were taking nRTI(s) and PI(s), and 58% of these patients had HIV RNA levels below the level of detection.
- 88 (19%) were taking nRTI(s) and an NNRTI, and 67% of these patients had HIV RNA levels below the level of detection
- 48 (10%) were taking nRTI(s), PI(s), and an NNRTI, and 44% of these patients had HIV RNA levels below the level of detection
- 8 (2%) were taking other antiretroviral combinations (e.g., two PIs or a PI and an NNRTI).
- 113 patients (24%) reported a history of taking all three classes of drugs.

These preliminary data indicate that a large fraction of patients seen by CPCRA units are eligible for the proposed study and that, of those eligible, many seem to have remaining treatment options at the time of randomization.

## 2.8. Summary of Rationale and Primary Hypothesis of the SMART Study

In the absence of a cure for HIV, it is critical that antiretroviral treatments be used as effectively as possible to prolong disease-free survival. While current approaches aimed at virologic suppression with multi-drug therapy have proven to be very effective in delaying the development of opportunistic diseases, they are associated with adverse events, many of which are as life-threatening as the opportunistic diseases prevented. Evidence from randomized trials is needed to guide the care of HIV-infected individuals. SMART is the first randomized trial aimed at evaluating a new strategy for using antiretroviral treatments on long-term clinical outcomes. Our hypothesis is that the drug-sparing, episodic treatment approach utilized for patients assigned the DC strategy will delay the development of opportunistic diseases and will reduce the incidence of major toxicities compared to the VS strategy.

## 2.9. Agents Used in the SMART Study

This is a study of antiretroviral management strategies as opposed to specific antiretroviral drugs. Antiretroviral treatments will be obtained by prescription from each patient’s health care provider. No medications will be provided by the study. All antiretroviral agents and immunologic modulators, approved or not, are permitted in this study. As new agents become available, there will be no restriction on their use.

## 2.10. Purpose

The purpose of this study is to compare the long-term clinical consequences of two strategies of antiretroviral management:

- the DC strategy (the experimental group), a strategy aimed at conserving drugs through episodic use of antiretroviral treatment for the minimum time to maintain CD4+ cell count ≥250 cells/mm^3^

versus

- the VS strategy (the control group), a strategy aimed at suppressing viral load as much as possible immediately following randomization and throughout follow-up, irrespective of CD4+ cell count.

The following schematic gives a brief overview of the study design.

Figure 2.	SMART Study Overview

# 3. Methodology

## 3.1. Study Design

This is a multicenter, randomized clinical trial comparing two treatment strategies over 6-9 years of follow-up for delaying disease progression or death. (The events constituting “disease progression” are opportunistic events that are consistent with the 1993 CDC expanded surveillance definition for clinical AIDS.^[88]^ These events are defined in the *CPCRA Clinical Events Handbook* and in a paper describing event documentation and review procedures*.*^[89]^)

Patients will be enrolled over a 3.5-year period. The study will not be blinded to patients and participating clinicians. However, the review of clinical endpoints by the CPCRA Clinical Events Committee, the reading of ECGs, DEXA and CT scans, and laboratory measurements will be performed without knowledge of the patient’s treatment assignment. The study will be event-driven. That is, following a 3.5-year enrollment period, follow-up will extend as long as necessary to obtain the required 910 primary events (see section 3.5).

The VS group is considered to be the control group. While there is no evidence from randomized trials with clinical endpoints that treatment primarily aimed at virologic suppression has long-term clinical benefit in a population like that to be studied (those with CD4+ cell counts > 350 cells/mm^3^), it is a strategy of treatment consistent with current guidelines based largely on expert opinion, one that is widely used, and one that has resulted in substantial declines in morbidity and mortality compared with the standard of care 4 years ago.Error: Reference source not found

This study is broadly inclusive – essentially all adults with HIV who have CD4+ cell counts > 350 cells/mm^3^ are eligible if they are considered candidates for antiretroviral therapy. This inclusiveness will allow evaluation of the DC and VS strategies across all stages of HIV disease. Data collection for this study has been kept to a minimum, with a focus on the unanswered questions about long-term clinical effectiveness.

The DC and VS strategies are expected to result in different plasma HIV RNA level and CD4+ cell count profiles over follow-up. For example, early in the study, by design, it is expected that average plasma HIV RNA levels will be higher in the DC group than in the VS group. In addition, the rate at which patients first reach a CD4+ cell count < 250 cells/mm^3^ is expected to be greater in the DC than VS group. Later in follow-up, these differences may reverse as a consequence of the greater conservation of drugs in the DC group.

The principles that will guide the management of patients in the DC and VS groups are briefly described below and in more detail in section 3.7.

## 3.2. Study Objectives

### 3.2.1. Primary Objective

To compare the DC group with the VS group in delaying clinical disease progression or death.

### 3.2.2. Secondary Objectives

1. To compare the DC group with the VS group for the following:

- Survival
- Incidence of major cardiovascular and metabolic complications
- Incidence of serious disease progression events
- Combined endpoint of clinical disease progression, major cardiovascular and metabolic complications, or death
- Grade 4 adverse events
- Self-reported changes in body appearance
- Adherence to antiretroviral treatment, averaged over follow-up
- Disease progression, death, and other outcomes above within subgroups defined by:
- treatment and viral load status at entry: (1) those not taking antiretroviral therapy at the time of randomization, for whom the initial interventions are deferred versus immediate treatment; (2) those taking treatment with suppressed viral loads, for whom the initial interventions are stopping treatment versus continuing treatment; and (3) those taking treatment with viral loads that are not suppressed, for whom the initial interventions are stopping treatment versus changing treatment
- drug history (naïve; exposure to one, two, three, or more classes of antiretroviral drugs)
- genotypic resistance pattern at baseline
- nadir CD4+ cell count
- CD4+ cell count at baseline
- plasma HIV RNA level at baseline
- slope of CD4+ cell count and plasma HIV RNA prior to enrollment
- calendar date of enrollment
- age, gender and race/ethnicity
- hepatitis B and C serology

2. In subsamples of patients, to compare the DC and VS groups for:

- Major cardiovascular complications, including fatal and nonfatal myocardial infarction, ECG evidence of left ventricular hypertrophy, major arrhythmias, and complete heart block or incident left or right bundle branch block (LBBB and RBBB)
- Prevalence at selected time points of multi-drug resistant (MDR) HIV, and rate of developing MDR HIV
- Fasting total, LDL, VLDL and HDL cholesterol; triglycerides, glucose, insulin, C‑peptide, hemoglobin A1c, and venous lactate
- Body cell mass by BIA
- Body composition
- Quality of life
- Development of targeted symptoms
- HIV transmission risk behaviors
- Health care utilization and costs

3. To conduct nested case-control studies in subsamples of patients on predictors of survival and clinical disease progression, using the frozen plasma from baseline and follow-up visits.
4. To study predictors (e.g., baseline and follow-up immunologic and virologic measures) of survival, disease progression, and major cardiovascular and metabolic complications.
5. To assess the extent to which clinical endpoint differences (survival, clinical disease progression and major cardiovascular and metabolic complications) between the VS and DC groups, if present, are explained by differences in CD4+ cell count, HIV RNA levels, HIV resistance patterns, and other risk factors over follow-up.

### 3.2.3. Tertiary Objectives

1. To ensure that there is adequate power to test the primary hypothesis of the SMART study, the DC and VS treatment strategies will be compared for the following explanatory variables, overall and for subgroups defined by drug history, genotypic resistance pattern, nadir CD4+ cell count, baseline CD4+ cell count, and baseline HIV RNA level:

- HIV RNA levels over follow-up
- CD4+ cell counts over follow-up
- Time to CD4+ cell count < 250 cells/mm^3^ during follow-up
- Timing of use of antiretroviral drugs and drug classes (e.g., time to the initiation or re-initiation of treatment in the DC group, the use of drugs in the DC group before the CD4+ cell count declines to below 250 cells/mm^3^ or delay in the use of new drugs in the VS group in the presence of an elevated viral load)
- Fraction of total follow-up period in which antiretroviral treatment is taken
- Type of antiretroviral drugs regimens used (number of drugs and classes)

2. Among patients in the DC group on antiretroviral treatment at baseline, to determine how CD4+ cell count and HIV RNA level change and the time to reinitiation of antiretroviral therapy vary according to baseline CD4+ cell count and HIV RNA level, nadir CD4+ cell count, genotypic resistance pattern, antiretroviral treatment history and antiretroviral treatment at entry.
3. Among patients in the DC group who are not on antiretroviral treatment at baseline, to estimate the rate of decline of CD4+ cell counts until antiretroviral treatment is initiated, and time to initiation of antiretroviral therapy.
4. To assess the success of enrolling antiretroviral treatment-naïve patients, which, for this subgroup of patients enrolled in the SMART study, is a trial of immediate versus deferred therapy (when to start).

## 3.3. Study Endpoints

### 3.3.1. Primary Endpoint

Time to disease progression or death. (The events constituting “disease progression” are opportunistic events that are consistent with the 1993 CDC expanded surveillance definition for clinical AIDS.Error: Reference source not found These events are defined in the *CPCRA Clinical Events Handbook* and in a paper describing event documentation and review procedures.Error: Reference source not found)

### 3.3.2. Other Major Endpoints

Other major endpoints include:

- Survival
- Time to major cardiovascular events: myocardial infarction, coronary artery disease requiring treatment or an invasive procedure, or stroke. (These events are defined in the *CPCRA Supplemental Events Handbook.*)
- Time to “serious” disease progression event, including death. A serious event for this protocol is defined as one of the following: progressive multifocal leukoencephalopathy, lymphoma, visceral Kaposi's sarcoma, AIDS dementia complex, toxoplasmosis, histoplasmosis, cryptococcosis, MAC, wasting syndrome, and cytomegalovirus disease.^[90]^
- Time to major cardiovascular or metabolic events: myocardial infarction (based on review of hospital records or serial ECG changes indicating major Q wave changes), coronary artery disease requiring treatment or an invasive procedure, myocarditis or pericarditis, stroke, diabetes mellitus, pancreatitis, lactic acidosis, or osteonecrosis (These events are defined in the *CPCRA Supplemental Events Handbook.*) Initiation of drug treatment for hypertension and hyperlipidemia will also be recorded.
- Time to disease progression event, major cardiovascular event, major metabolic event, or death
- Time to the development of any grade 4 adverse event
- Change in self-reported body appearance
- Time to developing new ST-T wave abnormalities, left ventricular hypertrophy, major arrhythmias, heart rate, heart rate corrected QT interval, complete heart block, LBBB, and RBBB based on the resting ECG
- Health care utilization and costs
- Change in total, LDL, VLDL, and HDL cholesterol; triglycerides, glucose, insulin, C-peptide, hemoglobin A1c, venous lactate, and in body cell mass as measured by BIA.
- Change in quality of life
- Symptom severity
- Change in HIV transmission risk behaviors
- Change in fat distribution as assessed by DEXA and CT scans
- Self-reported adherence to antiretroviral drugs
- Development of MDR HIV drug resistance (evidence of resistance to 2 or more classes of antiretroviral drugs)
- Retroviral rebound syndrome

## 3.4. Randomization

Eligible patients will be randomized in a 1:1 ratio to either the DC group or the VS group. Randomization will be stratified by clinical site. Randomization schedules will be constructed to ensure that approximately equal numbers of patients are assigned each treatment within each clinical site.

## 3.5. Sample Size and Statistical Considerations

Sample size is estimated at 6,000 patients. The assumptions made in determining sample size to address the primary objective of the study for the endpoint disease progression or death are detailed below:

1. The primary analysis will be intent-to-treat, using the stratified log-rank statistic with strata defined by the participating sites.
2. Type 1 error is 0.05 (two-sided), and Type 2 error is 0.20 (power = 0.80).
3. As a consequence of deferring treatment until the CD4+ cell count declines to below 250 cells/mm^3^, there will be a 20% difference in the primary endpoint (disease progression or death) rate between the DC group and the VS control group.
4. Disease progression events will account for 70% of the primary endpoints. The remaining events will be deaths. This estimate is based on experience to date in the CPCRA NvR study. This may be an overestimate as the patient population in the NvR study was more advanced at entry than that to be included in the SMART study. If it is assumed that one-half of these deaths will be unrelated to HIV and to the treatment strategies under evaluation and if all events are counted (as is planned), the expected treatment difference will be reduced from 20% to 17% (hazard ratio of 0.83).
5. Based on these assumptions, 910 primary endpoints must be observed.
6. Patients with HIV-1 infection and CD4+ cell counts > 350 cells/mm^3^ are to be enrolled. Event rates over 6-9 years for such individuals are not available except for early studies before more potent antiretroviral treatment was available. Two short-term clinical endpoint studies in advanced patients indicate that protease inhibitor-containing regimens will reduce rates by 50%.^[91],^^[92]^ In the pooled analysis of all patients in AZT monotherapy trials and with CD4+ cell counts ≥ 350 cells/mm^3^, the cumulative 5-year rate of disease progression was 25%.^[93]^ Thus, if the clinical benefits of protease inhibitors among patients with advanced disease apply to patients enrolled in the SMART study, a 5-year rate of 12.5% would be expected. Data from two other sources are consistent with this projection.^[94], Error: Reference source not found^ Data from the EuroSIDA project indicate that the annual rate of disease progression since 1997 among patients with CD4+ cell count ≥ 300 cells/mm^3^ is 1.3 per 100 person years. In another clinical trial of 2,527 asymptomatic patients with CD4+ cell counts 300 to 549 cells/mm^3^, the rate of HIV progression or death after an average of 2.3 years of follow-up was 1.8 cases per 100 person years.^[95]^ If cases of recurrent oropharyngeal candidiasis are excluded (these events would not be considered endpoints in the SMART study), the rate in this study is slightly higher (1.6 per 100 person years) than the annual rate estimated from EuroSIDA data. It is unclear how these rates will change with longer follow-up; however, given the rates of virologic failure being reported with protease inhibitor treatment, one would expect at least modest increases with further follow-up. An increase in the annual rate to 2.6% from 1.3% for years 3 through 7 would result in a 5-year cumulative event rate of 10% and a 7-year cumulative rate of 15%. These event rates were assumed for the SMART study.
7. Two percent of patients will be lost to follow-up each year. It is recognized that if the loss rate is as high as 2% per year then estimates of treatment differences for major endpoints could be severely biased. (Since the loss rate is approximately the same as the expected primary event rate, small differences in the rate of events that would have been observed among the losses could result in a different finding.) Nevertheless, this conservative adjustment to sample size was made in order to increase power because some losses are inevitable.
8. Patients will be enrolled over a 3-year period and followed for a minimum of 6 years; average follow-up will be 7.5 years and will range from 6 to 9 years (if enrollment extends to 3.5 years as currently planned -- a change from the initial version of the study --, the average follow-up may be nearly 8 years).

Important determinants of sample size are the hypothesized treatment difference, the expected event rate in the control group, and the duration of follow-up. The influence of these parameters on sample size is evident from Table 6, which shows the sensitivity of sample size to variations in the hypothesized event rate and the control group event rate.

Table 6

| **Sample Size Required for the SMART Study:**  **Influence of Hypothesized Treatment Difference**  **(expressed as a hazard ratio) and Control Group Event Rate***  **(expressed as cumulative percent after 5 years)** | | | |
| --- | --- | --- | --- |
|  | **Cumulative 5-Year Event Rates** | | |
| **Hazard Ratio** | **7.5%** | **10%** | **12.5%** |
| 0.83 | 7,600 | 5,800 | 4,700 |
| 0.80 | 5,400 | 4,100 | 3,300 |
| 0.75 | 3,400 | 2,600 | 2,100 |
| * assumes increasing hazard after year 2; 3-year enrollment; 6 years minimum follow-up; 2% loss-to-follow-up per year | | | |

Based on the assumptions cited above and the estimates in Table 6, 6,000 patients were set as the enrollment target for the SMART study, 3,000 in the DC group and 3,000 in the VS group.^[96]^ If more patients are enrolled in the enrollment period than planned, average follow-up may be shorter than 7.5 years if the event rate estimates are accurate; the converse may be true if fewer patients than planned are enrolled. Since this is an event-driven trial, the study will continue until 910 events occur in order to provide the desired power.

As described in section 2.6.6, additional data will be collected in substudies, addressing issues of body composition and metabolic complications, HIV transmission, healthcare utilization, and quality of life. In addition, the stored specimens for resistance testing will be used to estimate the prevalence of MDR HIV and the rate of acquiring MDR HIV. The sample size requirements of the substudies on body composition, HIV transmission, quality of life, and healthcare utilization are detailed in section 7. To estimate the prevalence of MDR HIV at an annual visit, it is estimated that 800 stored samples will require analysis for HIV resistance (400 per treatment group). With this sample size, a 5 to 10% difference between the two treatment groups in the percentage of patients with MDR HIV can be detected with 80% power at the 0.05 level of significance (2-sided) if the overall prevalence is 30 to 50%.

## 3.6. Patient Selection

A heterogeneous group of patients with respect to baseline characteristics and treatments used in a pragmatic trial like the SMART study is a strength. It will permit risks and benefits of treatments to be assessed in a variety of patients and will ensure that the trial reflects clinical practice with respect to the type of antiretroviral drugs, opportunistic infection prophylaxes, and immunomodulator and other treatments used.

As a general guideline, patients should be in reasonably good health at the time of study entry, with a Karnofsky performance score ≥ 60 (indicating they do not require considerable assistance and frequent medical care) and a perceived life expectancy of at least 6 months. The patient should be able, in the clinician's opinion, to comply with the protocol (i.e., be willing to accept and comply with the assigned treatment strategy).

### 3.6.1. Inclusion Criteria

1. Signed informed consent
2. Evidence of HIV infection (positive ELISA and Western Blot and/or documented history of measurable HIV RNA)
3. Age > 13 years
4. Current CD4+ cell count > 350 cells/mm^3^ (within 45 days prior to randomization)
5. Willing to initiate, modify, or stop antiretroviral therapy, in accordance with the randomized assignment
6. If participating in sexual activity that could lead to pregnancy, willingness to use acceptable contraception methods

### 3.6.2. Exclusion Criteria

1. Current participation in the CPCRA FIRST, MDR-HIV or another study which is not consistent with one of the treatment groups in the SMART study (e.g., ESPRIT or SILCAAT).
2. Current pregnancy^[97]^ or breastfeeding

## 3.7. Study Plan

This study will include (1) antiretroviral-naïve patients; (2) patients who previously were prescribed therapy but are not on drug at the time of randomization; (3) patients taking antiretroviral therapy at the time of randomization with suppressed viral loads; and (4) patients taking antiretroviral therapy at the time of randomization with viral loads that are not suppressed.

The management of patients throughout the course of the trial will be guided by their assigned strategy. The assigned strategy will guide the initiation of antiretroviral therapy (where applicable) and treatment changes immediately following randomization (where applicable) as well as all changes in antiretroviral therapy during follow-up (i.e., all rounds of therapy).

The sections below give guidelines for antiretroviral management in the DC and VS groups and the data collection requirements prior to randomization and during follow-up.

### 3.7.1.  Treatment Management Guidelines at Randomization

Treatment management guidelines for the DC and VS groups were developed based on two principles: (1) for the DC group, short-term risk of opportunistic events is very low until the CD4+ cell count drops below 250 cells/mm^3^, irrespective of the HIV RNA level; therefore, deferral of therapy while CD4+ cell counts are above this level may conserve long-term treatment options; and (2) for the VS group, ongoing HIV replication leads to immune system damage and to an increased risk of opportunistic diseases; therefore, therapy should be aimed at suppressing viral load levels irrespective of the CD4+ cell count. Management guidelines are summarized in table 7 and detailed below.

Beginning immediately following randomization (top of Table 7), patients in the DC and VS groups are to be managed differently.

a) **For patients assigned to the DC group:**

- **If not taking antiretroviral treatment** (either naïve or previously exposed to antiretroviral treatment), treatment should be deferred until the CD4+ cell count declines to < 250 cells/mm^3^.
- **If taking antiretroviral treatment,** antiretroviral treatment should be stopped until the CD4+ cell count declines to < 250 cells/mm^3^. At a minimum, CD4+ cell counts and viral load levels should be measured at 1, 2, 4, 6, 8, 10, and 12 months following discontinuation of therapy (randomization) and every 4 months thereafter. Also, the treatment half-lives of the drugs taken should be considered in the discontinuation of therapy.

b) **For patients assigned to the VS group:**

- **If not taking antiretroviral treatment**, treatment aimed at virologic suppression should be immediately initiated

.

- **If taking antiretroviral treatment**, the current regimen should be continued if the viral load is suppressed. If the viral load is not suppressed, treatment should be changed with a goal of achieving virologic control.

The degree of virologic suppression is at the clinician’s discretion. A detectable viral load as a trigger for changing therapy is meant to be a guideline. Depending on the patients’ treatment histories and clinical states, clinicians may choose to observe some patients with low but detectable HIV RNA levels (e.g., < 5,000 copies/mL). The level of achievable viral suppression will vary based on the individual patient and his/her treatment history. For example, for a patient on his/her initial antiretroviral regimen, virologic suppression below the level of detection is generally achievable, while for a patient on a 3^rd^ line or salvage regimen it may not be. As a general guideline, the viral load should be maintained as low as possible, preferably below the lower limit of the detection of the assay used.

### 3.7.2. Treatment Management Guidelines During Follow-up

Patients in the DC and VS groups are also to be managed differently during follow-up (bottom of table 7).

a) **For patients assigned to the DC group:** Antiretroviral treatment should be deferred until the CD4+ cell count declines to < 250 cells/mm^3^. In general, when the CD4+ cell count declines to < 250 cells/mm^3^, treatment should be initiated as soon as possible following confirmation. The cutoff of 250 cells/mm^3^ is intended as a guideline. Some patients may require a change in therapy before the CD4+ cell count declines to < 250 cells/mm^3^. For example, clinicians may choose to modify therapy for patients who develop symptoms (e.g., oral thrush, significant unexplained weight loss, unexplained fever) or who have a CD4+ percentage < 15% of lymphocytes. If the CD4+ cell count declines to less than 200 cells/mm^3^, PCP prophylaxis should be initiated (see U.S. Public Health Service and Infectious Disease Society of America guidelinesError: Reference source not found). If therapy is started before the CD4+ cell count declines to < 250 cells/mm^3^ or if it is not started when the CD4+ cell count falls below 250 cells/mm^3^, the reasons for deviation from these general guidelines should be documented in the patient’s record and on the required case report forms. In general, the goal is to defer the use of antiretroviral treatments as long as risk of opportunistic disease is low, which should be guided by the absolute CD4+ cell count.

When the CD4+ cell count declines to < 250 cells/mm^3^, as appropriate, a second CD4+ cell count should be obtained for confirmation within 4 months. Treatment should be reinitiated as soon as possible after the confirmatory CD4+ cell count is available. In some cases, (e.g., when the CD4+ cell count drops rapidly to < 100 cells/mm^3^), confirmation is not necessary for initiation of treatment. Treatment should be reinitiated (or initiated for those not on treatment at entry) with a goal of achieving virologic suppression in order to increase the CD4+ cell count to above 350 cells/mm³. For patients who stopped therapy when their viral load levels were below the level of detection, the regimens previously used may be re-initiated when the CD4+ cell counts decline to < 250 cells/mm^3^. For those who stopped when their viral load levels were not suppressed, different regimens may be more likely to achieve virologic suppression.

These principles are to be followed throughout the entire study as modifications in antiretroviral therapy are considered. Thus, if as a consequence of initiating or reinitiating antiretroviral treatment, the CD4+ cell count increases to above 350 cells/mm^3^, therapy should again be discontinued. **Discontinuation of therapy should not occur until the CD4+ cell count is confirmed to be above 350 cells/mm^3^, i.e., the patient has two consecutive values > 350 cells/mm^3^ obtained at least 2 months apart but not more than 4 months apart. The second CD4+ cell count > 350 cells/mm^3^ may be obtained at a regular follow-up visit or in between data collection visits. At the latest, it should be obtained at the next regular follow-up visit after the 2-month interval.** Therapy should be discontinued after the confirmatory CD4+ cell count becomes available.

While not recommended, clinicians may choose to maintain some patients on successful antiretroviral regimens for a longer period of time (i.e., after the patient’s CD4+ cell count has increased to > 350 cells/mm^3^) before withdrawing therapy in order to maximize the immunologic response. In some patients the CD4+ cell count may decline rapidly to < 250 cells/mm^3^ when therapy is discontinued. For these patients, clinicians may choose to continue therapy indefinitely with a goal of virologic control in order to maintain elevated CD4+ cell counts. Reasons for not discontinuing therapy when the CD4+ cell count is > 350 cells/mm^3^ should be documented in the patient’s record and on the required case report forms.

If the CD4+ cell count does not increase to above 350 cells/mm³ with the regimen prescribed, the treatment regimen may be modified.

**b)	For patients assigned to the VS group:** Antiretroviral treatment should be used following randomization with the goal of achieving viral suppression. Components of the regimen may be changed for toxicities, convenience, or other reasons. If the viral load increases, the treatment regimen should be changed as soon as possible following confirmation (see section 4.4) in order to achieve virologic control. Confirmatory viral load determinations must be carried out within 4 months of the original viral load increase. A treatment change should be instituted as soon as possible after the confirmatory viral load is available.

While not recommended, clinicians may choose to maintain some patients with few remaining drug options on regimens which are not fully suppressive, or temporarily discontinue therapy. Reasons for deviation from the general guidelines for modifying therapy in the presence of an elevated viral load should be documented in the patient’s record and on the required case report forms.

Table 7

| **AR Management in the DC and VS Groups** | |
| --- | --- |
| **DC Group** | **VS Group** |
| **Immediately after randomization:**  ***If not currently on AR treatment:***   - *Defer* treatment until CD4+ < 250 cells/mm^3^   ***If on AR treatment:***   - *Immediately* stop treatment until CD4+ < 250 cells/mm^3^ | **Immediately after randomization:**  ***If not currently on AR treatment:***   - *Immediately* initiate treatment   ***If on AR treatment:***   - **If viral load is suppressed^a^,** continue current regimen - **If viral load is not suppressed,** *immediately* change regimen to achieve virologic control |
| **Management during follow-up**  ***While CD4+ ≥ 250 cells/mm^3^:***   - *Defer* use of drugs until CD4+ <250 cells/mm^3^   ***When CD4+ < 250 cells/mm^3^:*^b^**   - *Initiate* a regimen to achieve virologic control in order to increase the CD4+ to >350 cells/mm³   ***When CD4+ rebounds to > 350 cells/mm^3^:*^c^**   - Stop treatment until CD4+ < 250 cells/mm^3^. | **Management during follow-up**  ***While viral load is suppressed:*^a^**   - Continue current regimen   ***When viral load is not suppressed:***   - Change regimen to achieve virologic control |

^a^ The degree of virologic suppression is at the clinician’s discretion. Clinicians may choose to observe some patients with detectable HIV RNA levels < 5,000 copies/mL.

^b^ Some patients may require a change in therapy before the CD4+ cell count declines to < 250 cells/mm³ if symptoms develop or if the CD4+ percentage is < 15%.

^c^ Clinicians may choose to maintain some patients on successful AR regimens after achieving CD4+ cell count rebounds of > 350 cells/mm³.

### 3.7.3. Baseline Screening and Enrollment

All patients will have the following information and measurements collected before randomization (within 45 days unless otherwise specified):

- Targeted health history, including HIV and supplemental diagnoses, clinical evaluation, and concomitant treatments (e.g., prophylaxes for opportunistic infections and immunomodulator treatment)
- For women of childbearing potential, a documented negative pregnancy test result on a specimen (serum or urine) within 14 days prior to randomization.
- CD4+ cell count and CD4 % (determined locally). (The CD4+ cell count that is used to determine eligibility may also be used as the baseline CD4+ cell count.)
- Nadir CD4+ cell count and apogee HIV RNA level available in the medical record
- Nadir CD4+ cell count and apogee HIV RNA level by patient self-report
- HIV RNA level (determined locally)
- Plasma sample for genotypic resistance testing
- Serum sample for total cholesterol, triglycerides, LDL , HDL, and VLDL cholesterol (no fasting requirements^[98]^)
- Three most recent CD4+ cell counts, CD4 %, and viral load measurements (prior to the baseline measurements) if available in the medical record
- Antiretroviral treatment history
- Self-reported adherence to antiretroviral therapy (for those prescribed therapy prior to randomization)
- Self-reported changes in body appearance
- Smoking status
- Documentation by laboratory report of hepatitis C and serostatus. (If a patient is known to have serologic evidence of hepatitis C by a positive antibody test or evidence of infection by detection of hepatitis C viral RNA, that information must be recorded. If hepatitis C serostatus is unavailable or negative and performed more than one year prior to screening, a hepatitis C antibody test must be performed.)
- Documentation by laboratory report of hepatitis B serostatus. (If a patient is known to be positive for hepatitis B, either with a positive hepatitis B surface antibody [Anti-HBsAg], indicating immunity, or with two positive measurements of hepatitis B surface antigen [HBsAg] separated by 6 months, indicating chronic infection, at any time in the past prior to screening, that information must be recorded and no further testing is required. Otherwise, the hepatitis B surface antigen [HBsAg], hepatitis B surface antibody [anti-HBsAg], and hepatitis B IgG core antibody [IgG anti-HBc] tests must be performed. If the IgG core antibody test [IgG anti-HBc] is not available locally, a total core antibody test [IgG+IgM anti-HBc] should be performed and documented in its place.)
- Stored plasma sample for future HIV drug resistance testing
- Stored plasma and serum samples for future HIV-related, CPCRA-approved research (requires additional consent)
- Personal identifying information for ascertaining survival status – name, father’s surname, Social Security number, date of birth, state of residence, and state of birth (requires additional consent)
- Resting 12-lead electrocardiogram (collected for all patients at sites with access to study-certified ECG equipment)
- Body cell mass by BIA (collected for all patients at sites with access to study-certified BIA devices)

In subsamples of patients, the following data will be collected:

- Stored sample of PBMCs that are viably cryopreserved for future virologic and immunologic research (requires additional consent)
- Quality of life
- Severity grade of selected symptoms
- Healthcare utilization, insurance status, and income
- HIV transmission risk behavior and test results for selected sexually transmitted diseases (*Neisseria gonorrhea, Chlamydia trachomatis*, and syphilis)
- Stored serum sample for future HHV-8 testing
- Fasting serum samples for total cholesterol, HDL, VLDL, and LDL cholesterol, triglycerides, glucose, insulin, and C-peptide
- Fasting whole blood sample for hemoglobin A1c
- Fasting sample for venous lactate
- Stored fasting serum sample for future metabolic research
- Body habitus assessment by the provider
- Fat distribution based on DEXA and CT scans (within 60 days prior to randomization)
- Waist and hip circumference
- Self-reported physical exercise
- Self-reported history of cosmetic procedures

Patients should remain on the antiretroviral regimen prescribed at the time of the screening examination until after randomization unless toxicities necessitate a regimen change. Within 48 hours prior to randomization, the patient should be contacted to update the baseline history and treatments, if necessary, and to verify that the patient still meets the eligibility criteria and consents to enrollment.

In general, patients should be present at the time of randomization in order to reaffirm interest in the study and their eligibility, and in order to initiate the DC or VS intervention immediately following randomization. If this is not possible for logistical reasons, patients must be seen within 7 days after randomization to initiate the intervention.

### 3.7.4. Patient Follow-up

Patients will be seen for routine follow-up clinical evaluation in accordance with standard CPCRA procedures and schedules. All patients will be followed to a common closing date, estimated to be 6-9 years after randomization but could be longer in order to obtain the required number of endpoints for the study.

Follow-up visits for data collection in both treatment groups will occur at 1, 2, 4, 6, 8, 10 and 12 months and every 4 months thereafter.

At Month 1, the following information will be collected:

- CD4+ cell count, CD4 %, and HIV RNA level (determined locally)
- Interim CD4+ cell counts, CD4 %, and HIV RNA levels obtained since last visit

The following information will be collected at every follow-up visit starting with Month 2:

- Targeted health history
- For women of child-bearing potential, self-reported pregnancy status
- CD4+ cell count, CD4 %, and HIV RNA level (determined locally)
- Interim CD4+ cell counts, CD4 %, and HIV RNA levels obtained since last visit
- Antiretroviral treatment changes

The following additional information will be collected every 4 months:

- Self-reported adherence to antiretroviral therapy

The following additional information will be collected every 12 months:

- Plasma and serum samples for future HIV-related, CPCRA-approved research (requires additional consent)
- Serum sample for total cholesterol, triglycerides, LDL, HDL, and VLDL cholesterol (no fasting requirements^[99]^)
- Self-reported changes in body appearance
- Smoking status
- Resting 12-lead electrocardiogram (collected for all patients at sites with access to study-certified ECG equipment)
- Body cell mass by BIA (collected for all patients at sites with access to study-certified BIA devices)
- Selected concomitant treatments

In subsamples of patients, the following additional data will be collected annually (unless otherwise noted):

- Quality of life and severity of selected symptoms (at Months 4, 8, and 12, and annually thereafter)
- HIV transmission risk behavior and test results for selected sexually transmitted diseases (*Neisseria gonorrhea, Chlamydia trachomatis*, syphilis) (at Months 4 and 12, and annually thereafter)
- Stored serum sample for future HHV-8 testing (at Months 4 and 12, and annually thereafter)
- Stored plasma sample for future HIV drug resistance testing (at Month 1, and every follow-up visit thereafter)
- Stored sample of PBMCs that are viably cryopreserved for future virologic and immunologic research (requires additional consent)
- Healthcare utilization, insurance status, and income
- Fasting serum sample for total cholesterol, triglycerides, LDL, VLDL, and HDL cholesterol, glucose, C-peptide, and insulin (at Months 4, 8, and 12, and annually thereafter)
- Fasting whole blood sample for hemoglobin A1c
- Stored fasting serum sample for future metabolic research
- Fasting sample for venous lactate (at Months 4 and 12, and annually thereafter)
- Fat distribution based on DEXA and CT scans
- Body habitus assessment by the provider
- Waist and hip circumference
- Self-reported physical exercise
- Self-reported history of cosmetic procedures

The procedures described above pertain to follow-up visits for the purpose of data collection. To ensure patient safety, more frequent follow-up visits may be necessary under certain circumstances, such as development of clinical symptoms, low nadir CD4+ cell count prior to enrollment, rapid fall in CD4+ cell count, rapid rise in HIV RNA, or drop of CD4+ percentage to < 15% of total lymphocytes.

- In addition to the data collection described above, certain events described in section 4.10 are to be reported as soon as clinical sites become aware of them and obtain the required documentation specified in the *SMART Protocol Manual of Operations.*

If a patient relocates to a clinic not participating in the SMART study, data collection of items that are considered as part of routine care (e.g., viral load, CD4+ cell count, clinical events) may continue if the patient’s new provider agrees and the patient provides a written consent in the form of a release of information. The *CPCRA Data Collection Handbook* provides guidance on how to collect data for patients who relocate.

# 4. Clinical Management Issues

The following clinical management guidelines apply to both the DC and VS groups.

## 4.1. Choice of Antiretroviral and Other Treatments

All available antiretroviral drugs, approved and experimental, may be utilized for patients in both treatment groups. New drugs and new classes of drugs that become available will be immediately incorporated into the study.

Given that the SMART study is currently planned to have a follow-up duration of 6 – 9 years, and that patients may be cycling off and on antiretroviral therapy during the course of this study, antiretroviral regimen selection should be considered carefully.

For example: The multi-system nature of abacavir (ABC) hypersensitivity reaction (HSR) has led to the HSR being misdiagnosed as an intercurrent medical illness, or the reaction being related to another medication. If symptoms that are consistent with ABC HSR are present, and HSR cannot be ruled out, ABC should be discontinued, even when other diagnoses are possible or more likely. Restarting ABC following a HSR results in a prompt return of symptoms within hours. This recurrence of the HSR may be more severe than on initial presentation and may include life-threatening hypotension and death. If ABC was stopped while patient was experiencing signs and symptoms compatible with HSR, ABC should only be reintroduced with caution and appropriate monitoring. Reintroduction of ABC in patients after treatment interruption, with no preceding symptoms of HSR, has rarely resulted in HSR.

There are also no restrictions on ancillary treatments. However, patients in trials of immunomodulatory treatments that require concurrent use of antiretroviral treatment should not be enrolled in SMART because such trials are inconsistent with the DC strategy. For both treatment groups, U.S. Public Health Service and Infectious Diseases Society of America (USPHS/IDSA) guidelines should be followed for prophylaxes for opportunistic infections.^[100]^

## 4.2. Resistance Testing to Guide Antiretroviral Therapy Changes

When making a change in treatment in either group, resistance testing may be helpful to guide therapy changes (guidelines should be consulted).Error: Reference source not found There are no restrictions on the use of resistance testing for guiding therapy changes.

## 4.3. CD4+ Cell Count and Viral Load Level Monitoring

Patients may be monitored more closely than required by this protocol for data collection (i.e., 1, 2, 4, 6, 8, 10, and 12 months following randomization and every 4 months thereafter). The frequency of non-study visits and measurements of CD4+ cell count and viral load will depend on the patient’s clinical status, nadir CD4+ cell count, stage of HIV disease, and recent CD4+ cell count and viral load changes. More frequent clinical (e.g., eye examinations for patients with a history of CMV) and laboratory monitoring may be indicated if there is a sudden or unexpected drop in CD4+ cell count or if therapy is interrupted.

## 4.4. Confirmation of CD4+ Cell Count and Viral Load Levels Prior to Making a Change in Therapy

In general, when planning a change in therapy based on CD4+ cell count or viral load, it is recommended that the assay be repeated for confirmation prior to a change in antiretroviral therapy. For patients in the DC group, CD4+ cell counts should usually also be confirmed to be below 250 cells/mm^3^ before initiating (or reinitiating) therapy. An exception might be a rapid drop in CD4+ cell count for which a delay in obtaining a confirmatory CD4+ cell count would place the patient at risk of an opportunistic event. For patients in the DC group, a second CD4+ cell count should be obtained to confirm that the CD4+ cell count is > 350 cells/mm^3^ before stopping antiretroviral therapy again. Specific time frames for confirmations are given in section 3.7.

## 4.5. Development of Opportunistic Infections, Bacterial Infections, or Malignancy

Refer to the *CPCRA Data Collection Handbook* for guidelines on the workup of suspected opportunistic events.

## 4.6. Study Drug Toxicity and Grading

It is recommended that all patients be closely monitored for signs and symptoms of drug toxicity. Symptoms and laboratory findings should be graded using the CPCRA toxicity table found in the *CPCRA Data Collection Handbook*.

Changes in antiretroviral treatment due to treatment toxicities may be made at any time in either treatment group. In the DC group, drug substitutions for toxicity should be within the same class whenever possible.

## 4.7. Pregnancy and Breastfeeding

Women are not eligible for enrollment into the study during their pregnancy or while they are breastfeeding. However, they may be randomized after delivery. The current guidelines for the management of pregnant women^[101]^ recommend that HIV infected women refrain from breastfeeding.

HIV-infected women with childbearing potential should receive preconceptional counseling and care as recommended by the American College of Obstetrics and Gynecology.^[102]^ For HIV-infected women, additional issues that require discussion include risk of transmission of HIV to the infant and the risks and benefits of various antiretroviral regimens and interventions.

For women of childbearing potential, a pregnancy test (serum or urine) must be performed whenever pregnancy is suspected.

Women in either treatment group who become pregnant during follow-up should be managed according to principles which have been developed by the U.S. Public Health Service. ^Error: Reference source not found^ This requires a careful assessment of the mother’s HIV disease stage, antiretroviral experience, gestational age, and the risks and benefits of specific antiretroviral regimens or other interventions. These guidelines include various case scenarios, updated data on relevant studies, and a summary of the safety and toxicity of individual antiretroviral drugs during pregnancy. The guidelines are regularly updated and providers utilize them for pregnant women.

**An additional consent form may be required for women who become pregnant in some locations.**

After delivery, women will continue to be followed as per their assigned treatment strategy. Breastfeeding is discouraged.

## 4.8. HIV Transmission Counseling

All patients should receive ongoing counseling regarding prevention of HIV transmission to others. When a patient has an elevated viral load, (e.g., during a treatment interruption or incomplete viral suppression on treatment), a discussion of the possibly increased risk of HIV transmission (sexual and/or mother-to-child) should occur.

## 4.9. Coenrollable Studies

During follow-up patients may enroll in other studies as long as the requirements of the study are consistent with the management strategy to which they are assigned. For example, patients in the VS group may enroll in antiretroviral studies which aim to evaluate new treatments for virologic efficacy at any time.

## 4.10. Event Reporting

In addition to the data collected at regularly scheduled follow-up visits, other important data items are reported when the clinical site becomes aware of the event rather than waiting until the next scheduled follow-up visit. These data are described below.

### 4.10.1.  Initiation and Discontinuation of Treatment in the DC Group

Each time antiretroviral treatment is reinitiated (or initiated the first time for those not on antiretroviral therapy at entry) in the DC group following a decline in CD4+ cell count while not taking treatment, this event should be reported on the required case report forms. In addition, if antiretroviral treatment is initiated prior to the CD4+ cell count declining to less than 250 cells/mm^3^, the reason(s) for re-initiating treatment early should be documented.

Similarly, when treatment is discontinued due to a CD4+ cell count > 350 cells/mm^3^, this event should be documented. Treatment half-lives of the drugs taken should be considered in the discontinuation of therapy. Differential half-lives of antiretroviral agents may have therapeutic implications for interruptions in therapy, particularly when patients are maximally suppressed. For instance, the prolonged half-life of the NNRTIs (efavirenz and nevirapine) could lead to unintentional monotherapy for a short period of time if all components of a potent NNRTI-containing regimen are discontinued at the same time. This may be even more significant in some intracellular reservoirs where the half-life of the NNRTIs may be even longer. The implications of this pharmacokinetic phenomenon are unclear; however, one small study which evaluated a 4 week interruption versus continuous therapy reported the emergence of resistance in 5 patients in the intermittent arm.^[103]^ Clinical trials have used three approaches: 1) stop all treatments simultaneously; 2) stop drugs with longer half-lives prior to those with shorter half-lives; and 3) substitute drugs with short-half lives for those with longer half-lives before stopping all treatments. Currently, there are no data on the optimal approach.

### 4.10.2.  Retroviral Rebound Syndrome

In the event a patient in either treatment group experiences a “retroviral rebound” syndrome, an acute illness that resembles primary HIV syndrome following interruption of therapy, this event should be documented.

### 4.10.3. Disease Progression Events

Events comprising the primary endpoint of the SMART study will be reported for patients in both treatment groups for the duration of the trial irrespective of changes in treatment status. These events should be reported immediately after a working diagnosis of the event has been made and the necessary documentation has been assembled. All events that a patient experiences must be reported, not just the first event.

### 4.10.4. Grade 4 Events, Deaths and Major Cardiovascular and Metabolic Complications

Grade 4 signs and symptoms (grade 4 events other than those related to an HIV disease progression diagnosis or limited to laboratory abnormalities), deaths, and major cardiovascular complications that occur will be reported for patients in both treatment groups for the duration of the trial. These events should be reported immediately following site awareness.

### 4.10.5.  Serious and Unexpected Adverse Experiences

Serious and unexpected adverse experiences associated with the antiretroviral drugs used in the DC and VS groups should be reported to national and international regulatory authorities according to local requirements. The DAIDS Safety Office should be copied on MedWatch reports submitted to the Food and Drug Administration by U.S. sites. It will not be necessary for the DAIDS Safety Office to be copied on reports sent to non-U.S. regulatory authorities. Adverse events reported to sponsors of other studies in which SMART participants might be co-enrolled, also **do not** need to be submitted to the DAIDS Safety Office.

# 5. Evaluation

## 5.1. Data Analysis

### 5.1.1. Plans for Describing the DC and VS Strategies

The DC and VS strategies were defined to result in very different virologic and immunologic profiles and a different pattern of use of antiretroviral drugs over the follow-up period. If this difference does not result, the hypothesis of drug conservation versus virologic suppression will not have been properly addressed. Thus, as part of the ongoing monitoring of this study, the DC and VS strategies will be compared for HIV RNA levels, CD4+ cell counts, and the fraction of follow-up time during which antiretroviral drugs are taken. Longitudinal measurements of viral load and CD4+ cell count will be summarized using measured levels (or log transformed) and using repeated binary assessments (e.g., viral load below the level of assay detection, CD4+ cell count > 250 cells/mm^3^). The duration of treatment deferral will be monitored in the DC group using life-table methods, and the median time of treatment deferral will be estimated. In addition, the number and percentage of patients assigned to the DC group who initiate therapy prior to the CD4+ cell count declining to < 250 cells/mm³ and the number and percentage of patients who do not initiate therapy when the CD4+ cell count declines to < 250 cells/mm^3^ will be monitored along with the reasons for deviating from the general protocol guidelines. Following initiation or reinitiation of treatment, the time to achieving a CD4+ rebound to > 350 cells/mm³ will be estimated. The number and percentage of patients who do not discontinue therapy after repeated CD4+ cell count measurements > 350 cells/mm^3^ will be monitored along with reasons for not doing so. Throughout follow-up the number of different antiretroviral drugs used by the DC and VS groups will be compared.

In the VS group, life-table methods will be used to estimate the time to virologic failure that requires a treatment change. In addition, the number and percentage of patients maintained on therapy with HIV RNA levels above the level of detection and the number and percentage of patients who discontinue treatment for 4 weeks or longer will be monitored along with reasons for doing so.

In addition to the overall comparison, the treatment groups will be compared for three subgroups of patients according to treatment history and baseline viral load level: (1) not on treatment at entry (this provides a comparison of deferral of treatment versus immediate treatment); (2) on treatment and viral load levels below the level of detection of the assay used (this provides a comparison of stopping treatment versus continuing treatment); and (3) on treatment and viral load levels above the level of detection (this provides a comparison of stopping treatment versus changing treatment). Additionally, subgroups defined by treatment history (number of classes the patients has been exposed to), nadir CD4+ cell count, baseline CD4+ cell count, and baseline genotypic resistance profile will be evaluated.

By design, in the early follow-up period, viral load levels should be higher and CD4+ cell counts should be lower for the DC compared with the VS group. This early “favorable” difference for the VS group will come at the expense of using more treatments. If the difference between treatment groups in HIV RNA levels and CD4+ cell counts is not substantial during the first 2 years of the trial, it is unlikely that the study question will be addressed. In this case, early termination of the study will be considered.

### 5.1.2. Plans for Addressing Primary and Secondary Objectives

The primary analysis will be by intention-to-treat, comparing the DC and VS groups. Thus, all patients will be included in the primary treatment comparison, irrespective of whether they adhered to their assigned strategy. Time-to-event methods, including stratified log-rank tests and proportional hazards regression analysis, and Kaplan-Meier cumulative event curves, will be used to summarize the major outcomes of disease progression or death; survival; major cardiovascular and metabolic complications; grade 4 events; and the combined endpoint of disease progression, major cardiovascular complications, metabolic complications or death. As part of these analyses, the proportionality of the hazards over time will be assessed in order to investigate whether there is any evidence of an early treatment difference that favors one group but that is reversed with additional follow-up.

Subgroup analyses for these outcomes will be performed to determine whether the treatment effect (DC versus VS) differs qualitatively across various baseline-defined subgroups. Subgroup analyses will be performed by age, gender and race, calendar date of enrollment (in order to assess the effect of different treatment patterns that may emerge) baseline and nadir CD4+ cell count, baseline HIV RNA level, and antiretroviral treatment history. The consistency of the treatment difference across participating sites will also be assessed. An overall test of heterogeneity will provide evidence of whether the magnitude of the treatment difference varies across baseline subgroups.

Many additional secondary analyses will be carried out aimed at understanding the difference (or lack of difference) in the major clinical outcomes. Many of these analyses take advantage of the additional data collection for specific subgroups of patients on body composition , HIV transmission, quality of life, and HIV drug resistance.

In addition, within each treatment group a number of analyses aimed at predicting changes in CD4+ cell count, viral load, HIV resistance and clinical responses will be carried out. These will include analyses of predictors of CD4+ cell count decline following discontinuation of therapy and CD4+ cell count rebound following initiation or reinitiation of therapy in the DC group, predictors of durable viral load responses in both groups, predictors of developing drug resistance in both treatment groups, predictors of major toxicities in both groups, and the association of proximal CD4+ cell count and viral load level with risk of opportunistic disease.

For both treatment strategies, there are risks of being unable to achieve a virologic and/or immunological response with therapy. Thus, time to viral load > 100,000 copies/mL while on treatment and to a CD4+ cell count < 50 cells/mm³ while on treatment will be closely monitored.

## 5.2. Data Monitoring

### 5.2.1. Monitoring the Implementation of the Protocol by the DSMB and the Protocol Team

As described above, at regular intervals the DC and VS groups will be described with respect to CD4+ cell count, viral load, and antiretroviral treatment changes. In general, the aim of this regular monitoring will be to assess adherence to the assigned strategies. The protocol team will also monitor study enrollment and losses to follow-up. The trial may be terminated or modified for poor enrollment, poor adherence to the treatment strategies, or excessive losses to follow-up. The following guidelines for stopping the trial based on these measures will be used:

- Accrual: failure to recruit more than 500 patients in the first 6 months, 1,000 patients by the end of the first year and more than 2,500 by the end of the second year. If some clinical sites are not eligible to enroll because of funding and/or site registration delays, this criterion may be modified to take account of the different starting dates at different sites.
- Reinitiation of treatment in the DC group: lower bound of 95% confidence interval for the cumulative percent re-initiating treatment at 6 months following randomization exceeds 50%.
- Discontinuation of treatment for 4 consecutive weeks or more in the VS group: lower bound of the one-sided 95% confidence interval for the percent of patients who stop antiretroviral treatment for 4 weeks or more during the first year of follow-up exceeds 10%.
- Adherence to assigned strategy (CD4+ cell count and viral load differences): CD4+ cell count difference between treatment groups favoring the VS group of less than 25 cells/mm^3^ after year 1 and less than 50 cells/mm^3^ after year 3, or a viral load difference between treatment groups favoring the VS group of less than 0.3 log_10_ after year 1, year 2, or year 3.
- Loss to follow-up: 1 year loss-to-follow-up rate > 2.5%, or projected overall 3-year lost-to-follow-up > 10% or an absolute difference between treatment groups of more than 7.5%.
- Missed follow-up visits: 3 or more consecutive follow-up visits missed in the 1^st^ year by more than 2.5% of patients, or an annual missed visit rate > 10%.

### 5.2.2. Monitoring of Treatment Differences in Clinical Outcomes by the DSMB

Data will be monitored by the Division of AIDS DSMB. The DSMB will review interim analyses at least once a year.

During the first year of the study (the “pilot phase”), the DSMB and the Division of AIDS reviewed the study at 6 and 12 months following initiation. These evaluations focused on safety comparisons between the DC and VS groups, adherence to the planned interventions, and the achievement of enrollment targets. Based on these reviews, it was determined that the study should continue to enroll the remaining patients required to address the primary objective of the study.

The DSMB will continue to review the protocol at least once a year and will recommend early termination or modification only when there is clear and substantial evidence of benefit or harm. As a guideline, O’Brien-Fleming boundaries and the Lan-DeMets spending function will be used to monitor the primary endpoint comparison.^[104],^^[105]^ Since this is an event-driven trial (i.e., it is planned to continue follow-up until 910 patients experience a primary endpoint), the information fraction used for defining the boundaries at each interim analysis will be based on the planned number of events, not the planned follow-up duration.

In addition to the primary endpoint, the DSMB will monitor a composite outcome of major cardiovascular and metabolic abnormalities (incidence or death due to: myocardial infarction, including silent myocardial infarction based on the resting ECG, stroke, coronary artery disease requiring surgery, kidney failure, and cirrhosis). These events are similarly severe as many events in the primary endpoint, and it is recommended that the DSMB consider early termination or protocol modification only when the O’Brien-Fleming boundary is crossed for the primary endpoint and the findings for both the primary and the composite cardiovascular, metabolic endpoint are consistent (i.e., both favor the VS group or both favor the DC group). Additional composite outcomes may be considered for stopping or modifying the trial.

With this monitoring approach, it is expected that termination of the trial during the first 3 to 4 years of follow-up is very unlikely because so few primary endpoint events, relative to the number on which sample size and power is based, will have occurred.

# 6. Procedures

## 6.1. Reference Documents

CPCRA study procedures are explained in the *CPCRA Investigator's Handbook*, the *CPCRA Data Collection Handbook*, the *Clinical Events Handbook*, the *Supplemental Events Handbook*, the *SMART Laboratory Handbook*, and the *SMART Protocol Manual of Operations*.

Since our goal is to maintain follow-up on all randomized patients and since it is likely that many patients will move over the course of the study, procedures have been developed for off-site data collection. The CPCRA Data Collection Handbook provides guidance on how to collect data for patients who transfer to other CPCRA units and for patients who transfer to non-CPCRA units.

## 6.2. Data Collection and Monitoring

Study data will be collected on standardized case report forms developed by the CPCRA SDMC or transmitted electronically to designated reading centers (e.g., for ECGs and DEXA and CT scans). It is assumed that most data will be collected during patient visits to healthcare providers. In some instances, it may be necessary to obtain and abstract hospital records. Written permission from the patient is generally required. A sample form authorizing release of medical records and/or information is presented in the *CPCRA Data Collection Handbook*. Study data and case report forms will be made available to CPCRA site monitoring personnel.

At a minimum, all items referenced in the protocol as being relevant to the research study will be recorded in the patient's research record in accordance with standard CPCRA procedures. In addition, all items specifically required by the protocol will be recorded on case report forms. Items that are recommended but not required by the protocol may or may not be recorded on case report forms.

## 6.3. Publications and Presentations

All persons preparing publications and presentations related to data obtained from this study will adhere to the CPCRA Publications and Presentations Policy found in the *CPCRA Investigator’s Handbook*.

## 6.4. Human Subjects

This protocol must receive the approval of the participating site's institutional review board (IRB) prior to implementation. All participants must sign an informed consent form (see sample in Appendix B). The confidentiality of all study participants will be protected in accordance with standard CPCRA procedures. If an amendment that affects data collection is implemented, patients may be enrolled on the previous version for 90 days after the new version is distributed. After 90 days no patients will be enrolled on the previous version. Amendments that do not affect data collection are not subject to this requirement. These and other DAIDS protocol registration policies are articulated in a manual, links to which can be found on the CPCRA and SMART web sites.

# 7. Substudies

This section describes the substudies referred to in section 2.6.6. These substudies address issues of quality of life, of healthcare utilization and cost-effectiveness, of HIV transmission risk behavior, and of metabolic complications. Not all sites will be participating in these substudies. At the sites conducting each substudy, patient participation in the substudies is optional.

## 7.1. Quality of Life and Healthcare Utilization Substudy (CPCRA 065A)

This substudy consists of two distinct components: 1) quality of life, and 2) healthcare utilization and cost-effectiveness. The primary objective of the first component is to compare the DC and VS groups for quality of life. The primary objective of the second component is to compare the two groups for healthcare utilization and cost-effectiveness. Both components are combined into a single substudy because the quality-of-life outcomes will also be used to calculate “quality-adjusted life-years” (QALYs), needed for determining cost-effectiveness. Therefore, quality of life and healthcare utilization will be assessed for the same patient population.

### 7.1.1. Rationale

*Quality of Life*

With advances in antiretroviral therapy, the morbidity and mortality of HIV disease have decreased by as much as 90%. With this improvement in survival, quality of life is increasingly relevant to decisions about antiretroviral therapy. Several factors influence the perceived quality of life: 1) HIV disease progression reduces quality of life^[106]^; 2) effective antiretroviral therapy substantially improves quality of life among persons with symptomatic HIV disease^[107],^^[108]^; and 3) combination antiretroviral therapy has a number of side effects, such as nausea, diarrhea, and body shape changes, which may reduce quality of life. Therefore, quality of life of asymptomatic patients with earlier HIV disease may decrease after the initiation of antiretroviral therapy^[109]^. These same considerations are applicable to changes in antiretroviral therapy. Changes in therapy that reduce clinical symptoms of HIV disease may improve quality of life, but regimen changes also have the potential for new and/or more severe side effects that worsen quality of life.

*Healthcare Utilization and Cost-Effectiveness*

HIV disease is a costly disease to treat and much of the cost is due to the use of antiretroviral drugs, which may differ between the DC and VS groups. However, costs of treating opportunistic infections and other complications of HIV and its treatment are also high.^[110]^ Thus, a treatment strategy that aims at intermittent antiretroviral drug use may be associated with less cost than one in which there is ongoing antiretroviral drug use. However, if the former strategy is associated with more complications associated with HIV disease, then this would be associated with increased costs. It is unclear whether in the long run costs associated with episodic antiretroviral therapy would be more or less than those associated with a strategy using ongoing therapy.

The high costs of treating HIV and the likely differences in costs and potential differences in outcomes between the DC and VS groups suggest that a cost-effectiveness analysis would be very useful to policymakers in the U.S. and abroad. In particular, if the two study arms have similar clinical outcomes, but different costs, it would be socially desirable to choose the less costly approach. Even if one arm were both more effective and more costly than the other, it would still be useful to understand the tradeoffs at stake.

### 7.1.2. Patient Selection

Data will be collected at selected CPCRA sites that are geographically dispersed, have diverse patient populations and represent different systems of care. A total of 1,200 patients will be enrolled in this substudy.

### 7.1.3. Objectives

**Primary Objectives**

1. To compare the DC group with the VS group for quality of life and symptom severity.
2. To compare healthcare utilization and resulting costs for the DC and VS groups.

**Secondary Objectives**

1. To compare the effect of *immediate* (VS group) versus *deferred* initiation (DC group) of antiretroviral therapy on quality of life, on symptom severity, on healthcare utilization, and on the resulting costs among patients who are not on antiretroviral therapy at enrollment.
2. To compare the effect of *continuing* (VS group) versus *stopping* antiretroviral therapy (DC group) on quality of life, on symptom severity, on healthcare utilization, and on the resulting costs for patients who are on antiretroviral therapy at enrollment.
3. To describe longitudinal changes in quality of life, in symptom severity, in healthcare utilization, and in the resulting costs over time for subgroups of patients in the DC group and in the VS group defined by gender, ethnicity, geographical region, socio-economic status and insurance source.
4. To compare QALYs for the DC and VS groups
5. To compare cost-effectiveness for the DC and VS groups

### 7.1.4. Measurements

**Baseline measurements**

- Self-reported quality of life (SF-12, plus visual analog scale)
- Severity of selected symptoms
- Self-reported healthcare utilization (see section 7.1.5)
- Self-reported source of insurance
- Self-reported socio-economic status

**Follow-up measurements**

The following follow-up measurements will be collected annually, unless otherwise noted.

- Self-reported quality of life (SF-12, plus visual analog scale), at Months 4, 8 and 12, and annually thereafter
- Severity of selected symptoms at Months 4, 8 and 12, and annually thereafter
- Self-reported healthcare utilization (see section 7.1.5)
- Self-reported source of insurance
- Self-reported socio-economic status

### 7.1.5. Selected outcome measures

*Quality of Life*

Quality of life will be assessed using a 12-item self-administered questionnaire (SF-12) adapted from the Medical Outcomes Study, and a visual analog scale, used by the NIAID AIDS Clinical Trials Group. The SF-12 assesses 8 dimensions of quality of life: 1) physical functioning, 2) general health perceptions, 3) pain, 4) social function, 5) physical role limitations, 6) emotional role limitations, 7) mental health, and 8) energy. Additional measures for the quality of life will be obtained through a symptom severity survey and an assessment of body appearance. The symptom severity survey assesses common side effects of antiretroviral therapy (nausea, vomiting) as well as symptoms that may indicate HIV disease progression.

*Self-reported healthcare utilization*

Healthcare utilization (excluding medication use) will be collected through a self-administered questionnaire, which asks for healthcare utilization within the last 4 weeks. The questionnaire is based on the HIV Costs and Services Utilization Study (HCSUS) survey instrument.^[111]^ The HCSUS study (conducted by the RAND Corporation) is the principal national source of service utilization information on persons with HIV. Baseline data from that survey have already been used to estimate the costs of care for persons with HIV.^[112]^

The table below describes the data elements that will be collected through the self-administered healthcare utilization questionnaire. All questions are asked with respect to *“the last 4 weeks”*.

Table 8

**Healthcare utilization questionnaire items**

| **Item** | **Question** |
| --- | --- |
| Hospital and emergency room utilization | - How many nights did you stay in a hospital ward (not emergency room)? - How many visits did you make to an emergency room? |
| Nursing home utilization | - How many nights did you stay at a nursing home? |
| Physician utilization | - How many visits did you make to a clinic or office to see a doctor or nurse? |
| Home health care | - How many times has a nurse or other health care provider cared for you at home? - How many days has a family member or friend provided care for you at home? |
| Other | - How many times did you see a social worker or case manager? |

Additionally, participants are asked for the number of missed days of employment, school, housework, etc., to be used for assessing costs associated with their HIV disease.

*Drug utilization*

Prescribed medications will be recorded for all patients in the SMART study. This includes antiretroviral drugs and other HIV-related agents such as prophylactic drugs for opportunistic infections.

*Cost Information*

The main costs of treating HIV/AIDS are the costs of antiretroviral drugs and the hospitalization costs associated with treating opportunistic infections and other complications associated with HIV and its treatment. Additionally, a monetary value will be assigned to all items collected in the *healthcare utilization* questionnaire listed in Table 8.

National data sources will be used to compute national average unit costs for service provision. Site-specific cost data will not be used as there are substantial variations in how sites compute costs and how payers pay. Medicare DRGs and RBRVS values for hospitalizations and physician visits and published data on drug costs will be used in computing the costs for medical care. The cost of unpaid care provided by family or friends, and the cost of patient time, e.g., missed work, missed school or volunteering, will be valued at the average hourly total compensation rate for civilian workers as reported in the base year by the Bureau of Labor Statistics.

*Quality-Adjusted Life Years (QALYs)*

QALYs will be calculated based on the outcomes of the quality of life questionnaire (SF-12). It is expected that an appropriate scaling of the SF-12 questionnaire for calculating QALYs will be available by the end of the study based on ongoing work to validate the SF-12 for calculating QALYs. Otherwise, the QALYs will be calculated using the quality of life assessment on a visual analog scale.^[113]^

### 7.1.6. Subsampling Plan and Data Analysis

**Sample Size**

*Quality of Life*

For sample size calculations, estimates of the standard deviations of the Quality of Life (QOL) dimensions of the SF-12 questionnaire at baseline, and of the changes from baseline to Month 12 were obtained from the IL-2 Study (CPCRA 059). The patient population in the IL‑2 study is comparable to the SMART study, since patients entering IL-2 had a CD4+ cell count of at least 300 cells/mm^3^. The estimates are obtained from the control group in IL-2 (standard antiretroviral medication, no study drug). Values of the standard deviations for the 8 dimensions of the SF-12 questionnaire are given in Table 9.

Table 9

**Mean and Standard Deviation of QOL Dimensions at Baseline, and Standard Deviation of the Change in QOL to Month 12**

**Source: IL-2 Study (CPCRA 059)**

| **QOL dimension** | **Mean at baseline, (possible range)**  **(N=256)** | **SD at baseline**  **(N=256)** | **SD of change in QOL at Month 12 (N=233)** |
| --- | --- | --- | --- |
| General health perception | 3.9 (1-5) | 0.8 | 0.9 |
| Physical functioning | 5.5 (2-6) | 1.0 | 0.9 |
| Role Limitations, physical | 3.6 (2-4) | 0.7 | 0.8 |
| Role limitations, emotional | 3.6 (2-4) | 0.7 | 0.8 |
| Pain | 4.5 (1-5) | 0.8 | 0.8 |
| Mental health | 9.2 (2-12) | 1.7 | 1.8 |
| Energy | 4.3 (1-6) | 1.1 | 1.1 |
| Social Functioning | 4.4 (1-5) | 0.9 | 0.9 |

For each of the 8 quality of life dimensions, the standard deviation at baseline is approximately the same as the standard deviation of the change in quality of life from baseline to Month 12. The sample size for the substudy is calculated to detect a difference in *mean change in quality of life* (between treatment groups) of *17 % of the standard deviation* of the quality of life change.

Table 10 displays the total sample size that is necessary to detect a difference of 25 %, 20%, 17% and 15 % of the standard deviation, with a power of 0.8 and a Type 1 error of 0.05 (significance level). The sample size has been inflated to 1,200 to allow for loss-to-follow-up and missing data.

Table 10

**Sample Size Required to Detect a Difference in Change in Quality of Life Between Two Treatment Groups with Power = 0.8 and Type 1 Error = 0.05**

**Based on SD estimates from the IL-2 Study (CPCRA 059)**

| **Difference in mean QOL to be detected (as % of the SD of change)** | 25 % | 20 % | 17 % | 15 % |
| --- | --- | --- | --- | --- |
| **Sample size** | 506 | 788 | 1090 | 1398 |

*Healthcare Utilization and Cost-Effectiveness*

The sample size for the substudy will detect a difference in *mean costs (per year) associated with HIV disease* (between treatment groups) of *17 % of the standard deviation* of the annual costs (per patient) associated with HIV disease with a power of 0.8 and a Type 1 error of 0.05 (significance level).

The mean cost of treating HIV (per patient per year) was estimated at $22,200 by the HIV Cost and Services Utilization Study Consortium, Bozzette et al.^[114]^ It is assumed that the standard deviation is equal to the mean of the cost. With this assumption, the sample size is calculated to detect a difference of $3,774 in mean costs per patient per year between the DC and VS groups.

Table 11 displays the total sample size that is necessary to detect a difference of 25 %, 20%, 17% and 15 %of the standard deviation, with a power of 0.8 and a Type 1 error of 0.05. The sample size has been inflated to 1,200 to allow for loss-to-follow-up and missing data.

Table 11

**Sample Size Required to Detect a Difference in Costs Between Two Treatment Groups with Power = 0.8 and Type 1 Error = 0.05**

| **Difference in *mean cost* to be detected**  **(as % of the SD of Cost per patient per year)** | 25 % | 20 % | 17 % | 15 % |
| --- | --- | --- | --- | --- |
| **Sample size** | 506 | 788 | 1090 | 1398 |
| **Difference in *mean* cost to be detected (in US$)** | $ 5,550 | $ 4,440 | $3,774 | $3,330 |

**Statistical Methods**

*Quality of Life*

All analyses will be intent-to-treat. All patients with a baseline quality of life score and at least one subsequent measurement will be included in the analyses.

For each quality of life dimension, changes from baseline, and average quality of life throughout follow-up will be compared at fixed time points (at month 4, 8, and 12, and yearly thereafter) for all subgroups. The change in quality of life over time will be described using longitudinal regression. Analyses will be stratified by site.

*Healthcare Utilization*

Healthcare utilization and the resulting costs will be compared between the DC and VS groups using generalized linear models, adjusted for baseline demographics, such as gender, ethnicity, geographical region, and baseline clinical markers.

*Cost-effectiveness*

For the cost-effectiveness analysis, the incremental cost-effectiveness ratio will be used, defined as the ratio between the difference (increment) in mean costs and the difference in effectiveness (QALYs) between the treatment groups. Standard cost-effectiveness methods suggested by Gold et al.,^[115]^ will be used to perform and report the analysis. After performing the analysis for the base case, sensitivity analyses will be conducted by varying key parameters.

Costs and QALYs will be discounted to the base year of the study to account for the time value of money and health. After the costs and quality adjusted life years have been discounted and summed within each arm of the study, cost-effectiveness ratios will be computed.

In order to calculate the costs associated with HIV disease, data on service utilization will be matched to information on the unit cost of services. Elements like the cost of health care provided by family members or cost due to missed work days will be included.

## 7.2. HIV Transmission Risk Behavior Substudy (CPCRA 065B)

### 7.2.1. Rationale

There is increasing attention to the role that persons in HIV care may play in ongoing transmission of HIV.^[116],^^[117]^ The two strategies being evaluated in the SMART trial may have substantially different effects on HIV transmission. Plasma viral loads correlate with risk of perinatal and heterosexual transmission; transmission is very unlikely among persons who have plasma viral load measurements < 1000-1500 copies/ml.^[118],^^[119]^ Therefore, withholding antiretroviral therapy could result in a greater risk of HIV transmission to sexual or needle-sharing partners.

However, HIV transmission is much more complicated than considerations of viral load alone. Transmission risk behavior appears to be affected by the use of antiretroviral therapy. Condom use decreased after initiation of combination antiretroviral therapy^[120]^, and in a recent study, the rate of bacterial sexually-transmitted infections increased after patients started antiretroviral therapy.^[121]^ Outbreaks of syphilis and gonorrhea among HIV-infected persons in the era of combination antiretroviral therapy have now been reported from many cities in developed countries, suggesting a change in sexual behavior related to the availability of antiretroviral therapy.^[122],^^[123]^ Therefore, the use of continuous antiretroviral therapy may not change or may even increase HIV transmission because its use results in an increase in transmission risk behavior. The recent increase in HIV incidence in San Francisco, despite the widespread availability of combination antiretroviral therapy^[124]^ demonstrates the plausibility of this hypothesis - that availability of antiretroviral therapy may have the paradoxical effect of increasing HIV transmission.

Another consideration involving HIV transmission risk is the risk of transmitting a drug-resistant strain of the virus. Primary drug resistance appears to be increasing in the United States and Canada, and primary resistance decreases the initial response to antiretroviral therapy.^[125]^ Relatively high rates of primary drug resistance might reverse the dramatic effects of antiretroviral therapy on the morbidity and mortality of HIV infection. Therefore, transmission risk behavior (unprotected sex or needle-sharing) while on antiretroviral therapy carries with it the risk of transmission of drug-resistant HIV.

The SMART trial offers an opportunity to study the relationships between use of antiretroviral therapy and HIV transmission risk behavior. Patients will be randomized to two different strategies of antiretroviral treatment. The episodic use of antiretroviral therapy in the DC arm could increase overall transmission risk, by increasing the risk per episode while patients are off therapy and have unsuppressed viremia. The emphasis on maintaining the viral load as low as possible in the VS arm may decrease HIV transmission (by decreasing the per episode risk of transmission). However, if being on antiretroviral therapy increases transmission risk behavior, the VS strategy could be associated with an increased risk of transmission. In particular, prolonged use of antiretroviral therapy may result in carriage and transmission of drug-resistant HIV.

### 7.2.2. Patient selection

Data will be collected at selected participating sites that are geographically dispersed, have diverse populations and represent different systems of care. Six hundred patients will be enrolled.

### 7.2.3. Objectives

**Primary objective**

To compare the DC group with the VS group for HIV transmission risk behaviors

*High-risk HIV transmission risk behavior* will be defined as: 1) self-reported needle-sharing or anal or vaginal sex without a condom, or 2) a positive urine test for *Neisseria gonorrhea* or *Chlamydia trachomatis* or serologic evidence of incident syphilis or HHV-8 infection.

**Secondary objectives**

1. To compare the effect of *immediate* (VS group) versus *deferred* initiation of antiretroviral therapy (DC group) on HIV transmission risk behavior among patients who are *not* on antiretroviral therapy at enrollment.
2. To compare the effects of *continuing* (VS group) versus *stopping* antiretroviral therapy (DC group) on HIV transmission risk behavior among patients who are on antiretroviral therapy at enrollment.
3. To evaluate the correlation between self-reported adherence to antiretroviral therapy and HIV transmission risk behavior for patients on antiretroviral therapy.
4. To compare the DC and VS groups for HIV transmission risk behavior in subgroups defined by age, gender, possible transmission category, HIV RNA level, and baseline genotypic resistance pattern.
5. To evaluate the correlation between self-reported transmission risk behavior and the acquisition of *Neisseria gonorrhea*, *Chlamydia trachomatis*, syphilis and/or HHV-8.
6. To develop analytic techniques to combine behavioral and biological (viral load, presence of antiretroviral drug resistance) data into a measure of overall transmission risk, using prediction equations from other studies.

### 7.2.4. Measurements

HIV transmission risk behavior will be assessed in two ways: self-reported behavior and biological testing for four sexually-transmitted infections (gonorrhea, chlamydia, syphilis, and HHV-8).

**Self-reported risk behavior**

Self-reported behavior will be recorded using a self-administered confidential questionnaire, which avoids the bias that may arise from having a questionnaire regarding this sensitive subject administered by study personnel. To minimize recall bias, this questionnaire focuses on sexual behavior in the last two months.^[126]^ The questionnaire distinguishes between steady and occasional sex partners and assesses condom use and knowledge of HIV serostatus of partners for the last episode of sexual intercourse with the steady and/or occasional partner. Injection drug-use and needle-sharing are recorded.

**Biological testing**

To encourage participation, laboratory testing for sexually-transmitted infections will not involve rectal or genital examination or sampling, but will be performed using urine samples, as well as blood samples collected during routine protocol visits. Urine testing using DNA amplification techniques (polymerase chain reaction [PCR], strand displacement) is almost as sensitive as culture or DNA amplification tests of specimens obtained directly from the urethra or cervix (90% versus 97% for *Chlamydia trachomatis*, 94% versus 97% for *Neisseria gonorrhea*) and has greater patient acceptability in a screening program. Importantly, urine testing using DNA amplification techniques is highly specific (98.9 to 100%). ^[127],^^[128],^^[129],^^[130]^ In addition, syphilis and HHV-8 infection will be assessed serologically.^[131]^ Urine tests for gonorrhea and chlamydia, and serum tests for syphilis will be performed locally. Test results will be returned to the patient and care provider so that these treatable conditions can be appropriately managed. Incident syphilis will be defined as a newly-positive treponemal serological test (for example, FTA-ABS) or a greater than 4-fold increase in non-treponemal test (RPR) among those who have a positive serological test at enrollment. HHV-8 serologic testing will be performed at the end of the trial in a central laboratory. Diagnostic tests for HHV-8 are in evolution and rapidly improving, so the assay to be used will be chosen when sample collection has been completed.

Patients who are diagnosed with gonorrhea, chlamydia, or syphilis based on tests performed in this study should be treated using current guidelines^[132]^ and counseled about the need for safe sex.

**Baseline measurements**

- Self-reported HIV transmission risk behavior
- Urine testing for *Neisseria gonorrhea* and *Chlamydia trachomatis* using a licensed DNA amplification test (performed locally)
- Syphilis serology (performed locally)
- Stored serum specimen for future HHV-8 testing

**Follow-up measurements**

The following measurements will be collected at the Month 4 and Month 12 follow-up visits, and annually thereafter for the first 4 years after enrollment:

- Self-reported HIV transmission risk behavior
- Urine testing for *Neisseria gonorrhea* and *Chlamydia trachomatis* using a licensed DNA amplification test (performed locally)
- Syphilis serology (performed locally)
- Stored serum specimen for future HHV-8 testing

### 7.2.5. Subsampling plan and statistical methods

In a recent study of randomly selected patients in HIV care, 22 % reported anal and/or vaginal intercourse without a condom the last time they had intercourse during the past 3 months, or needle-sharing.^[133]^ Similar percentages were reported from two other clinic cohorts.Error: Reference source not found^,^ ^[134]^ Assuming that 22 % is a reasonable estimate for the rate of sexual HIV transmission risk behavior among persons likely to enroll in the SMART study, the sample size is calculated to detect a 10% difference in the proportion of patients with high-risk transmission risk behavior between the two study arms with a power of 0.8 and a Type 1 error of 0.05 (significance level).

Table 10

**Sample size required to detect a 10 % difference in transmission risk behavior**

| Postulated difference | Total sample size required |
| --- | --- |
| 20% vs. 30% | 626 |
| 15% vs. 25% | 540 |
| 10% vs. 20% | 438 |

Patients will be enrolled from selected sites, with the goal of enrolling at least 600 patients.

**Data analysis**

As described in section 7.2.1, HIV transmission risk is affected by 1) viral load, 2) the type and frequency of risk behavior, and 3) the presence of drug-resistant virus. While each of these factors will be measured in this substudy, there is at present no standardized analytic technique for combining these factors into a single metric expressing transmission risk. Ways of combining behavioral and biological data (viral load and resistance testing) into an expression of transmission risk will be explored.

For selected analyses, patients will be categorized into one of three HIV transmission risk categories based on self-reported behavior during the previous 2 months: *high risk* - unprotected anal and/or vaginal sex or needle-sharing, *low risk -* protected anal and/or vaginal sex, protected and/or unprotected oral sex, and *no risk -* no reported sex or needle sharing.

## 7.3. Body Composition Substudy (CPCRA 065C)

### 7.3.1. Rationale

Antiretroviral therapy is commonly associated with lipodystrophy (visceral fat accumulation, breast enlargement, lipomata and peripheral lipoatrophy), and is significantly associated with dyslipidemia (high triglycerides, high cholesterol, low HDL, LDL-B cholesterol), insulin resistance, type 2 diabetes and lactic acidemia.^[135],^^[136],^^[137],^^[138],^^[139],^^[140],^^[141],^^[142],^^[143],^^[144],^^[145]^ The prevalence of these changes ranges from 30-80% in different cohorts, and may be influenced by gender, race/ethnicity, age, viral load, immune function, as well as the type and duration of antiretroviral therapy.Error: Reference source not found^,Error: Reference source not found,Error: Reference source not found,Error: Reference source not found^ Elevated insulin, C-peptide and triglycerides one year after commencement of therapy may be risk factors for increased severity of lipodystrophy.Error: Reference source not found

Body composition techniques used for assessing lipodystrophy and HIV wasting include total body potassium (the gold standard), bio-impedance analysis (BIA), Dual Energy X-ray Absorptiometry (DEXA) and the single-cut abdominal CT scans (L4 Level).Error: Reference source not found^,Error: Reference source not found,Error: Reference source not found,Error: Reference source not found,Error: Reference source not found,^^[146],^^[147],^^[148]^ However, total body potassium remains a limited research-only tool, and regional fat and bone mineral density (BMD) cannot be reliably evaluated with BIA,Error: Reference source not found whereas DEXA is useful for measuring limb fat and regional BMD, and CT for visceral fat. Also, BMD on CT scanning of lumbar spine (which estimates trabecular bone) was significantly reduced in patients with HIV lipodystrophy but not BMD as assessed by DEXA (which estimates cortical bone).Error: Reference source not found Recent evidence suggests that the quality and reproducibility of images with the 3-cut abdominal CT scans are superior to the single cut CT scans with minimal additional radiation, time and costs.^[149]^ Thus 3-cut CT scan (L2, mid and L4 level) can provide valuable information on both fat and bone compartments.^[150],^^[151]^

There are no data on longitudinal body composition changes among treatment naïve patients, and only one 12-week study of body composition changes after treatment interruption.^[152]^

Several protease inhibitor switch studies, most of no more than 6 months’ duration, have largely demonstrated amelioration of the metabolic derangements but no reversal in the body habitus changes.^[153],^^[154]^ However, no study has evaluated the effect of discontinuation of antiretroviral therapy on reversal of these metabolic parameters.

Osteopenia has also been linked to use of combination antiretroviral therapy after adjustment for age, sex, race and body mass index (BMI).Error: Reference source not found^,^^[155],^^[156]^ Associations between visceral fat accumulation and peripheral lipoatrophy and osteopenia have been observed.Error: Reference source not found^,Error: Reference source not found^ Clinically osteopenia has been most related to nucleoside analogue therapy-induced lactic acidemia and to body weight prior to initiation of antiretroviral therapyError: Reference source not found^,Error: Reference source not found^ However, both these studies were cross-sectional. In vitro, protease inhibitors affect osteoblast activity.^[157]^ Various markers of bone formation and resorption (e.g., serum osteocalcin, parathyroid hormone (PTH), ionized calcium, 25 OH vitamin D, bone specific alkaline phosphatase, and collagen type I cross linked N-telopeptide, collagen type I cross linked C-telopeptide) have been studied in the HIV and non-HIV population with conflicting data.Error: Reference source not found^,Error: Reference source not found,^^[158],^^[159]^

Lactic acidosis and acute hepatic failure occur (about 1:1000) rarely and unpredictably with nucleoside analogue therapy.Error: Reference source not found^,Error: Reference source not found,^^[160]^ Symptomatic lactic acidemia without liver dysfunction was found in about 5% of adults receiving nucleoside analogue therapy in one cohort and asymptomatic lactic acidemia in 10-36% of such adults.Error: Reference source not found^,Error: Reference source not found^ The significance of lactic acidemia in the absence of acidosis and hepatic failure is not clear, but lactic acidemia is also associated with lipodystrophy, osteopenia, myopathy, peripheral neuropathy and a postpartum neurological disease in infants.Error: Reference source not found^,Error: Reference source not found,Error: Reference source not found^ Few data (none randomized) are available on lactate levels among patients receiving or discontinuing antiretroviral therapy.

Changes in various endocrine and paracrine factors that influence body composition have been reported to be associated with HIV lipodystrophy and wasting syndromes.Error: Reference source not found^,^^[161],^^[162],^^[163],^^[164],^^[165],^^[166],^^[167]^ Insulin resistance (assessed by various methods) has been associated with body composition changes.Error: Reference source not found^,^^[168],^^[169]^ The Homeostasis Model Assessment (HOMA) for estimating insulin resistance, using single simultaneous fasting insulin and glucose, is a sensitive, reproducible method validated in large epidemiological studies.^[170]^ Significant reductions in mean growth hormone and insulin-like growth factor levels proportional to visceral fat accumulation among lipodystrophic subjects have been found.Error: Reference source not found Similarly, changes in testosterone and thyroid stimulating hormone have been documented in small cross-sectional studies.Error: Reference source not found^,Error: Reference source not found,Error: Reference source not found,Error: Reference source not found^ In a recent study of HIV-infected men treated with protease inhibitors, an inverse relationship was seen between the gonadal steroid levels and hyperinsulinemia; also, an increased BMI and reduced lean body mass predicted hyperinsulinemia.Error: Reference source not found In cross-sectional studies of patients with HIV lipodystrophy the data on significant changes in concentrations of leptin, adiponectin, cortisol, and products of corticosteroid metabolism are conflicting.Error: Reference source not found^,Error: Reference source not found,Error: Reference source not found,Error: Reference source not found^ It is unknown, if these endocrine changes have any etiological role, or are just epi-phenomena to changes in body composition. It is also unknown if these changes are associated with the antiretroviral drugs or the virus itself, or are early predictors of the body composition changes. Therefore, a prospective evaluation of endocrine factors (with and without drug effects as presented in the SMART study), in combination with body composition changes is necessary to better characterize the HIV lipodystrophy.

Until recently, both the diagnostic and severity assessments of lipodystrophy were subjective. An objective case definition of lipodystrophy incorporating 10 clinical, metabolic and body composition variables was developed that can diagnose lipodystrophy with 80% accuracy.Error: Reference source not found Each variable in the definition is assigned a score and, after subtraction of a constant (43), a sum total score of at least zero is diagnostic of lipodystrophy. The proportions of antiretroviral-naïve, antiretroviral-experienced but protease inhibitor-naïve, and protease inhibitor-experienced subjects that were classified as lipodystrophy cases by this case definition were 5%, 39% and 59%, respectively. The lipodystrophy case definition (LDCD) score is a better objective measure of lipodystrophy severity than either DEXA, abdominal CT, or lipid, glycemic and acid-base parameters known to correlate with lipodystrophy severity.Error: Reference source not found Also, and in contrast to DEXA and CT, the LDCD score is also gender-independent, whereas body composition values on DEXA and CT are markedly different between men and women. Changes in lipodystrophy prevalence and severity over time have not been well evaluated. Two prospective studies, one randomized, have included DEXA assessment, but none has included CT or the LDCD score. The SMART Body Composition substudy provides an ideal setting in which to assess lipodystrophy severity over time: the study is a randomized, long-term study, and includes DEXA, CT, and subjective lipodystrophy assessments. Furthermore, about half the subjects reported lipodystrophy at baseline, so assessment of the score can be undertaken in adults with and without lipodystrophy. Inclusion of lipodystrophy prevalence and severity in this substudy will contribute to determining the overall benefits and risks of the DC and VS strategies of the SMART study.

Body composition is influenced by several factors. It is well known from established HIV and non-HIV literature that the level of physical activity influences lean body mass, fat mass and bone mineral density.^[171],^^[172],^^[173],^^[174]^ Also, the use of variety of drugs are known to change body composition. These include recombinant growth hormone, corticosteroids, thyroid supplements, anabolic steroids, drugs for osteoporosis, recombinant leptin, and PPAR gamma agonists.^[175],^^[176] ,^^[177],^^[178],^^[179],^^[180],^^[181]^ In addition, cosmetic procedures may directly influence the subjective and/or objective measures of body composition. These include liposuction, facial filling with polylactic acid, or silicone augmentation of buttocks. In this substudy, data on physical activity, concomitant medications and cosmetic procedures will be collected and used to help interpret differences in body composition between the VS and DC groups.

SMART is the only study where body composition can be studied with and without drug treatment for prolonged periods of time in both treatment-naïve and experienced patients. Moreover, due to their debilitating effect on body shape, the potentially increased risk of cardiac disease, and the deleterious effects on quality of life, the risks of these body composition changes may be a determining factor in selecting one versus another treatment strategy (i.e., viral suppression or drug conservation). Therefore it is imperative that a detailed and careful prospective evaluation of body composition changes in concert with metabolic, host and viral factors be undertaken in the SMART study. The SMART study offers a unique chance to assess the impact of cessation or deferred initiation versus continuation or initiation of antiretroviral therapy on body composition changes and metabolic abnormalities.

### 7.3.2. Objectives

**Primary Objectives**

1. To compare the DC group with the VS group for changes in limb fat, assessed by DEXA, averaged over follow up.
2. To compare the DC group with the VS group for changes in visceral fat, assessed by CT, averaged over follow up.

**Secondary Objectives**

1. To compare the DC group with the VS group for changes in regional and total body fat
2. To compare the DC group with the VS group for changes in regional and total bone mineral density
3. To compare the DC group with the VS group for changes in metabolic parameters
4. To compare the DC group with the VS group for lipodystrophy prevalence and severity
5. To compare the DC group with the VS group for body composition changes in subgroups defined by baseline host factors, (e.g., demographics, metabolic parameters), HIV disease stage, and duration of exposure to different drugs/drug classes.
6. To evaluate the change in metabolic parameters as predictors of changes in body composition.

### 7.3.3. Selected Outcome Measures

*Regional fat:* limb fat and truncal fat will be measured (in kilograms) by DEXA scan. Additionally, limb fat will be calculated as percentage of total limb mass. Visceral adipose tissue (VAT) and subcutaneous adipose tissue (SAT) will be measured (in cm^2^) by 3-cut CT scan (L2, mid and L4 level).

*Total fat* and *total bone mineral density* will be measured by DEXA scan.

*Regional bone mineral density* will be measured by CT scan of the spine, and by DEXA of the proximal femur and lumbar spine.

The *insulin resistance index* will be measured using the HOMA model.

*Lipodystrophy* prevalence and severity will be measured using the lipodystrophy case definition score^[182],^^[183]^.

### 7.3.4. Patient Selection

Data will be collected at selected CPCRA sites that have access to study-certified DEXA and CT scan equipment. A total of 300 patients will be enrolled

**Inclusion Criteria**

1. Signed informed consent for the Body Composition substudy
2. Valid baseline measurements can be obtained from each of the following scans:

- Total body DEXA (total body fat, limb fat and truncal fat)
- Spine DEXA (bone mineral density)
- CT at levels L2, L3 and L4 (SAT and VAT)
- QCT (bone mineral density)

### 7.3.5. Measurements

**Baseline Measurements:**

Within 60 days prior to randomization:

- Body composition measured by DEXA, qCT and 3-cut CT scan. For women of childbearing potential, a negative serum or urine pregnancy test result has to be obtained within 14 days prior to each of the scans.

Within 45 days prior to randomization:

- Fasting (12 hours minimum) serum sample for lipid and glycemic parameters (total cholesterol, LDL cholesterol, HDL cholesterol, VLDL cholesterol, triglycerides, serum glucose, insulin, and C-peptide)
- Fasting whole blood sample for hemoglobin A1c
- Fasting venous lactate
- Stored fasting serum sample for future metabolic research^[184]^
- Waist and hip circumference
- Body habitus assessment by the provider
- Self-reported physical exercise
- Self-reported history of cosmetic procedures
- Concomitant medications

**Follow-up Measurements:**

The following measurements will be collected at months 4, 8 and 12, and annually thereafter, unless otherwise noted:

- Fasting (12 hours minimum) serum sample for lipids, glucose, insulin, and C-peptide
- Fasting whole blood sample for hemoglobin A1c (annually)
- Fasting venous lactate (at months 4 and 12, and annually).
- Stored fasting serum sample for future metabolic research (annually)
- Body composition measured by DEXA, and 3-cut CT scan (annually). For women of childbearing potential, a negative serum or urine pregnancy test result has to be obtained within 14 days prior to each of the scans. While a patient is pregnant, DEXA and CT scans will not be performed.
- Waist and hip circumference (annually)
- Body habitus assessment by the provider (annually)
- Self-reported physical exercise (annually)
- Self-reported history of cosmetic procedures (annually)
- Concomitant medications (annually)

### 7.3.6. Subsampling Plan and Statistical Analysis

**Sample size**

The sample size is calculated to detect a difference in the average change in limb fat (from baseline throughout follow-up) of 0.5 kg with 80 % power and a significance level of 0.05 . The sample size is also sufficient to detect a difference in mean change in visceral fat from baseline of 25 cm^2^, and a difference of 1.0 kg in average total body fat.

Assuming a value of 1.4 kg for the standard deviation of the change in limb fat during follow-up, a total sample size of 250 patients (1:1 allocation) is needed to detect a difference in mean change of 0.5 kg with 80 % power and a significance level of 0.05. To allow for missing data and loss-to-follow-up, the sample size will be increased to n=300 patients.

There are no data on body composition changes in patients not receiving therapy.

The standard deviations used in the sample size calculations were derived from the PILR study which investigated the effect of discontinuing protease inhibitors. Data from n=73 patients in the PILR study provide the following observed standard deviations of *changes from baseline to week 24*: a) SD = 0.8 kg for the change in limb fat (measured by DEXA); b) SD=45 cm^2^ for the change in visceral fat (VAT; assessed by CT scan); c) SD=30 cm^2^ for the change in extra-abdominal fat (SAT; assessed by CT scan); and d) SD=1.9 kg for the change in total body fat (assessed by DEXA). Data from n=104 patients in the MITOX study^[185]^ provide similar values for standard deviations, a) SD=0.8 kg, b) SD=38 cm2, and d) SD=2.0 kg. The MITOX study investigated the effect of abacavir in AR experienced adults with lipoatrophy (n=111), over 24 weeks.

For sample size calculations, we inflated the standard deviation of changes in limb fat to 1.4 kg, to account for several factors that may result in higher standard deviations in the SMART study than observed in the PILR study : a) the within-patient variability in average change over 6-9 years may be higher than the change over 24 weeks observed in the PILR study; b) DEXA and CT scans will be performed at several different sites; and c) the 73 patients in the PILR study may not be representative of the patient population recruited for the SMART study (in particular, both the PILR and MITOX studies have too few female study participants to estimate the standard deviation for this subgroup reliably). The table below provides sample sizes necessary to detect a difference of 0.5 kg in mean change in limb fat with 80 % power and a significance level of 0.05.

Table 11

**Sample Size Required to Detect a Difference of 0.5 kg in Change in Limb Fat Between Two Treatment Groups with Power = 0.8 and Type 1 Error = 0.05**

| **SD of the Change in Limb Fat** | 1.0 kg | 1.2 kg | 1.4 kg | 1.6 kg |
| --- | --- | --- | --- | --- |
| **Sample size** | 128 | 184 | 250 | 324 |

The standard deviations of change in fat will be monitored during the study to ensure that adequate estimates were used in planning the sample size.

**Statistical Methods**

Analyses will be by intent-to-treat. Treatment groups will be compared using generalized linear models, adjusted for covariates such as age, gender, race, antiretroviral experience, and baseline clinical markers. Changes from baseline in total body fat, regional fat, VAT/SAT ratio, and bone mineral density will be compared between the VS and DC groups at month 12, and averaged over the total follow-up. Changes from baseline in metabolic and endocrine parameters will be compared between the VS and DC groups at month 4, month 8, month 12, averaged over the first 12 months, and averaged over the total follow-up. Changes from baseline in body habitus parameters (assessed by the provider) and lipodystrophy prevalence and severity (assessed by the lipodystrophy case definition scoreError: Reference source not found) will be compared between the VS and DC groups at fixed time points and, as appropriate, through time-to-event analyses. Additionally, changes over time in fat distribution will be compared using longitudinal regression methods. Regression analysis will be used to identify predictors of changes in body composition and to examine the relationships between changes in metabolic parameters and body composition. Information on cosmetic surgery, physical activity and concomitant medications through follow-up will be summarized, and used to help interpret differences in body composition between the VS and DC groups.

If sample size permits, the two treatment arms will be compared within subgroups of patients, defined by a) gender, and b) antiretroviral experience.

#### Appendix A. Hypothetical CD4+ Cell Count Patterns Over Follow-up

#### Appendix B. Sample Informed Consent

**DIVISION OF AIDS**

**COMMUNITY PROGRAMS FOR CLINICAL RESEARCH ON AIDS (CPCRA)**

*Protocol Title:*

**A LARGE, SIMPLE TRIAL COMPARING TWO STRATEGIES FOR MANAGEMENT**

**OF ANTI-RETROVIRAL THERAPY (CPCRA 065) (version 2.0)**

*Short Title of the Study:* **SMART Protocol (CPCRA 065) (version 2.0)**

INTRODUCTION

You are being asked to take part in this research study because you are infected with HIV, the virus that causes AIDS, and you have a CD4 count (T cell count) above 350 cells. This study is sponsored by the National Institutes of Health (NIH). The doctor in charge of this study at this site is *[insert name of Principal Investigator].* Before you decide if you want to be a part of this study, we want you to know about the study.

This is a consent form. It gives you information about this study. The study staff will talk with you about this information. You are free to ask questions about this study at any time. If you agree to take part in this study, you will be asked to sign this consent form. You will get a copy to keep.

WHY IS THE SMART STUDY BEING DONE?

The SMART study is being done because, although anti-HIV drugs are available, healthcare providers still have questions about the best ways to use these drugs to treat people living with HIV. Many healthcare providers now treat patients with daily drugs in order to keep the amount of virus in the blood (viral load) as low as possible. We know that this approach to using anti-HIV drugs is very effective in helping people with CD4 counts less than 200-250 cells live longer without serious diseases. But we do not know if this is the best way to use anti-HIV drugs to treat HIV disease in patients with higher CD4 counts over many years.

There is information suggesting that patients may be able to wait to use anti-HIV drugs because the risk of getting sick appears low while CD4 counts are higher than 250 cells. We are not sure if waiting to use anti-HIV drugs is better than using anti-HIV drugs at higher CD4 counts. Most information used to guide healthcare providers and patients about the treatment of HIV disease comes from different types of studies that were carried out over a short period of time.

A new kind of study that is carried out over several years is needed. To help healthcare providers and patients decide how to use anti-HIV treatments over many years, the SMART study will compare two ways of using anti-HIV drugs in a large number of HIV-infected people and follow them over a long period of time.

What is the SMART study about?

The SMART study will compare two ways of treating HIV disease over many years. Patients will be randomized (assigned by chance, like the flip of a coin) to one of two ways of using anti‑HIV drugs.

1. One way is to wait to use anti-HIV drugs while a patient’s chance of getting sick from HIV infection is low, based on his/her CD4 cell count. Patients treated in this way will be in the WAIT group. (Healthcare providers call this way the Drug Conservation or DC strategy.) Waiting until the CD4 count reaches 250 cells is not the usual way that HIV/AIDS is treated.

   This way of treating HIV may save anti-HIV drugs for use at times when your CD4 cell count is lower and may avoid drug side effects. Some of these side effects may be serious. The quality of your life may be improved. Also, this way could reduce difficulties associated with taking many different medicines, and costs. The risk of developing drug resistance may be lower due to the shorter time on anti-HIV drugs.
2. The second way is to use anti-HIV drugs at all times to keep the viral load (amount of virus in the blood) as low as possible. Patients treated in this way will be in the GO group. (Healthcare providers call this way the Viral Suppression or VS strategy.) Taking many drugs in different combinations for a long period of time is the usual way to treat HIV/AIDS.

   This way of treating HIV may prevent the virus from multiplying and prevent damage to your immune system. Also, this way could make the anti-HIV drugs work better because they are used when CD4 counts are higher. There may also be less chance of developing resistance to anti-HIV drugs since levels of virus in the blood stay lower.

We are not sure which of these ways is the better way to treat HIV. The study will compare these two ways of treating HIV disease to see if one way is better at lowering people's chance of getting sick/sicker and helps them live longer, compared with the other way.

HOW WILL THE STUDY WORK?

The study does not limit the types or number of anti-HIV drugs to be used. Your healthcare provider can prescribe any anti-HIV drugs for you. When to use anti-HIV drugs is guided by the study group that you are assigned to.

- If you are assigned to the WAIT group and are taking anti-HIV drugs, your healthcare provider will stop your anti-HIV drugs and will use them again only when your CD4 count drops to below 250 cells. If you are not on anti-HIV drugs, your provider will wait and start you on anti-HIV drugs if your CD4 count drops below 250 cells.

Once you are started on anti-HIV drugs, you will be treated with anti-HIV drugs until your CD4 count rises to at least 350 cells. At that point, your healthcare provider will advise you about stopping your anti-HIV drugs.

Your healthcare provider will monitor you carefully while you are off drugs. If you stop your anti-HIV drugs and your CD4 count drops again to below 250 cells, you will take anti-HIV drugs again. This cycle may be repeated as many times as your CD4 count rises and falls.

- If you are assigned to the GO group and are already taking anti-HIV drugs, you will continue taking anti-HIV drugs to keep your viral load as low as possible. If you are not taking anti-HIV drugs when you enter the study, you will start taking anti-HIV drugs right away.

If your viral load rises, your healthcare provider will change the anti-HIV drugs that you are taking.

WHAT DO I HAVE TO DO IF I AM IN THIS STUDY?

SCREENING

Within 45 days before randomization, your healthcare provider will examine you and ask you questions about your health and medicines you are taking. You will be asked about your education level. You will also be asked questions about any changes you may have noticed in your body appearance. If you are taking anti-HIV drugs, you will be asked about how you regularly take your anti-HIV drugs. It will take about 20 minutes to answer all these questions.

You will have approximately 3-4 tablespoons (45-60 milliliters [mL]) of blood drawn. Your blood will be used for routine tests, for tests to see if you have had viral hepatitis (a liver infection), and for tests of the levels of cholesterol and triglycerides (fats in the blood). Your blood will also be tested to see if your HIV virus is drug resistant (when the anti-HIV drugs no longer seem to work to slow or stop the virus from multiplying). The results of all these tests will be provided to you when available.

If you are a woman able to become pregnant, you must have a negative blood or urine pregnancy test within 14 days prior to randomization.

*Instruction to site personnel: Insert language in your informed consent ONLY if your site has access to the study-certified ECG and BIA equipment.*

*ECG*

- You will have an electrocardiogram (ECG or EKG), a painless, quick, and routine recording of the electrical activity of the heart. During the test, you will be asked to lie on a table. Ten electrodes will be placed on your body to record the electrical current coming from your heart. The results of this test will be provided to you when available.

*BIA*

- Your body composition will be measured using a quick and painless test called bioimpedance assay (BIA). During the test, you will be asked to lie on a table. Electrodes will be placed on your hand, wrist, foot, and ankle. A very weak current will pass through your body, and this measurement will be used to determine your body composition. Results of this test will be provided to you when available.

Optional Information at SCREENING

*Personal Information*

If you agree**,** you will be asked to give the research staff some personal identifying information (name, father’s last name, Social Security number, birth date, State of birth, State of residence). You may enroll in this study even if you decide not to provide this personal information. If you choose to provide the information, it may be used in the future to check the National Death Index, a computer information system listing the names of people who have died. This kind of check will be done if, at some point during the study, your healthcare provider has no other record of whether or not you are alive.

*Locator Information*

If you agree, you will be asked to provide the names of any friends or family members that the research staff may contact if they have problems getting in touch with you. If the information needed for this study is not available from your medical records, or if there are safety issues and you cannot be reached directly, your study healthcare provider may attempt to reach you by contacting those friends or family members. However, your healthcare provider will not release any confidential information about you.

Randomization

If you have agreed to take part in this study, you will be randomized to one of the two treatment groups – the WAIT group or the GO group. You have an equal chance of being assigned to either group.

You should come to the clinic or your healthcare provider's office at the time of randomization. If this is not possible, your healthcare provider will see you within the next 7 days to explain what you need to do while you are in the study and to get you started.

Followup Visits

Your healthcare provider will see you for study visits at months 1, 2, 4, 6, 8, 10, 12, and then every 4 months until the end of the study. Remember that your healthcare provider may want to see you more often if he/she thinks it is necessary. It is important to get to each appointment. You will be asked to keep these appointments even if you choose not to follow your assigned intervention.

*At most visits:*

- Your healthcare provider will ask you questions about your health, medications you are taking, and any symptoms you may have. If you are a woman able to become pregnant, your provider will ask you if you think you may be pregnant. Your healthcare provider may perform a pregnancy test (blood or urine), if necessary.
- You will have approximately 2-3 tablespoons (**30-45** mL) of blood drawn for routine tests and possible future HIV drug resistance testing. Results of the routine tests will be provided to you when available.
- You will fill out a questionnaire about taking your anti-HIV medications (if you are taking any). It will take about 10 minutes to fill it out.

*Once a year:*

- You will have about 1 tablespoon (15 mL) of blood drawn to check the levels of cholesterol and triglycerides (fats in the blood).
- You will complete a questionnaire about changes in your body appearance. It will take about 10 minutes to fill out the questionnaire.

*Instruction to site personnel: Insert language in your informed consent ONLY if your site has the study-certified ECG and BIA equipment.*

- You will have an ECG every 12 months.
- Your body composition will be measured with BIA every 12 months.

If You Move

If you move or transfer your medical care to another healthcare provider, the research staff would like to continue to collect information about your health for the main study and for any of the smaller questions (substudies) that you may be taking part in. Information about the SMART substudies is found later in this consent form.

With your permission, your study healthcare provider will contact your new healthcare provider and ask him or her to provide information about your health. The types of information your new provider will be asked for are routine primary care information, such as results of laboratory tests (for example, CD4 cell counts, viral load, and routine blood work), what medications you are taking, and any new diagnoses. When you move, you will be asked to sign a "Release of Medical Information" form for your new healthcare provider. A separate signature sheet will be given to you to sign if you agree to allow the collection of information about you if you move away or change primary care providers.

HOW MANY PEOPLE WILL TAKE PART IN THIS STUDY?

In all, the plan is to enroll about 6,000 people in the SMART study. **The first 1,000 people enrolled were in the "pilot phase" of the study to see if it was safe and could be finished. This “pilot phase” has been completed. A Data and Safety Monitoring Board (DSMB) has reviewed the study data and had no concerns about safety, so the study will continue. (A DSMB is an outside group of experts who monitor the study.)**

HOW LONG WILL I BE IN THIS STUDY?

You will be in this study up to 9 years, depending on when you join.

WHY WOULD MY HEALTHCARE PROVIDER TAKE ME OFF THIS STUDY EARLY?

Your healthcare provider may need to take you off the study early without your permission if:

- The National Institutes of Health (NIH) or the Institutional Review Board (IRB) cancels the study. (An IRB is a committee that watches over the safety and rights of research subjects.)
- A Data and Safety Monitoring Board (DSMB) recommends that the study be stopped early. The DSMB will review the safety data and other information for the SMART study at least once a year. They could decide to stop the study early.

WHAT ARE THE RISKS OF THE STUDY?

Risks of Each Way of Treating HIV in the Study

Each way of treating HIV disease in this study may be associated with potential benefits and certain risks. It is not known in the long run which of these strategies will be less risky.

WAIT group: The long-term risks of stopping anti-HIV drugs are not clear. People who stop anti-HIV drugs will probably have a drop in their CD4 cell counts and a rise in viral loads compared to those who continue taking them as in the GO group. It is expected, but not certain, that the CD4 cell count will go back up and the viral load back down, after re-starting anti-HIV drugs. These people may also have a greater chance of developing opportunistic infections if the CD4 cell count drops too much compared to patients in the GO group. Patients with higher viral loads may be able to pass the virus to others. It is possible that having a higher viral load and stopping or starting HIV drugs may lead to HIV resistance.

GO group: The long-term risks of ongoing use of anti-HIV drugs are not clear. People who take anti-HIV drugs over many years will probably have more side effects than patients who take anti-HIV drugs for shorter periods of time as in the WAIT group. Also, patients may find it hard to take anti-HIV drugs according to their healthcare providers' directions for many years, which may lead to the development of HIV drug resistance. It is also possible that using more anti-HIV drugs and staying on them for longer periods may lead to the development of HIV resistance. You may have fewer drugs options available when the risk of disease is high as compared to patients in the WAIT group.

Risks of Anti-HIV Treatment

All anti-HIV drugs can cause side effects. Your healthcare provider will discuss with you the risks of the specific anti-HIV drugs that you take. These risks are not specific to this study but would be associated with taking these drugs, whether you are on the study or not. It is also possible for the HIV virus to develop resistance to any anti-HIV drug. It is not known which way of treating HIV disease in this study will lead to resistance to the most drugs over time.

Risks of Drug Interaction

For your safety, you must tell your healthcare provider about all medicines, including prescription, over-the-counter (non-prescription), and herbal or alternative medicines, you are taking. This is because there may be serious side effects when other medicines are taken with anti-HIV drugs. Also, please let your healthcare provider know before you enroll in any other studies while on this study.

Risks of Blood Collection

The risks of having blood taken for testing may include pain, inflammation (swelling that is generally painful, red, and warm), and a bruise at the puncture site.

ARE THERE RISKS RELATED TO PREGNANCY?

Some anti-HIV drugs may cause harm to an unborn baby. Also, if you are not taking anti-HIV drugs, the risk that HIV infection may be passed to your unborn baby could be increased. The risks to the unborn baby from the anti-HIV drugs taken by men fathering children are unknown but could be serious.

The study staff will talk to you about choosing reliable birth control that you must use during the study and for 6 weeks after you stop the study.

*For women:* If you think you may want to become pregnant at any time during this study, tell your healthcare provider right away. If you become pregnant, your anti-HIV drugs may need to be changed; or, if you are not taking any anti-HIV drugs, you may be started on them. The study staff will discuss the different medicines and choices with you. You will be allowed to stay in the study. If you choose to stay in the study, you will be asked to sign another consent form.

ARE THERE BENEFITS TO TAKING PART IN THIS STUDY?

If you take part in this study, there may be a direct benefit to you, but no guarantee can be made. You will receive results of some tests that are not part of the standard of care. It is also possible that you may receive no benefit from being in this study. Information learned from this study may help others who have HIV.

WHAT OTHER CHOICES DO I HAVE BESIDES THIS STUDY?

Instead of being in this study you have the choice of:

- HIV treatment using current standards and guidelines
- No treatment

Please talk to your healthcare provider about these and other choices available to you.

WHAT ABOUT CONFIDENTIALITY?

**We will do everything we can to protect your privacy. In addition to the efforts of the study staff to help keep your personal information private, we have gotten a Certificate of Confidentiality from the U.S. Federal Government. This certificate means that researchers cannot be forced to tell people who are not connected with this study, such as the court system, about your participation. Also, any publication of this study will not use your name or identify you personally.**

**People who may review your records include: the (insert Name of Site) IRB, National Institutes of Health (NIH), study coordinating center, study staff, study monitors, and their designees. Having a Certificate of Confidentiality does not prevent you from releasing information about yourself and your participation in the study.**

WHAT ARE THE COSTS TO ME?

Procedures and tests that are required for the study but are not usually part of standard care will be paid for by the study. You, your insurance company, or other third-party payer will have to pay for clinic visits, laboratory tests, and medicines.

WHAT HAPPENS IF I AM INJURED?

If you are injured as a result of being in this study, you will be given immediate treatment for your injuries. The cost for this treatment will be charged to you or your insurance company. There is no program for compensation either through this institution or the National Institutes of Health (NIH). You will not be giving up any of your legal rights by signing this consent form.

WHAT ARE MY RIGHTS AS A RESEARCH SUBJECT?

Taking part in this study is completely voluntary. You may choose not to take part in this study or leave this study at any time. You will be treated the same no matter what you decide.

We will tell you about new information from this or other studies that may affect your health, welfare, or willingness to stay in this study. When the study is completed, we will tell you the results of it.

WHAT DO I DO IF I HAVE QUESTIONS OR PROBLEMS?

For questions about this study or a research-related injury, contact:

- *[name of the investigator or other study staff]*
- *[telephone number of above]*

For questions about your rights as a research subject, contact:

- *[name or title of person on the Institutional Review Board (IRB) or other organization appropriate for the site]*
- *[telephone number of above]*

SUBSTUDIES

*Instruction to site personnel: Insert language in your informed consent ONLY if your site is participating in the substudy data collection.*

If you choose to participate in a substudy, your signature will be required. You do not have to participate in a substudy to be eligible to participate in the main study. You may later quit any substudy you participate in and stay in the main study without penalty and without affecting your medical care.

**Quality of Life and Healthcare Utilization Substudy (CPCRA 065A)**

This substudy compares the WAIT and GO groups for use of healthcare services and quality of life. Information from the substudy will be help healthcare providers get a better picture of how the two ways of treating HIV disease compare for cost and for quality of life. About **1200** patients will enroll into this substudy.

At baseline you will be asked questions about your use of health care services, your income, your health insurance, and your quality of life. It will take about 15 minutes to finish these questionnaires.

At months 4, 8, 12, then every year, you will fill out a questionnaire about your quality of life. It will take about 10 minutes to finish this questionnaire.

Every year you will be asked questions about your use of health care services, your household income, and your health insurance. It will take about 10 minutes to finish this questionnaire.

**HIV Transmission Risk Behavior Substudy (CPCRA 065B)**

This substudy looks at behavior that may be associated with HIV transmission (passing the virus to someone else), such as sexual behavior and injecting drug use. This substudy will try to find out whether there are important differences between the WAIT and GO groups related to these types of behavior. About 600 patients will enroll into this substudy.

At baseline, months 4 and 12, then every year:

- You will fill out a questionnaire about your behavior that may be associated with HIV transmission, such as sexual behavior and injecting drug use. It will take about 10 minutes to answer these questions.
- You will provide a urine sample for testing for common sexually transmitted diseases (gonorrhea and chlamydia). You will be given the results when they are available.
- You will provide 1 tablespoon (15 mL) of blood to test for syphilis and HHV-8, two sexually transmitted infections. You will be given the results of the test for syphilis when they are available.

If you are found to have a sexually transmitted infection, based on tests in this substudy, you will be counseled about how to protect yourself from getting such an infection again. You will also be treated according to current guidelines.

**SIGNATURE PAGE**

If you have read this consent form (or had it explained to you), all your questions have been answered and you agree to take part in this study (CPCRA 065), please sign your name below.

| Participant’s Name (print) |  | Participant’s Signature and Date |
| --- | --- | --- |
| Participant’s Legal Guardian (print) (As appropriate) |  | Legal Guardian’s Signature and Date |
| Study Staff Conducting  Consent Discussion (print) |  | Study Staff Signature and Date |
| Witness’ Name (print)  (As appropriate) |  | Witness’ Signature and Date |

SIGNATURE PAGE
for THE QUALITY OF LIFE AND HEALTHCARE UTILIZATION SUBSTUDY (CPCRA 065A)

If you voluntarily agree to join the Quality of Life and Healthcare Utilization Substudy, please sign your name below:

| Participant’s Name (print) |  | Participant’s Signature and Date |
| --- | --- | --- |
| Participant’s Legal Guardian (print) (As appropriate) |  | Legal Guardian’s Signature and Date |
| Study Staff Conducting  Consent Discussion (print) |  | Study Staff Signature and Date |
| Witness’ Name (print)  (As appropriate) |  | Witness’ Signature and Date |

SIGNATURE PAGE
for THE HIV Transmission Risk Behavior Substudy (CPCRA 065B)

If you voluntarily agree to join the HIV Transmission Risk Behavior Substudy, please sign your name below:

| Participant’s Name (print) |  | Participant’s Signature and Date |
| --- | --- | --- |
| Participant’s Legal Guardian (print) (As appropriate) |  | Legal Guardian’s Signature and Date |
| Study Staff Conducting  Consent Discussion (print) |  | Study Staff Signature and Date |
| Witness’ Name (print)  (As appropriate) |  | Witness’ Signature and Date |

**Optional Personal Information: If you agree to provide personal identifying information (name, father's last name, Social Security number, birth date, State of birth, State of residence) for confidential study use, please sign your name below.**

| Participant’s Name (print) |  | Participant’s Signature and Date |
| --- | --- | --- |
| Participant’s Legal Guardian (print) (As appropriate) |  | Legal Guardian’s Signature and Date |
| Study Staff Conducting  Consent Discussion (print) |  | Study Staff Signature and Date |
| Witness’s Name (print)  (As appropriate) |  | Witness’s Signature and Date |

**Optional Locator Information: If you agree to provide the names of any friends or family members that may be contacted for study information if there are problems getting in touch with you (your provider will not release any confidential information about you), please sign your name below.**

| Participant’s Name (print) |  | Participant’s Signature and Date |
| --- | --- | --- |
| Participant’s Legal Guardian (print) (As appropriate) |  | Legal Guardian’s Signature and Date |
| Study Staff Conducting  Consent Discussion (print) |  | Study Staff Signature and Date |
| Witness’ Name (print)  (As appropriate) |  | Witness’ Signature and Date |

Body Composition Substudy (CPCRA 065C)

If you decide not to take part in this substudy, you may still participate in the main SMART study. If you choose to participate in this substudy, your signature will be required. You may later quit the substudy and stay in the main study without penalty and without affecting your medical care.

**WHY IS THE BODY COMPOSITION SUBSTUDY BEING DONE?**

The Body Composition substudy will compare the two ways of treating HIV disease (WAIT and GO groups) for how they affect changes in body composition and in some blood measures over many years.

WHAT DO I HAVE TO DO IF I AM IN THIS SUBSTUDY?

SCREENING

***Within 60 days of randomization into the main study:***

- You will have a CT scan, which takes about *** minutes *[sites to insert amount of time appropriate for your site/procedure].* A CT (computed tomography) scan is a special kind of x-ray that takes pictures of the body using a small amount of radiation. You will be asked to lie quietly while the scan is taken. Some people require medication to help them relax during this test. Your CT scan results will be provided to you when available.
- For women of childbearing potential, a negative serum or urine pregnancy test is required if there is suspicion that you may be pregnant, before either the DEXA and the CT scan. This test must be taken within 14 days prior to the scan. Additionally, your scan center may require confirmation of a pregnancy test prior to the DEXA or CT scan.
- You will also have a DEXA scan. A DEXA (dual-energy x-ray absorptiometry) scan is a special kind of x-ray using a small amount of radiation. It allows the healthcare provider to see body parts or layers better than a regular x-ray. During the DEXA scan, you will lie very still on a table for about *** minutes *[sites to insert amount of time appropriate for your site/procedure]*, while a machine passes above your entire body. The machine will create a picture (scan) of the bones and fat in your body. Your DEXA scan results will be provided to you when available.

*Within 45 days of randomization into the main study:*

- You will have approximately 3 tablespoons (45 mL) of blood drawn to check the levels of glucose (sugar), insulin (a hormone), cholesterol, and triglycerides (fats); and to have a special test, venous lactate, which measures how your body cells function. **Other tests of your metabolism will also be done on your blood**. You will fast (take nothing by mouth except water or medicine) for at least 12 hours before blood is drawn at this visit. And your healthcare provider will give you special instructions about your activity level and talk to you about how to take your medications on this day. You will be given the results of this testing.
- You will have about **2** tablespoons (30 mL) of blood drawn for future metabolic tests. You will be given the results of these tests when they are available.
- Your healthcare provider will assess possible changes in your body appearance.
- **You will be asked questions about medications you are taking.**
- **Your waist and hip will be measured with a tape measure.**
- **You will be asked about how much you exercise and about any cosmetic surgery you have had.**

Followup Visits

You will return for regular visits at months 4, 8, 12, and every 12 months after that.

*At all of these visits*:

You will have approximately 1 tablespoon (15 mL) of blood drawn to check the levels of glucose (sugar), insulin (a hormone), cholesterol, and triglycerides (fats). You will fast for at least 12 hours before blood is drawn at this visit. You will be given the result of this testing when it is available.

*At some of these visits:*

You will have approximately 1 tablespoon (15 mL) of blood drawn for the venous lactate test. You will be given the results of this test when they are available.

*Every year:*

You will have a DEXA scan and a CT scan every year. The results will be available to you through your study healthcare provider. You will also have about 2 tablespoons (30 mL) of blood drawn for future metabolic tests. You will be given the results of these tests when they are available.

- **You will be asked questions about medications you are taking.**
- **Your waist and hip will be measured with a tape measure.**
- **You will be asked about how much you exercise and about any cosmetic surgery you have had.**

HOW MANY PEOPLE WILL TAKE PART IN THIS SUBSTUDY?

300 people will take part in this substudy.

HOW LONG WILL I BE IN THIS SUBSTUDY?

You will be in this substudy up to 9 years, depending on when you join.

WHAT ARE THE RISKS OF THE SUBSTUDY?

Risks of DEXA and CT Scans

- The only risk of a DEXA or CT scan is radiation, the same risk as all other x-ray tests. Exposure to x-rays will be at a level that is far below the normal amount of radiation in the environment that people are exposed to. If, after taking the DEXA or CT scan, you choose not to participate in the study, you will have been unnecessarily exposed to radiation.
- Some people may feel discomfort lying still and flat on a table.

**ARE THERE BENEFITS TO TAKING PART IN THIS SUBSTUDY?**

If you take part in this substudy, there may be a direct benefit to you, but no guarantee can be made. You will receive results of tests that are not part of the standard of care. It is also possible that you may receive no benefit from being in this study. Information learned from this study may help others who have HIV.

**SIGNATURE PAGE
FOR THE BODY COMPOSITION SUBSTUDY (CPCRA 065C)**

All other information that is contained in the main study consent form that you signed applies to this substudy consent form as well.

If you have read this consent form (or had it explained to you), all your questions have been answered, and you agree to take part in this substudy, please sign your name below.

| Participant’s Name (print) |  | Participant’s Signature and Date |
| --- | --- | --- |
| Participant’s Legal Guardian (print) (As appropriate) |  | Legal Guardian’s Signature and Date |
| Study Staff Conducting  Consent Discussion (print) |  | Study Staff Signature and Date |
| Witness’ Name (print)  (As appropriate) |  | Witness’ Signature and Date |

**PERMISSION TO MAINTAIN CONTACT AND CONTINUE FOLLOWUP**

**IN THE SMART STUDY IF YOU CHANGE HEALTHCARE PROVIDERS**

(To be signed when a move is anticipated)

If you move or transfer your medical care to another healthcare provider and you voluntarily agree to allow us to contact your new healthcare provider at study visit times and ask him/her to provide us with information about your health, please sign below.

| Participant’s Name (print) |  | Participant’s Signature and Date |
| --- | --- | --- |
| Participant’s Legal Guardian (print) (As appropriate) |  | Legal Guardian’s Signature and Date |
| Study Staff Conducting  Consent Discussion (print) |  | Study Staff Signature and Date |
| Witness’s Name (print)  (As appropriate) |  | Witness’s Signature and Date |

#### Appendix C. Sample Informed Consent for the Storage of Specimens

**INFORMED CONSENT**

**FOR THE STORAGE OF SPECIMENS OBTAINED WHILE PARTICIPATING
IN A NIH-SPONSORED CPCRA RESEARCH TRIAL**

*Protocol Title:*

**A LARGE, SIMPLE TRIAL COMPARING TWO STRATEGIES FOR MANAGEMENT OF ANTI-RETROVIRAL THERAPY (CPCRA 065) (version 2.0)**

*Short Title for the Study:* **SMART Study (CPCRA 065) (version 2.0)**

**INTRODUCTION**

You have decided to take part in a research study being sponsored by the Division of AIDS. While you are in this research study, there may be some samples of blood taken from you that might be useful for future research. You are being asked to agree to the storage of these samples and to their use for future research.

This consent form gives you information about the collection, storage, and use of your samples. The study staff will talk with you about this information. Please ask if you have any questions. If you agree to the storage of your samples for future research, you will be asked to sign this consent form. You will get a copy to keep.

**HOW WILL YOU GET THE SAMPLES FROM ME?**

If you agree to allow the researchers to take additional samples for storage, you will have about **3 tablespoons (45 mL)** of blood drawn at baseline and each 12-month study visit. These additional samples and any leftover samples will be kept and used for future research.

**HOW WILL YOU USE MY SAMPLES?**

Your samples will be used only to learn more about HIV infection and its complications. The research may include studies to understand how HIV causes disease and complications and how to best treat or prevent HIV infection and its complications. Samples may also be used to study other problems that are very important to persons with HIV infection, such as liver disease, diabetes, or heart disease. Testing may include studies of HIV, studies of other infections that affect people with HIV (for example, hepatitis viruses), studies of your cells, proteins, and other chemicals in your body.

The researchers do not plan to contact you or your healthcare provider with any results from these studies done on your stored samples. This is because research tests are often done with experimental procedures; and, in general, results from only one research study should not be used to make a decision on how to treat your disease.

Your samples will not be sold or used directly to produce commercial products. Research studies using your samples will be reviewed by the National Institutes of Health and a special committee at the researcher’s institution (an Institutional Review Board).

**WILL MY SAMPLES BE USED FOR STUDIES OF MY GENES (DNA)?**

You will not be asked, at this time, to allow studies of your genes (DNA) on your stored blood samples. If, in the future, the CPCRA wants to do a test of your genes, you will be asked, at that time, if you will allow the testing.

**HOW LONG WILL YOU KEEP MY SAMPLES?**

There is no time limit on how long your samples will be stored.

**HOW WILL MY SAMPLES BE STORED?**

Your samples will be stored at special facilities that are designed to store samples safely and securely. The storage facilities are designed so that only researchers approved by CPCRA and NIH will have access to the samples. Some employees of the storage facilities will need to have some access to your samples in order to store them and to keep track of where they are, but these people will not have information that directly identifies you. An Institutional Review Board will oversee the storage facilities to protect you and other research volunteers from harm.

**DOES STORAGE OF MY SAMPLES BENEFIT ME?**

There are no direct benefits to you. The benefit of doing research on stored samples includes learning more about HIV infection and its complications in order to help people who have HIV.

**WHAT ARE THE RISKS?**

There are no risks to your health related to storing your samples. Possible risks to your privacy are described in the next section.

**WHAT ABOUT CONFIDENTIALITY?**

In order to keep your information private, your samples will be labeled with a code that can be traced back only to your research clinic. Your personal information (name, address, phone number) will be protected by the research clinic. When researchers are given your stored samples to study, they will not be given your personal information. The results of future tests will not be included in your health records unless you are notified of a special test result and you ask that your test result be sent to another healthcare provider. Every effort will be made to keep your personal information confidential.

**WHAT ARE MY RIGHTS?**

Allowing your samples to be stored is voluntary. You may decide not to have samples stored and still be in this research study or any future study.

If you decide now that your samples can be stored for future research, you may change your mind at any time. You must contact your study provider or nurse and let him/her know that you do not want your samples used for future research. Your samples will then no longer be used.

**WHAT DO I DO IF I HAVE QUESTIONS?**

For questions about the storage of your samples, contact *[insert the name of the investigator]* at *[insert telephone number].*

For questions about your rights related to the storage of your samples for research, contact *[insert the name or title of person on the Institutional Review Board]* at *[insert telephone number].*

**SIGNATURE PAGE:**

**CONSENT FOR THE STORAGE OF SPECIMENS OBTAINED WHILE PARTICIPATING IN A NIH-SPONSORED CPCRA RESEARCH TRIAL**

Please carefully read the statements below and think about your choice. No matter what you decide, it will not affect your health care or your participation in this study.

I agree to have any of my leftover **blood** samples stored and used for future research related to HIV infection and its complications. I understand that this agreement does not apply to tests of my genes (DNA).

_____Yes

_____No

I agree to have additional **blood** samples **(1 tablespoon [15mL])** taken **at baseline and each 12-month study visit** for the purpose of storage and to be used for future research related to HIV infection and its complications. I understand that this agreement does not apply to tests of my genes (DNA).

_____Yes

_____No

**For sites that are able to ship samples for PBMC processing, the following text should be included in the IC submitted to the protocol registration office.**

**Some sites participating in the main study will also collect blood specimens that require special shipping and processing.  This blood will be separated into cells and plasma before being frozen and stored for future research.**

**I understand that I do not have to agree to this extra blood collection in order to participate in the main study.**

**I agree to have another additional blood sample (2 tablespoons [30mL]) taken at baseline and each 12-month study visit for storage and future research related to HIV infection and its complications.  I understand that this agreement does not apply to tests of my genes (DNA).**

**_____Yes**

**_____No**

| Participant’s Name (print) |  | Participant’s Signature and Date |
| --- | --- | --- |
| Participant’s Legal Guardian (print) (As appropriate) |  | Legal Guardian’s Signature and Date |
| Study Staff Conducting  Consent Discussion (print) |  | Study Staff Signature and Date |
| Witness’ Name (print)  (As appropriate) |  | Witness’ Signature and Date |

#### Appendix D. Sample Informed Consent for Women who Become Pregnant

**INFORMED CONSENT FOR WOMEN WHO BECOME PREGNANT WHILE PARTICIPATING IN THE SMART STUDY**

*Protocol Title:*

A Large, Simple Trial Comparing Two Strategies for Management of Anti-Retroviral Therapy (CPCRA 065)

(version 2.0)

*Short Title of the Study:* **SMART Protocol (CPCRA 065) (Version 2.0)**

INTRODUCTION

You are being asked to sign this informed consent because you became pregnant while participating in the research study called the SMART study. This study was designed so that women who were pregnant could not join the study. You have already signed an informed consent for the study. The first consent you signed still applies to your participation in the study.

Since you are pregnant, this new consent form will give you additional information related to your pregnancy and your baby and how it may affect the treatments you are receiving as part of the SMART study. You need to sign this consent if you agree to continue participation in the study. You will be given a copy to keep. You are free to ask questions of the study staff at any time.

WHAT DO I HAVE TO DO IF I STAY IN THIS STUDY?

If you decide to stay in this study while you are pregnant, you will continue to come to the clinic for study visits as described in the section "What Do I Have to Do if I Am in This Study?" of the first consent you signed.

If you are taking anti-HIV drugs, your healthcare provider may stop or change some of them. If you are not taking anti-HIV drugs, your healthcare provider will start you on them. You will be advised to take anti-HIV drugs according to current guidelines for treating pregnant women and for lowering the chance of passing the HIV virus to the baby. Your healthcare provider will discuss with you the risks and benefits to you and your baby of the use of anti-HIV drugs during your pregnancy. You will not have DEXA and CT scans done while you are pregnant.

This study will not provide care related to your pregnancy, the delivery of your baby, or the care of your baby. You must arrange for your care and your baby’s care outside of this study. Your healthcare provider will assist you in referring you to another health care provider who specializes in pregnancy and delivery. You will also be advised to have your baby cared for by a pediatrician. The healthcare provider who usually takes care of you in the clinic will continue to provide medical care for you.

WHAT ARE THE RISKS RELATED TO STAYING IN THE STUDY?

Now that you are pregnant, there are some possible risks you should know. These possible risks to you and your baby are in addition to the risks that are described in the study consent you already signed. You should reread the section "Are There Risks Related to Pregnancy?" in the first consent you signed. Taking anti-HIV medications may be associated with additional or more severe side effects in pregnancy. Your provider will need to monitor you carefully during pregnancy. In addition, specific anti-HIV medications should be avoided during pregnancy.

If you are assigned to the viral suppression group (VS or GO Group) of the SMART study and are on anti-HIV medications, your provider may continue you on the same treatment or may modify the treatment.

If you are assigned to the drug conservation group (DC or WAIT Group) of the SMART study and are not taking anti-HIV medications, your provider will need to start you on them. Your provider will discuss with you the risks and benefits of taking these medications.

The risks to the baby with the use of anti-HIV medications are not completely understood. The use of anti-HIV medications has been shown to decrease the chance of the baby's getting HIV, but it is unknown if anti-HIV medications could cause illness or birth defects in the baby.

BREASTFEEDING

Current recommendations indicate that HIV-infected women should avoid breastfeeding. You should reread the information on risks listed in the main informed consent.

ARE THERE BENEFITS TO STAYING IN THIS STUDY?

If you continue to take part in this study, there may be a benefit to you and your baby, but no guarantee can be made. It is also possible that you and your baby will receive no benefit from continuing in this study. Information learned from this study may help others who have HIV.

WHAT OTHER CHOICES DO I HAVE BESIDES STAYING IN THE
SMART STUDY?

If you decide not to continue in the SMART study during your pregnancy, you will continue to receive care and will not lose your medical benefits. Your healthcare provider will discuss with you the treatment choices available to you and the risks and benefits of these choices.

WHAT WILL I DO AFTER PREGNANCY?

When you are no longer pregnant, your participation in the SMART study will continue. Your treatment will be according to your originally assigned group.

WHAT ABOUT CONFIDENTIALITY?

Efforts will be made to keep your personal information confidential. We cannot guarantee absolute confidentiality. Your personal information may be disclosed if required by law. Any publication of this study will not use your name or identify you personally.

Your records may be reviewed by the *[insert name of site]* IRB, National Institutes of Health (NIH), **study coordinating center**, study staff, and study monitors.

WHAT ARE THE COSTS TO ME?

In addition to any costs that are described in the study consent you already signed; this study will not cover any cost related to your pregnancy, delivery of your baby, or care of your baby.

WHAT HAPPENS IF MY BABY OR I AM INJURED?

If your baby or you are injured as a result of being in this study, you will both be given immediate treatment for your injuries. The cost for this treatment will be charged to you or your insurance company. There is no program for compensation, either through this institution or the National Institutes of Health (NIH). You will not be giving up any of your legal rights by signing this consent form.

WHAT ARE MY RIGHTS AS A RESEARCH SUBJECT?

Continuing to take part in this study is completely voluntary. You may choose not to continue in this study or leave this study at any time. You will be treated the same no matter what you decide.

We will tell you about new information from this or other studies that may affect your health, welfare, or willingness to stay in this study. If you want the results of the study, once available, let the study staff know.

WHAT DO I DO IF I HAVE QUESTIONS OR PROBLEMS?

For questions about this study or a research-related injury, contact:

- *[site insert name of the investigator or other study staff]*
- *[site insert telephone number of above]*

For questions about your rights as a research subject, contact:

- *[site insert name or title of person on the Institutional Review Board (IRB) or other organization appropriate for the site]*
- *[site insert telephone number of above]*

**SIGNATURE PAGE**

If you have read this consent form (or had it explained to you), all your questions have been answered, and you agree to take part in this study, please sign your name below.

| Participant’s Name (print) |  | Participant’s Signature and Date |
| --- | --- | --- |
| Participant’s Legal Guardian (print) (As appropriate) |  | Legal Guardian’s Signature and Date |
| Study Staff Conducting  Consent Discussion (print) |  | Study Staff Signature and Date |
| Witness’ Name (print)  (As appropriate) |  | Witness’ Signature and Date |

#### Appendix E. Time-and-Events Schedule

| Requirement | Screening visit (within 45 days before  randomization) | Randomization | | Follow-up visits Year 1  (Months) | | | | | | | Follow-up visits  Years 2-9 (Every 4 months) | | |
| --- | --- | --- | --- | --- | --- | --- | --- | --- | --- | --- | --- | --- | --- |
|  |  | Within 14 | At or within |  |  |  |  |  |  |  |  |  |  |
|  |  | days before | 7days after | 1 | 2 | 4 | 6 | 8 | 10 | 12 | 4 | 8 | 12 |
| Study visit: Explain requirements of assigned treatment strategy (DC or VS), and initiate intervention |  |  | X |  |  |  |  |  |  |  |  |  |  |
| Targeted health history (selected diagnoses and treatments) | X |  |  |  | X | X | X | X | X | X | X | X | X |
| AR treatment history | X |  |  |  |  |  |  |  |  |  |  |  |  |
| CD4+ cell count and CD4 % (determined locally) | X |  |  | X | X | X | X | X | X | X | X | X | X |
| Nadir CD4+ cell count available in the medical record and nadir CD4+ cell count by patient self-report | X |  |  |  |  |  |  |  |  |  |  |  |  |
| Plasma for HIV RNA level (determined locally) | X |  |  | X | X | X | X | X | X | X | X | X | X |
| Up to 3 most recent *additional* CD4+ cell counts, CD4 %, and HIV RNA measurements available in the medical record | X |  |  |  |  |  |  |  |  |  |  |  |  |
| Interim CD4+ cell counts, CD4 %, and HIV RNA measurements obtained since last visit |  |  |  | X | X | X | X | X | X | X | X | X | X |
| Documentation by laboratory report of hepatitis C antibody serostatus^[186]^ and hepatitis B serostatus**^[187]^** | X |  |  |  |  |  |  |  |  |  |  |  |  |
| Serum sample (fasting or non-fasting)**^[188]^** to determine: total cholesterol, LDL, HDL, VLDL cholesterol, triglycerides | X |  |  |  |  |  |  |  |  | X |  |  | X |
| Stored sample of PBMCs that are viably cryopreserved for future virologic and immunologic research^[189]^ | X |  |  |  |  |  |  |  |  | X |  |  | X |
| Stored plasma samples for future HIV-related, CPCRA-approved research (requires additional consent) | X |  |  |  |  |  |  |  |  | X |  |  | X |
| Stored serum samples for future HIV-related, CPCRA-approved research (requires additional consent) | X |  |  |  |  |  |  |  |  | X |  |  | X |
| Plasma specimen for genotypic resistance testing | X |  |  |  |  |  |  |  |  |  |  |  |  |
| Stored plasma sample for future HIV drug resistance testing | X |  |  | X^[190]^ | X^2^ | X^2^ | X^2^ | X^2^ | X^2^ | X^2^ | X^2^ | X^2^ | X^2^ |
| Concomitant Medications | X |  |  |  |  |  |  |  |  | X |  |  | X |
| Smoking status | X |  |  |  |  |  |  |  |  | X |  |  | X |
| Personal identifying information for ascertaining survival status (requires additional consent) | X |  |  |  |  |  |  |  |  |  |  |  |  |
| For women of childbearing potential, documented negative pregnancy test result on a specimen (serum or urine) |  | X |  |  |  |  |  |  |  |  |  |  |  |
| For women of child-bearing potential, self-reported pregnancy status |  |  |  |  | X | X | X | X | X | X | X | X | X |
| Current AR therapy and changes since last visit |  |  |  |  | X | X | X | X | X | X | X | X | X |
| Changes in body appearance (patient self-report) | X |  |  |  |  |  |  |  |  | X |  |  | X |
| Self-reported adherence to antiretroviral therapy (for patients who are currently prescribed AR therapy) | X |  |  |  |  | X |  | X |  | X | X | X | X |
| Demographic data**^[191]^** | X |  |  |  |  |  |  |  |  |  |  |  |  |
| ECG**^[192]^** | X |  |  |  |  |  |  |  |  | X |  |  | X |
| BIA^[193]^ | X |  |  |  |  |  |  |  |  | X |  |  | X |

**Time-and-Events Schedule:
 Additional data collection in substudies**

| Requirement | Screening visit (within 45 days before  randomization) | Randomization | | Follow-up visits Year 1  (Months) | | | | | | Follow-up visits  Years 2-9 (Every 4 months) | | |
| --- | --- | --- | --- | --- | --- | --- | --- | --- | --- | --- | --- | --- |
|  |  | Within 14 | At or within |  |  |  |  |  |  |  |  |  |
|  |  | days before | 7 days after | 2 | 4 | 6 | 8 | 10 | 12 | 4 | 8 | 12 |
| **Quality of life and healthcare utilization substudy (n=1,200)** |  |  |  |  |  |  |  |  |  |  |  |  |
| Self-reported healthcare utilization | X |  |  |  |  |  |  |  | X |  |  | X |
| Demographic data**^[194]^** | X |  |  |  |  |  |  |  | X |  |  | X |
| Self-reported quality of life | X |  |  |  | X |  | X |  | X |  |  | X |
| Symptom severity | X |  |  |  | X |  | X |  | X |  |  | X |
|  |  |  |  |  |  |  |  |  |  |  |  |  |
| **HIV transmission risk substudy (n=600)** |  |  |  |  |  |  |  |  |  |  |  |  |
| Self-reported HIV transmission risk behavior | X |  |  |  | X |  |  |  | X |  |  | X |
| Urine testing**^[195]^** for *Neisseria gonorrhea* and *Chlamydia trachomatis* | X |  |  |  | X |  |  |  | X |  |  | X |
| Serologic testing for syphilis (performed locally) | X |  |  |  | X |  |  |  | X |  |  | X |
| Stored serum sample**^[196]^** for future HHV-8 testing | X |  |  |  | X |  |  |  | X |  |  | X |
|  |  |  |  |  |  |  |  |  |  |  |  |  |
| **Body composition substudy (n=300)** |  |  |  |  |  |  |  |  |  |  |  |  |
| Fasting**^[197]^** serum sample for total cholesterol, LDL, HDL, and VLDL cholesterol, triglycerides, serum glucose, C-peptide, and insulin | X |  |  |  | X |  | X |  | X |  |  | X |
| Fasting whole blood sample for hemoglobin A1c | X |  |  |  |  |  |  |  | X |  |  | X |
| Fasting venous lactate**^[198]^** | X |  |  |  | X |  |  |  | X |  |  | X |
| Fasting serum stored for future metabolic measurements  (leptin, free fatty acids, total and free testosterone, LDL subparticles, cortisol, thyroid stimulating hormone, free T4, insulin-like growth factor-1, osteocalcin, parathyroid hormone (including calcium), 25-hydroxy vitamin cholecalciferol, Na, K, HCO_3_, Cl for anion gap, bone specific alkaline phosphatase, serum cross-linked N and C telopeptide, adiponectin) | X |  |  |  |  |  |  |  | X |  |  | X |
| Body composition measurement by DEXA and 3-cut CT scan | X |  |  |  |  |  |  |  | X |  |  | X |
| For women of childbearing potential, pregnancy tests on serum or urine samples (within 14 days prior to the DEXA and CT scans, performed locally) | X |  |  |  |  |  |  |  | X |  |  | X |
| Body habitus assessment by provider | X |  |  |  |  |  |  |  | X |  |  | X |
| Waist and hip circumference | X |  |  |  |  |  |  |  | X |  |  | X |
| Self-reported physical exercise | X |  |  |  |  |  |  |  | X |  |  | X |
| Self-reported history of cosmetic procedures | X |  |  |  |  |  |  |  | X |  |  | X |
| Concomitant medications | X |  |  |  |  |  |  |  | X |  |  | X |

#### Appendix F. SMART Protocol Team

**Wafaa El-Sadr, M.D., M.P.H., Co-Chair**

Harlem AIDS Treatment Group

Harlem Hospital Center

Division of Infectious Diseases

MLK Pavilion, Room 3107

506 Lenox Avenue

New York, NY 10037

Phone: (212) 939-2936

Fax: (212) 939-2968

E-Mail: [wme1@columbia.edu](mailto:wme1@columbia.edu)

**James Neaton, Ph.D., Co-Chair**

CPCRA Statistical and Data Management Center/CCBR

University of Minnesota

2221 University Avenue, Suite 200

Minneapolis, MN 55414-1380
Phone: (612) 626-9040

Fax: (612) 624-2819

E-Mail: [jim@ccbr.umn.edu](mailto:jim@ccbr.umn.edu)

**Philip Andrew, R.N., B.S.**

North Jersey Community Research Initiative

393 Central Avenue, 3rd Floor

Newark, NJ 07103

Phone: (973) 483-3444x33

Fax: (973) 485-7080

E-Mail: [p.andrew@njcri.org](mailto:p.andrew@njcri.org)

**Abdel Babiker, Ph.D.**

MRC Clinical Trials Unit

222 Euston Road

London NW1 2DA

UK

Phone: 44.207.670.4719

Fax: 44.207.670.4817

E-mail: [a.babiker@ctu.mrc.ac.uk](mailto:a.babiker@ctu.mrc.ac.uk)

**William Burman, M.D.**

Denver Public Health Department

605 Bannock Street, Room 520

Denver, CO 80204-4507

Phone: (303) 436-8197
Fax: (303) 436-7194

E-Mail: [bburman@dhha.org](mailto:bburman@dhha.org)

**Calvin Cohen, M.D., M.Sc.**

Community Research Initiative of New England

320 Washington Street, Third Floor

Brookline, MA 02146

Phone: (617) 566-4004x211

Fax: (617) 566-8226

E-Mail: [CCohen@Crine.org](mailto:CCohen@Crine.org)

**David Cohn, M.D.**

Denver Public Health Department

605 Bannock Street, Room 540

Denver, CO 80204-4507

Phone: (303) 436-7204

Fax: (303) 436-7194

E-Mail: [dcohn@dhha.org](mailto:dcohn@dhha.org)

**David Cooper, AO, M.D. D.Sc**

National Centre for HIV Epidemiology and Clinical Research (NCHECR)

Second Floor, St. Vincent’s Hosp. Med. Ctr.

376 Victoria Street

Sydney NSW 2010 AUSTRALIA

Phone: 61.2.9332.4648

FAX: 61.2.9360.1749

E-mail:	[dcooper@nchecr.unsw.edu.au](mailto:dcooper@nchecr.unsw.edu.au)

**Fraser Drummond, MBChB MRCA DA**

Therapeutic and Vaccine Reseach Program

National Centre in HIV Epidemiology and Clinical Research (NCHECR)

376 Victoria Street

Darlinghurst NSW 2010, AUSTRALIA

Phone: +612 9332 4648

Fax: +612 9380 5399

E-mail: [fdrummond@nchecr.unsw.edu.au](mailto:fdrummond@nchecr.unsw.edu.au)

**Fred Gordin, M.D.**

Washington Regional AIDS Program

Infectious Diseases (151B)

VA Medical Center

50 Irving Street, NW

Washington, DC 20422

Phone: (202) 745-8301

Fax: (202) 745-8694

E-Mail: [fred.gordin@med.va.gov](mailto:fred.gordin@med.va.gov)

**Birgit Grund, Ph.D.**

CPCRA Statistical and Data Management Center/CCBR

University of Minnesota

2221 University Avenue, Suite 200

Minneapolis, MN 55414-1380

Phone: (612) 626-8622

Fax: (612) 626-8892

E-Mail: [birgit@ccbr.umn.edu](mailto:birgit@ccbr.umn.edu)

**Richard Hafner, M.D.**

NIH, NIAID, DAIDS, TRP, OIRB

6700-B Rockledge Drive -MSC 7620

Bethesda, MD 20892-7620

Phone: (301) 435-3766

Fax: (301) 402-3171

E-Mail: [rhafner@niaid.nih.gov](mailto:rhafner@niaid.nih.gov)

**Carlton Hogan**

CPCRA Statistical and Data Management Center/CCBR

University of Minnesota

2221 University Avenue, Suite 200

Minneapolis, MN 55414-1380

Phone: (612) 626-8899

Fax: (612) 626-8892

E-Mail: [carlton@ccbr.umn.edu](mailto:carlton@ccbr.umn.edu)

**Jennifer Hoy, M.B.B.S. F.R.A.C.P.**

Infectious Disease Unit

The Alfred Hospital

Commercial Road

Melbourne, Victoria, 3004

Australia

Phone: 61-3-9276-6900

Fax: 61-3-9529-7019

E-Mail: [jennifer.hoy@med.monash.edu.au](mailto:jennifer.hoy@med.monash.edu.au)

**Karin L. Klingman, M.D.**

NIH, NIAID, DAIDS, TRP, HIVRB

6700-B Rockledge Drive - MSC 7624

Bethesda, MD 20892-7620

Phone: (301) 435-3772

Fax: (301) 435-9282

E-Mail: [kklingman@niaid.nih.gov](mailto:kklingman@niaid.nih.gov)

**Jay Kostman, M.D.**

UPHS/Presbyterian Medical Center

Wright Saunders Building, Suite W-250

Division of Infectious Diseases

39th and Market Streets

Philadelphia, PA 19104

Phone: (215) 662-8693

Fax: (215) 243-3272

E-Mail: [kostman@mail.med.upenn.edu](mailto:kostman@mail.med.upenn.edu)

**Jens Lundgren, M.D., DMSc**

Coordinating Centre

Department of Infectious Diseases 144

Hvidovre University Hospital

DK-2650 Hvidovre

Denmark

Phone: 45.36.32.30.15

Fax: 45.36.47.33.40

E-Mail: [jdl@cphiv.dk](mailto:jdl@cphiv.dk)

**Ana Martinez, R.Ph.**

Chief, Pharmaceutical Affairs Branch

NIH, NIAID, DAIDS, PAB

Room 5115

6700-B Rockledge Drive, MSC 7620

Bethesda, MD 20892-7620

Phone: (301) 435-3734 or (301) 435-3734

Fax: (301) 402-1506

E-mail: [amartinez@niaid.nih.gov](mailto:amartinez@niaid.nih.gov)

**Carol Miller, M.P.H.**

CPCRA Statistical and Data Management Center/CCBR

University of Minnesota

2221 University Avenue, Suite 200

Minneapolis, MN 55414-1380

Phone: (612) 626-8048, 1-800-511-0311

Fax: (612) 626-8892

E-Mail: carol@ccbr.umn.edu

**Christopher Mullin, M.S.**

CPCRA Statistical and Data Management Center/CCBR

University of Minnesota

2221 University Avenue, Suite 200

Minneapolis, MN 55414-1380

Phone: (612) 626-7966, 1-800-511-0311

Fax: (612) 626-8892

E-Mail: [chrism@ccbr.umn.edu](mailto:chrism@ccbr.umn.edu)

**Jacqueline Neuhaus, M.S.**

CPCRA Statistical and Data Management Center/CCBR

University of Minnesota

2221 University Avenue, Suite 200

Minneapolis, MN 55414-1380

Phone: (612) 626-8893, 1-800-511-0311

Fax: (612) 626-8892

E-Mail: [jacquie@ccbr.umn.edu](mailto:jacquie@ccbr.umn.edu)

**Mollie Poehlman, M.S.**

CPCRA Statistical and Data Management Center/CCBR

University of Minnesota

2221 University Avenue, Suite 200

Minneapolis, MN 55414-1380

Phone: (612) 626-8982, 1-800-511-0311

Fax: (612) 626-8892

E-Mail: [mollie@ccbr.umn.edu](mailto:mollie@ccbr.umn.edu)

**Ronald J. Prineas, M.D., Ph.D.**

EPICARE

Wake Forest University

School of Medicine

Department of Public Health Sciences

2000 West First Street, Suite 505

Winston-Salem, NC 27104

Phone: (336) 716-0830

Fax: (336) 716-0834

E-Mail: [rprineas@wfubmc.edu](mailto:rprineas@wfubmc.edu)

**Claire Rappoport, M.A.**

10 Inyo Street

Brisbane, CA 94005

Phone: (415) 656-1401

Fax: (415) 656-4308

E-Mail: [Clairer@itsa.ucsf.edu](mailto:Clairer@itsa.ucsf.edu)

**Barry Schmetter, B.S.**

CPCRA Operations Center

Social & Scientific Systems

8757 Georgia Avenue, 12^th^ Floor

Silver Spring, MD 20910-3714

Phone: (301) 628-3317

Fax: (301) 230-7190

E-mail: [BSchmetter@s-3.com](mailto:Bschmetter@s-3.com)

**Janis Stewart, R.N.**

CPCRA Clinical Site Monitoring Group

Pharmaceutical Product Development, Inc.

3151 South 17th Street Extension

Wilmington, NC 28412

Phone: (910) 772-7051

Fax: (919) 654-9861

E-Mail: [janis.stewart@wilm.ppdi.com](mailto:debra.farrow@wilm.ppdi.com)

References

1. ^1^	Keystone National Policy Dialogue on Establishment of Studies to Optimize Medical Management of HIV Infection. The Keystone Center, July 1996.
2. Cohen OJ. Antiretroviral therapy: time to think strategically. Lancet 2000;132:320-2.
3. Folkers G, Morales J. HIV treatment guidelines updated for adults and adolescents available on the world wide web. Available from: [http://www.hivatis.org](http://www.hivatis.org/). Accessed 2001.
4. Carpenter C, Cooper D, Fischl M, et al. Antiretroviral therapy in adults: updated recommendations of the International AIDS Society-USA Panel. JAMA 2000;283:381-90.
5. Gazzard B, Moyle G. 1998 revision to the British HIV Association guidelines for antiretroviral treatment of HIV seropositive individuals. Lancet 1998;352:314-6.
6. Guidelines for the use of antiretroviral agents in HIV-1 infected adults and adolescents. July 2003 (http://AIDSinfo.nih.gov).
7. Mocroft A, Katalama C, Johnson AM, et al. AIDS across Europe, 1994-98: the EuroSIDA study. Lancet 2000;356:291-6.
8. Mellors JW, Rinaldo CR, Gupta P, et al. Prognosis in HIV-1 infection predicted by the quantity of virus in plasma. Science 1996;272:1167-70.
9. MacArthur RD, Perez G, Walsmley S, et al. CD4+ cell count is a better predictor of disease progression than HIV RNA level in persons with advanced HIV infection on highly active antiretroviral therapy. [Abstract] 8^th^ Conference on Retroviruses and Opportunistic Infections; February 2001; Chicago, Illinois.
10. Grabar S, Le Moing V, Goujard C, et al. Clinical outcome of patients with HIV-1 infection according to immunologic and virologic response after 6 months of highly active antiretroviral therapy. Ann Intern Med 2000;133:401-10.
11. Phair J, Munoz A, Detels R, et al. and the Multicenter AIDS Cohort Study Group. Risk of *Pneumocystis carinii* pneumonia among men infected with HIV-1. N Engl J Med 1990;322:1607.
12. Weverling GJ, Mocroft A, Ledergerber B, et al. Discontinuation of Pneumocystis carinii pneumonia prophylaxis after start of highly active antiretroviral therapy in HIV-1 infection. EuroSIDA Study Group. Lancet 1999;353:1293-8.
13. Furrer H, Egger M, Opravil M, et al. Discontinuation of primary prophylaxis for Pneumocystis carinii pneumonia in HIV-1 infected adults treated with combination antiretroviral therapy. N Engl J Med 1999;340:1301-6.
14. Kirk O, Lundgren JD, Pedersen C. Can chemoprophylaxis against opportunistic infections be discontinued after an increase in CD4 cells induced by highly active antiretroviral therapy? AIDS 1999;13:1647-51.
15. El-Sadr WM, Burman WJ, Grant LB, et al. Discontinuation of prophylaxis for Mycobacterium avium complex disease in HIV-infected patients who have a response to antiretroviral therapy. N Engl J Med 2000;342:1085-92.
16. Furrer H, Telenti A, Rossi M, et al. Discontinuing or withholding primary prophylaxis against *Mycobacterium avium* in patients on successful antiretroviral combination therapy. The Swiss HIV Cohort Study. AIDS 2000;14:1409-12.
17. Lederberger B, Mocroft A, Reiss P, et al. Discontinuation of secondary prophylaxis against Pneumocystis carinii pneumonia in patients with HIV infection who have a response to antiretroviral therapy. N Engl J Med 2001; 344:168-174.
18. Kirk O, Reiss P, Uberti-Foppa C, et al. Safe interruption of maintenance therapy against previous infection with four common HIV-associated opportunistic pathogens during potent antiretroviral therapy. Ann Intern Med 2002;137:239-250.
19. Lederman MM, Valdez H. Immune restoration with antiretroviral therapies. Implications for management. JAMA 2000;284:223-8.
20. Phillips AN, Staszewski S, Weber R, et al. Viral load changes in response to antiretroviral therapy according to the baseline CD4+ lymphocyte count and viral load. [Abstract PL3.4] 5^th^ International Congress on Drug Therapy in HIV Infection; October 22-26 2000; Glasgow, United Kingdom.
21. The Antiretroviral Therapy (ART) Collaboration. Prognostic significance of early immunologic and virologic response in HIV-1 infected patients starting potent antiretroviral therapy. Lancet (in press).
22. Lucas GM, Chaisson RE, Moore RD. Highly active antiretroviral therapy in a large urban clinic: risk factors for virologic failure and adverse drug reactions. Ann Intern Med 1999;131:81-7.
23. Bartlett J, Demasi F, Quinn J, et al. Meta-analysis of efficacy of triple combination therapy in antiretroviral-naïve HIV-1-infected adults. [Abstract] 7^th^ Conference on Retroviruses and Opportunistic Infections; January 2000; San Francisco, California.
24. Deeks SG, Hecht FM, Swanson M, et al. HIV RNA and CD4 cell count response to protease inhibitor therapy in an urban AIDS clinic: response to both initial and salvage therapy. AIDS 1999;13:F35-43.
25. Baxter J, Mayers D, Wentworth D, et al. A randomized study of antiretroviral management based on plasma genotypic antiretroviral resistance testing in patients failing therapy. AIDS 2000;14:F83-93.
26. Durant J, Clevenbergh P, Halfon P, et al. Drug-resistance genotyping in HIV-1 therapy: the VIRADAPT randomised controlled trial. Lancet 1999;353:2195-9.
27. Mocroft A, Phillips AN, Miller V, et al. The use of an response to second-line protease inhibitor regimens: results from the EuroSIDA Study. AIDS 2001;15:201-209.
28. Mocroft A, Phillips AN, Friis-Moller N, et al. Response to antiretroviral therapy among patients exposed to three classes of antiretrovirals: results from the EuroSIDA Study. Antivir Ther 2002; 1:21-30.
29. Wong JK, Hezareh M, Gunthard HF, et al. Recovery of replication-competent HIV despite prolonged suppression of plasma viermia. Science 1997;278:1291-5.
30. Finzi D, Hermankova M, Pierson T, et al. Identification of a reservoir for HIV-1 in patients on highly active antiretroviral therapy. Science 1997;278:1295-300.
31. Martinez-Picado J, DePasquale MP, Kartsonis NA, et al. Selection of antiretroviral resistance in the latent reservoir of HIV type 1 during successful therapy. [Abstract 238] 7^th^ Conference on Retroviruses and Opportunistic Infections; January 2000; San Francisco, California.
32. John H, Muller NJ, Opravil M, et al. Indinavir urinary stones as origin of upper urinary tract obstruction. Urol Int 1997;59(4):257-9.
33. Havlir D, Gilbert P, Bennett K, et al. Randomized trial of continued indinavir (IDV)/ZDV/3TC vs. switch to IDV/ddI/d4T or IDV/ddI/d4T + hydroxyurea in patients with viral suppression. [Abstract 456] 7th Conference on Retroviruses and Opportunistic Infections; January 2000; San Francisco, California.
34. Brinkman K, Smeitink JA, Romihn J, Reiss P. Mitochondrial toxicity induced by nucleoside-analogue reverse transcriptase inhibitors is a key factor in the pathogenesis of antiretroviral-therapy-related lipodystrophy. Lancet 1999;354:1112-5.
35. Carr A, Samaras K, Thorisdottir A, et al. Diagnosis, prediction, and natural course of HIV-1 protease-inhibitor-associated lipodystrophy, hyperlipidemia, and diabetes mellitus: a cohort study. Lancet 1999;353:2093-9.
36. Henry K, Melroe H, Huebsch J, et al. Severe premature coronary artery disease with protease inhibitors. Lancet 1998;351:1328.
37. Carr A, Miller J, Cooper DA. Osteopenica in HIV-infected men: association with lactic acidemica and lower weight pre-antiretroviral therapy. [Abstract] 2^nd^ International Workshop on Adverse Drug Reactions and Lipodystrophy; September 2000; Toronto, Canada.
38. Carr A, Miller J, Law M, Cooper DA. A syndrome of lipoatrophy, lactic acidemia and liver dysfunction associated with HIV nucleoside analogue therapy: contribution to protease inhibitor-related lipodystrophy syndrome. AIDS 2000;14:F25-32.
39. Brinkman K, Smeitink JA, Romijn JA, Reiss P. Mitochondrial toxicity induced by nucleoside-analogue reverse-transcriptase inhibitors is a key factor in the pathogenesis of antiretroviral-therapy-related lipodystrophy. Lancet 1999;354:1112-5.
40. Friis-Moller N, Weber R, D'Arminio Monforte A, et al. Exposure to HAART is associated with an increased risk of myocardial infarction: the DAD study. 10^th^ Conference on Retroviruses and Opportunistic Infections; February 2003; Boston, MA.
41. Paterson D, Swindells S, Mohr J, et al. Adherence to protease inhibitor therapy and outcomes in patients with HIV infection. Ann Intern Med 2000;133:21-30.
42. Combs DL, O'Brien RJ, Geiter LJ. USPHS tuberculosis short-course chemotherapy trial 21: effectiveness, toxicity, and acceptability. Ann Intern Med 1990;112:397-406.
43. Mannheimer S, Friedland G, Matts J, et al. Self-reported antiretroviral adherence correlates with HIV viral load and declines over time. [Abstract TuOrB421] 13^th^ International AIDS Conference; July 2000; Durban, South Africa.
44. Catz SL, Kelleg JA, Bogart LM, et al. Patterns correlates, and barriers to medication adherence among persons prescribed new treatments for HIV disease. Health Psychology 2000;19(2)124-33.
45. Mellors JW, Munoz A, Giorgi JV, et al. Plasma viral load and CD4+ lymphocytes as prognostic markers of HIV-1 infection. Ann Intern Med 1997;126:946-54.
46. Panel on Clinical Practices for Treatment of HIV Infection. Report of the NIH Panel to define principles of therapy for HIV infection. MMWR 1998;4,(No. RR-5).
47. Montaner J, Reiss P, Cooper D, et al. A randomized double-blind trial comparing combinations of nevirapine, didanosine, and zidovudine for HIV infected patients: the INCAS trial. JAMA 1998;279:930-7.
48. Parkin NT, Deeks SG, Wrin MT, et al. Detection of reduced drug susceptibility at low viral loads (<1000 copies/mL) prior to virologic failure during salvage therapy. [Abstract 31] Antiviral Therapy 2000;5(Suppl 2).
49. Hirsch MS, Brun-Vezinet F, D’Aquila R, et al. Antiretroviral drug resistance testing in adult HIV-1 infection. Recommendations of International AIDS Society-USA Panel. JAMA 2000;283:2417-26.
50. Condra JH, Schleif WA, Blahy OM, et al. In vivo emergence of HIV-1 variants resistant to multiple protease inhibitors. Nature 1995;374:569-71.
51. Molla A, Korneyeva M, Gao Q, et al. Ordered accumulation of mutations in HIV protease confers resistance to ritonavir. Nat Med 1996;2:760-6.
52. Miller V, Sabin C, Hertogs K, et al. Antiretroviral treatment interruptions in patients with treatment failure. [Abstract 25] Antiviral Therapy 2000;5(Suppl 2).
53. Cohen C, Hunt S, Sension M, et al. Phenotypic resistance testing signficantly improves response to therapy. [Abstract] 7^th^ Conference on Retroviruses and Opportunistic Infections; January 2000; San Francisco, California.
54. Havlir D, Hellman NS, Petropoulos CM, et al. Drug susceptibility in HIV infection after viral rebound in patients receiving indinavir containing regimens. JAMA 2000;283:229-34.
55. Gallant JE, Hall C, Barnett S, Raines C. Ritonavir/saquinavir as salvage therapy after failure of initial protease inhibitor regimen. [Abstract 427] 5^th^ Conference on Retroviruses and Opportunistic Infections; February 1998; Chicago, Illinois.
56. Henry K, Kane E, Melrose H, et al. Experience with ritonavir/saquinavir based regimen for the treatment of HIV infection in subjects developing increased viral loads while receiving nelfinavir. [Abstract I-204] 37^th^ ICAAC; 1997; Toronto, Canada.
57. Tebas P, Patick AK, Kane EM, et al. Virologic responses to a ritonavir-saquinavir-containing regimen in patients who had previously failed nelfinavir. AIDS 1999;13:F23-8.
58. Hammer S, Squires K, Degruttola V, et al. Randomized trial of abacavir (ABC) & nelvinavir (NVF) in combination with efavirenz (EFV) & adefovir dipivoxil (ADV) as salvage therapy in patients with virologic failure receiving indinavir (IDV). [Abstract 490] 6^th^ Conference on Retroviruses and Opportunistic Infections; 1999; Chicago, Illinois.
59. Miller V, Rottmann C, Hertogs K, et al. Mega-HAART, resistance and drug holidays. Antivir Ther 1999;1:27.
60. Deeks SG, Wrin T, Hoh R, et al. Virologic and immunologic evaluation of structured treatment interruptions in patients experiencing long-term virologic failure. [Abstract LB10] 7^th^ Conference on Retroviruses and Opportunistic Infections; January 2000.
61. Lawrence J, Mayers DL, Huppler-Hullsiek K, et al. A multicenter randomized trial of structured treatment interruption in patients with multi-drug resistant human immunodeficiency virus. N Eng J Med (in press).
62. Ruiz L, Ribera E, Bonjoch A, et al. Virlogical and immunological benefit of a salvage therapy that includes kaletra plus fortovase preceded or not by antiretroviral therapy interruption in advanced HIV-infected patients with multidrug resistance mutations (48 weeks of follow-up). XI International HIV Drug Resistance Workshop, Seville, Spain, July 2002. Antivir Ther 2002;7:S126.
63. Katalama C, Dominguez S, Durvivier C, et al. Long-term benefit of treatment interruption in salvage therapy (GUGHAART ANRS 097). 10^th^ Conference on Retroviruses and Opportunistic Infections, February 2003, Boston, MA.
64. Lundgren JD, Vella S, Paddam A, et al. Interruption/stopping antiretroviral therapy and risk of clinical disease: Results from the EuroSIDA Study. 9^th^ Conference on Retroviruses and Opportunistic Infections, February 2002, Seattle, WA.
65. Hatano H, Vogel S, Metcalf J, et al. Plasma viral loads approximate pre-HAART levels after discontinuation of HAART. [Abstract] 7^th^ Conference on Retroviruses and Opportunistic Infections; January 2000; San Francisco, California.
66. Tebas P, Henry K, Mondy K, et al. Effect of prolonged discontinuation of successful antiretroviral therapy on CD4+ T cell decline in human imunodeficiency virus-infected patients: implications for intermittent therapeutic strategies. J Infect Dis 2002;186:851-854.
67. Colven R, Harrington RD, Spach DH, et al. Retroviral rebound syndrome after cessation of suppressive antiretroviral therapy in three patients with chronic HIV infection. Ann Intern Med 2000;133:430-4.
68. Kilby JM, Goepfert PA, Miller AP, et al. Recurrence of the acute HIV syndrome after interruption of antiretroviral therapy in a patient with chronic HIV infection: a case report. Ann Int Med 2000; 133:435-38.
69. Douek DC, Brenchley JM, Betts MR, et al. HIV preferentially infects HIV-specific CD4+ T cells. Nature 2002; 417:95-98.
70. Yerly S, Kaiser L, Perneger TV, et al. Time of initiation of antiretroviral therapy: impact on HIV-1 viraemia. AIDS 2000;14:243-9.
71. Miller V, Staszewski S, Sabin C, et al. CD4 lymphocyte count as a predictor of the duration of highly active antiretroviral therapy-induced suppression of human immunodeficiency virus load. J Infect Dis 1999;180:530-3.
72. Moore R, Keruly J, Bartlett, J, et al. Start HAART early (CD4 > 350 cells/mm^3^) or later? Evidence for greater effectiveness if started early. [Abstract] 7^th^ Conference on Retroviruses and Opportunistic Infections; January 2000; San Francisco, California.
73. Pollard R. Factors predictive of durable HIV suppression in a randomized double-blind trial with nevirapine (NVP), zidovudine (ZDV), and lamivudine (3TC) in treatment-naïve (ARV‑n) patients with advanced AIDS. [Abstract] 7^th^ Conference on Retroviruses and Opportunistic Infections, January 2000.
74. Staszewski S, Miller V, Sabin C, et al. Determinants of sustainable CD4 lymphocyte count increases in response to antiretroviral therapy. AIDS 1999;13:951-6.
75. Yip B, Wood E, Montessori V, et al. Continued suppression of plasma viral load is needed to achieve an optimal CD4 response among patients receiving triple drug antiretroviral therapy. [Abstract] 7^th^ Conference on Retroviruses and Opportunistic Infections; January 2000; San Francisco, California.
76. Meibohm A, Alexander J Jr., Robertson M, et al. Early versus late initiation of indinavir (IDV) in combination with zidovudine (ZDV) and lamivudine (3TC) in HIV-1 infected adults. [Abstract] 7^th^ Conference on Retroviruses and Opportunstic Infections, January 2000; San Francisco, California.
77. Autran B, Carcelain G, Li T, et al. Positive effects of combined antiretroviral therapy on CD4 T cell homeostasis and function in advanced HIV disease. Science 1997;277:112-6.
78. Miller V, Mocroft A, Reiss P, et al. Relations among CD4 lymphocyte count nadir, antiretroviral therapy, and HIV-1 disease progression: results from the EuroSIDA Study. Ann Intern Med 1999;130:570-7.
79. Palella FJ, Delaney KM, Moorman AC, et al. Declining morbidity and mortality among patients with advanced immunodeficiency virus infection. N Engl J Med 1998;338:853-60.
80. Mocroft A, Vella S, Benfield TL, et al. Changing patterns of mortality across Europe in patients infected with HIV-1. EuroSIDA Study Group. Lancet 1998;352:1725-30.
81. Lang W, Perkins H, Anderson RE, et al. Patterns of T lymphocyte changes with human immunodeficiency virus infection: from seroconversion to the development of AIDS. J Acquir Immune Defic Snydr 1989;2:63-9.
82. Barbaro G, Di Lorenso G, Grisorio B, Barbarini G. Clinical meaning of ventricular ectopic beats in the diagnosis of HIV-related myocarditis: a retrospective analysis of Holter electrocardiographic recordings, echocardiographic parameters, histopathological and virological findings. Cardiologia 1996;41:1199-207.
83. Herskoitz A. Cardiomyopathy and other symptomatic heart diseases associated with HIV infection. Curr Opin Cardiol 1996;11:325-31.
84. Kannel WB, Abbott RD. Incidence and prognosis of unrecognised myocardial infarction. An update on the Framingham Study. N Engl J Med 1984;311:1144.
85. Grimm RH, Tillinghast S, Daniels K, et al. Unrecognized myocardial infarction: experience in the Multiple Risk Factor Intervention Trial (MRFIT). Circulation 1987;75 (Suppl II):II6-8.
86. Kannel WB, Gordon T, Castelli WP, Margolis JR. Electrocardiographic left ventricular hypertrophy and risk of coronary heart disease: the Framingham Study. Ann Intern Med 1970;72:813-22.
87. Prineas RJ, Rautaharju PM, Grandits G, Crow R for the MRFIT Research Group. Independent risk for cardiovascular disease predicted by modified continuous score electrocardiographic criteria for 6-year incidence and regression of LVH among clinical disease free men: 16 year followup for the MRFIT. J Electrocardiology 2001;34:91-101.
88. CDC. 1993 Revised classification system for HIV infection and expanded surveillance case definition for AIDS among adolescents and adults. Morbidity and Mortality Weekly Report CDC Surveillance Summary 1993;41(No. RR-17):1-19.
89. Green LA, Rhame F, Price RW, et al. Experience with a cross-study endpoint review committee for AIDS clinical trials. AIDS 1998;12:1983-90.
90. Neaton J, Wentworth D, Rhame F, et al. Methods of studying interventions: considerations in choice of a clinical endpoint for AIDS clinical trials. Stat Med 1994;13:2107-25.
91. Hammer SM, Squires KE, Hughes MD, et al. A controlled trial of two nucleoside analogues plus indinavir in persons with human immunodeficiency virus infection and CD4 cell counts of 200 per cubic millimeter or less. N Engl J Med1997;337:725-33.
92. Cameron DW, Heath-Chiozzi M, Danner S, et al. Randomised placebo-controlled trial of ritonavir in advanced HIV-1 disease. Lancet 1998;351:543-9.
93. Babiker A. MRC HIV Clinical Trials Centre, London. Personal communication, 1998.
94. Lundgren J, Mocroft A, Philips A. Department of Infectious Diseases at Hvidovre Hospital, Copenhagen, Denmark and Royal Free Centre for HIV Medicine & Department of Primary Care and Population Sciences, University College London, London, U.K. Personal Communication, 1998.
95. Kahn JO, Cheng DW, Mayer K, et al for the 806 Investigator Team. Evaluation of HIV-1 Immunogen, an immunologic modifier, administered to patients with HIV having 300 to 549 x 10^6^/L CD4 cell counts. JAMA 2000:284:2193-202.
96. Shih J. Sample size calculation for complex clinical trials with survival endpoints. Cont Clinical Trials 1995;16:395-407.
97. Refer to Section 4.7 for guidelines on how to manage patients who become pregnant during follow-up.
98. For patients enrolled in the Body Composition substudy, this sample will be collected under fasting requirements.
99. For patients enrolled in the Body Composition substudy, this sample will be collected under fasting requirements.
100. Centers for Disease Control and Prevention. 1999 USPHS/IDSA guidelines for the prevention of opportunistic infections in persons infected with human immunodeficiency virus: U.S. Public Health Service (USPHS) and Infectious Disease Society of America (IDSA). MMWR 1999;48(No. RR-10):1-30.
101. Public Health Service Task Force Recommendations for the Use of Antiretroviral Drugs in Pregnant HIV-1 Infected Women for Maternal Health and Interventions to Reduce Perinatal HIV-1 Transmission in the United States. Available from: http://www.hivatis.org. Accessed January 24, 2001.
102. American College of Obstetricians and Gynecologist Technical Bulletin. Preconceptional care. Number 205, May 1995.
103. Dybul M, Nis-Kraske E, Daucher M, e t al. Long-cycle structured intermittent versus continuous highly active antiretroviral therapy for the treatment of chronic infection with human immunodeficiency virus: effects on drug toxicity and on immunologic and virologic parameters. J Infect Dis 2003;188:388-96.
104. O’Brien PC, Fleming TR. A multiple testing procedure for clinical trials. Biometrics 1979;35:549-56.
105. Lan KKG, DeMets DL. Discrete sequential boundaries for clinical trials. Biometrika 1983;70:659-63.
106. Hays RD, Cunningham WE, Sherbourne CD, et al. Health-related quality of life in patients with human immunodeficiency virus infection in the United States: results from the HIV cost and services utilization study. Am J Med 2000;108:714-22.
107. Cohen C, Revicki DA, Nabulsi A, et al. A randomized trial of the effect of ritonavir in maintaining quality of life in advanced HIV disease. AIDS 1998;12:1495-502.
108. Chatterton ML, Scott-Lennox J, Wu AW, Scott J. Quality of life and treatment satisfaction after the addition of lamivudine or lamivudine plus lovaride to zidovudine-containing regimens in treatment-experienced patients with HIV infection. Pharmacoeconomics 1999;15 Suppl 1:67-74.
109. Wu AW, Rubin HR, Matthews WC, et al. Functional status and well-being in a placebo-controlled trial of zidovudine in early symptomatic HIV infection. J Acquir Immune Defic Syndr 1993;6:452-8.
110. Freedberg KA, Scharfstein JA,. Seage GR 3^rd^, et al. The cost-effectiveness of preventing AIDS-related opportunistic infections. JAMA 1998 279:130-6.
111. Berry SH, Brown JA, Athey L, et al. HCSUS Baseline Patient Questionnaire Documentation. Santa Monica, Calif: RAND; 1998. MR-1090-AHCPR.
112. Shapiro MF, Morton SC, McCaffrey DF, et al. Variations in the care of HIV-infected adults in the United States: results from the HIV Cost and Services Utilization Study. JAMA 1999 281:2305-15.
113. Bayoumi AM, Redelmeier DA. Economic Methods for Measuring the Quality of Life Associated with HIV Infection. Quality of Life Research. 8:471-80.
114. Bozzette SA, Berry SH, Duan N, et al. The care of HIV-infected adults in the United States. HIV Cost and Services Utilization Study Consortium. N Engl J Med 1998;339:1897-904.
115. Gold, MR, Siegel JE, Russell LB, Weinstein MC. Cost-Effectiveness in Health and Medicine. New York: Oxford University Press; 1996.
116. Erbelding EJ, Stanton D, Quinn TC, Rompalo A. Behavioral and biologic evidence of persistent high-risk behavior in an HIV primary care population. AIDS 2000;14:297-301.
117. Committee on HIV Prevention Strategies in the United States IoM. No time to lose: getting more from HIV prevention. Washington DC: National Academy Press; 2001:1-227.
118. Quinn TC, Wawer MH, Sewankambo N, et al. Viral load and heterosexual transmission of human immunodeficiency virus type 1. Rakai Project Study Group. N Engl J Med 2000;342:921-9.
119. Garcia PM, Kalish LA, Pitt J, et al. Maternal levels of plasma human immunodeficiency virus type 1 RNA and the risk of perinatal transmission. Woman and Infants Transmission Study Group. N Engl J Med 1999;341:394-402.
120. Miller M, Meyer L, Boufassa F, et al. Sexual behavior changes and protease inhibitor therapy. AIDS 2000;14:F33-9.
121. Scheer S, Chu PL, Klausner JD, et al. Effect of highly active antiretroviral therapy on diagnoses of sexually transmitted diseases in people with AIDS. Lancet 2001;357:432-5.
122. Centers for Disease Control and Prevention. Gonorrhea among men who have sex with men - selected sexually transmitted disease clinics, 1993-1996. MMWR 1997;46:889-92.
123. Centers for Disease Prevention and Control. Resurgent bacterial sexually transmitted disease among men who have sex with men - King County, Washington, 1997-1999. MMWR 1999;48:773-7.
124. McFarland W, Schwartz S, Kellogg TA, et al. Implications of highly active antiretroviral treatment for HIV prevention: the case of men who have sex with men in San Francisco. [Abstract] 13^th^ International AIDS Conference; July 2000; Durban, South Africa.
125. Little SJ, Routy JP, Daar ES, et al. Antiretroviral drug susceptibility and response to initial therapy among recently HIV-infected sucjects in North America. 8th Conference on Retroviruses and Opportunistic Infections; February 2001; Chicago, Illinois.
126. Catania JA, Gibson DR, Chitwood DD, Coates TJ. Methodological problems in AIDS behavioral research: influences on measurement error and participation bias in studies of sexual behavior. Psychol Bull 1990;108:339-62.
127. Carroll KC, Aldeen WE, Morrison M, et al. Evaluation of the Abbott LCx ligase chain reaction assay for detection of Chlamydia trachomatis and Neisseria gonorrhoeae in urine and genital swab specimens from a sexually transmitted disease clinic population. J Clin Microbiol 1998;36:1630-3.
128. Xu K, Glanton V, Johnson SR, et al. Detection of *Neisseria gonorrhoeae* infection by ligase chain reaction testing of urine among adolescent women with and without *Chlamydia trachomatis* infection. Sex Transm Dis 1998;25:533-8.
129. van Doornum GJ, Schouls LM, Pijl A, Cairo I, Buimer M, Bruisten S. Comparison between the LCx Probe system and the COBAS AMPLICOR system for detection of *Chlamydia trachomatis* and *Neisseria gonorrhoeae* infections in patients attending a clinic for treatment of sexually transmitted diseases in Amsterdam, The Netherlands. J Clin Microbiol 2001;39:829-35.
130. Smith KR, Ching S, Lee H, et al. Evaluation of ligase chain reaction for use with urine for identification of *Neisseria gonorrhoeae* in females attending a sexually transmitted disease clinic. J Clin Microbiol 1995;33:455-7.
131. Martin JN, Ganem DE, Osmond DH, et al. Sexual transmission and the natural history of human herpesvirus 8 infection. N Engl J Med 1998;338:948-54.
132. 1998 Guidelines for Treatment of Sexually Transmitted Diseases. MMWR 1998; 47 (no. RR-1):1-128.
133. Flaks R, Burman W, Gourley P, et al. HIV transmission risk factors among those in HIV clinical care, National STD Prevention Conference; December 4-7 2000 Milwaukee, Wisconsin.
134. Wenger NS, Kusseling FS, Beck K, Shapiro MF. Sexual behavior of individuals infected with the human immunodeficiency virus. The need for intervention. Arch Intern Med 1994;154:1849-54.
135. Carr A, Samaras K, Thorisdottir A, et al. Diagnosis, prediction, and natural course of HIV-1 protease-inhibitor-associated lipodystrophy, hyperlipidemia, and diabetes mellitus: a cohort study. Lancet 1999;353:2093-9.
136. Engelson E, Kotler DP, Tan YX, et al. Altered body fat distribution in HIV infection: regional body composition measurements by whole body MRI and DXA scans [Abstract 32181]. 12th World AIDS Conference; 1998 Geneva, Switzerland.
137. Huang JS, Reitschel P, Hadigan CM, et al. Increased abdominal visceral fat is associated with reduced bone density in HIV-infected men with lipodystrophy. AIDS 2001;15:975-82.
138. Carr A, Samaras K, Burton S, et al. A syndrome of peripheral lipodystrophy, hyperlipidemia, and insulin resistance in patients receiving HIV protease inhibitors. AIDS 1998;12:F51-8.
139. Visnegarwala F, Krause K, Musher DM. Severe diabetes associated with HIV-1 protease inhibitor therapy. Annals of Int Med 1997;127:947.
140. Miller KD, Jones E, Yanovski JA, et al. Visceral abdominal-fat accumulation associated with use of Indinavir. Lancet 1998;351:871-5.
141. Dong K, Flynn MM, Dickinson BP, et al. Changes in body habitus in HIV+ women after initiation of protease inhibitor therapy. J Acquir Immune Defic Syndr 1999;21:107-13.
142. Hadigan C, Meigs JB, Corcoran C, et al. Metabolic abnormalities and cardiovascular disease risk factors in adults with human immunodeficiency virus infection and lipodystrophy. Clin Infect Dis 2001;32:130-9.
143. Henry K, Melroe H, Huebsch J, et al. Severe premature coronary artery disease with protease inhibitors. Lancet 1998;351:1328.
144. Raghavan SS, Stanton M, Lester K, et al. The association of personal and or family history of diabetes, cardiovascular complications and obesity with the development of anti-retroviral body habitus changes and metabolic complications. [Abstract WePeB4276] 13^th^ International AIDS Conference; July 2000; Durban, South Africa.
145. Sekhar RV, Jahoor F, White AC, et al. Metabolic basis of HIV-lipodystrophy syndrome. Am J Physiol Endocrinol Metab 2002. 283(2):E332-7.
146. Corcoran C, Anderson EJ, Burrows B, et al. Comparison of total body potassium with other techniques for measuring lean body mass in men and women with AIDS wasting. Am J Clin Nutr 2000;72:1053-8.
147. Haarbo J, Gotfredsen A, Hassager C, et al. Validation of body composition by dual energy X-ray absorptiometry (DEXA). Clin Physiol 1991;11:331-41.
148. Chilibeck P, Calder A, Sale DG, et al. Reproducibility of dual-energy x-ray absorptiometry. Can Assoc Radiol J 1994;45:297-302.
149. Greenfield JR, Samaras K, Campbell LV, et al. Measuring abdominal fat: is a single CT slice enough? [Abstract] International Congress on Sport Science, Sports Medicine and Physical Education; September 7-12 2000; Brisbane, Australia.
150. Cann CE, Genant HK. Cross sectional studies of vertebral mineral using quantative computed tomography. J Comput Assit Tomogr 1982;6:216.
151. Faul DD, Couch JL, Cann CD, et al. Composition -selective reconstruction for mineral contenet in the axial and appendicular skeleton. J Comput Assist Tomogr 1982;6:202-3.
152. Hatano H, Miller KD, Yoder CP, et al. Metabolic and anthropometric consequences of interruption of highly active antiretroviral therapy*.* AIDS 2000; 14(13):1935-42..
153. Carr A, Hudson J, Chuah J, et al. for the PIILR investigators. HIV protease inhibitor substitution in patients with lipodystrophy: a randomised, multicentre, open-label study. AIDS. In press 2001.
154. Tebas P, Yarasheski KE, Henry K, et al. Evolution of multiple metabolic parameters after the switch of protease inhibitors to nevirapine. [Abstract ThPpB1485] 13^th^ International AIDS Conference; July 2000; Durban, South Africa..
155. Tebas P, Powderly WG, Calxton S, et al. Accelerated bone mineral loss in HIV-infected patients receiving potent anti-retroviral therapy. AIDS 2000;14:F63-7.
156. Carr A, Miller J, Law M, et al. A syndrome of lipoatrophy, lactic acidemia and liver dysfunction associated with HIV nucleoside analogue therapy: contribution to protease inhibitor-related lipodystrophy syndrome. AIDS 2000;14: F25-32.
157. Dusso A, Vidal M, Powderly WG, et al. Protease inhibitors inhibit conversion of 25(OH)-vitamin D to 1,25 (OH) 2-vitamin D. [Abstract O30]. 2nd International Workshop on Adverse Drug Reactions and Lipodystrophy in HIV; September 13-15 2000; Toronto, Canada. Antiretroviral Therapy 2000;5:38.
158. Dogan E.,Posaci C. Monitoring hormone replacement therapy by biochemical markers of bone metabolism in menopausal women. Postgrad Med J 2002; 78(926):727-31.
159. Gallagher JC, Rapuri PB, Haynatzki G, et al. Effect of discontinuation of estrogen, calcitriol, and the combination of both on bone density and bone markers. J Clin Endocrinol Metab 2002;87(11):4914-23.
160. Vrouenraets SM, Treskes M, Regez RM, et al. Hyperlactataemia in HIV-infected patients: the role of NRTI-treatment. Antivir Ther 2002;7(4):239-44.
161. Rietschel P, Hadigan C, Corcoran C, et al. Assessment of growth hormone dynamics in human immunodeficiency virus-related lipodystrophy. J Clin Endicrinol Metab 2001;86:504-10.
162. Bhasin S, Storer TW, Javanbakht M, et al. Testosterone replacement and resistance exercise in HIV infected men with weight loss and low testosterone levels. JAMA 2000; 283:763-70.
163. Hadigan C, Corcoran C, Piecuch S, et al. Hyeperandrogenemia in human immunodeficiency virus-infected women with the lipodystrophy syndrome. J Clin Endocrinol Metab 2000;85:3544-50.
164. Sheneberger R, Schneider S. An impact of protease inhibitors on thyroid stimulating hormone regulation? [Abstract WePeB4270] 13^th^ International AIDS Conference; July 9-14 2000; Durban, South Africa.
165. Estrada V, Serrano-Rios M, Martinez Larrad MT, et al. Leptin and adipose tissue maldistribution in HIV-infected male patients with predominant fat loss treated with antiretroviral therapy*.* J Acquir Immune Defic Syndr 2002;29(1):32-40..
166. Hadigan C, Corcoran C, Stanley T, et al. Fasting hyperinsulinemia in human immunodeficiency virus-infected men: relationship to body composition, gonadal function, and protease inhibitor use. J Clin Endocrinol Metab 2000;85:35-41.
167. Mynarcik DC., Combs T, McNurlan MA, et al. Adiponectin and leptin levels in HIV-infected subjects with insulin resistance and body fat redistribution. J Acquir Immune Defic Syndr 2002;31(5):514-20.
168. Noor MA, Lo JC, Mulligan K, et al. Metabolic effects of indinavir in healthy HIV-seronegative men. AIDS 2001;15:F11-8.
169. Thiebaut R, Daucourt V, Mercie P, et al.. Lipodystrophy, metabolic disorders, and human immunodeficiency virus infection: Aquitaine Cohort, France, 1999. Groupe d'Epidemiologie Clinique du Syndrome d'Immunodeficience Acquise en Aquitaine. Clin Infect Dis 2000;31(6):1482-7.
170. Haffner SM, Gonzalez C, Miettinen H, et al. A prospective analysis of the HOMA Model. Diabetes Care 1996;19:1138-41.
171. Jones SP, Doran DA, Leatt PB, et al. Short-term exercise training improves body composition and hyperlipidaemia in HIV-positive individuals with lipodystrophy. AIDS 2001;15(15):2049-51.
172. Chen D., Misra A., Garg A. Clinical review 153: Lipodystrophy in human immunodeficiency virus-infected patients. J Clin Endocrinol Metab 2002; 87(11):4845-56.
173. Roubenoff R., McDermott A., Weiss L., et al. Short-term progressive resistance training increases strength and lean body mass in adults infected with human immunodeficiency virus. AIDS 1999;13(2):231-9.
174. Poehlman ET, Denino WF, Beckett T, et al. Effects of endurance and resistance training on total daily energy expenditure in young women: a controlled randomized trial. J Clin Endocrinol Metab 2002;87(3):1004-9.
175. Simha V., Zerwekh J. E., Sakhaee K., et al. Effect of subcutaneous leptin replacement therapy on bone metabolism in patients with generalized lipodystrophy. J Clin Endocrinol Metab 2002;87(11):4942-5.
176. Arioglu E., Duncan-Morin J., Sebring N., et al. Efficacy and safety of troglitazone in the treatment of lipodystrophy syndromes. Ann Intern Med 2000;133(4):263-74.
177. Visnegarwala F, Maldonado M. Use of PPAR-y modulator Rosiglitazone in normoglycemic patients with HIV lipodystrophy syndrome. 84th Annual Meeting of The Endocrine Society, San Francisco, California, June 19-22, 2002. Abstract P2-355.
178. Sutinen J, Westerbacka J, Vehkavaara S, et al. Rosiglitazone in the Treatment of HAART Associated Lipodystrophy (HAL): A Randomized, Double-Blind, Placebo-Controlled Study. In 9th Conference on Retroviruses and Opportunistic Infections, 2002 Seattle, Washington.
179. Schambelan M, Mulligan K, Grunfeld C, et al. Recombinant human growth hormone in patients with HIV-associated wasting. A randomized, placebo-controlled trial. Serostim Study Group. Ann Intern Med 1996;125(11):873-82.
180. Gomez JM, Maravall FJ, Gomez N, et al. Pituitary-thyroid axis, thyroid volume and leptin in healthy adults. Horm Metab Res 2002;34(2):67-71.
181. Kotler DP, Wang J, Pierson RN. Body composition studies in patients with the acquired immunodeficiency syndrome. Am J Clin Nutr 1985;42(6):1255-65.
182. HIV Lipodystrophy Case Definition Study Group. An objective case definition of lipodystrophy in HIV-infected adults. Lancet 2003 (in press).
183. Carr A, Law MG, HIV Lipodystrophy Case Definition Study Group. An objective lipodystrophy severity grading scale derived from the lipodystrophy case definition score. AIDS (submitted).
184. At baseline and annually, serum will be stored for future measurements of the following parameters: Leptin, Free Fatty Acids, LDL subparticles, Total and Free testosterone, Cortisol, Thyroid Stimulating Hormone, Free T4, Insulin like Growth factor –1, Osteocalcin, Parathyroid hormone, 25-hydroxy vitamin cholecalciferol, Na, K, HCO_3_, Cl for anion gap, bone specific alkaline phosphatase, serum cross-linked N and C telopeptide, adiponectin.
185. Carr A, Workman C, Smith DE, et al. for the Mitochondrial Toxicity (MITOX) Study Group. Abacavir substitution for nucleoside analogs in patients with HIV lipoatrophy. JAMA 2002;288:207-215.
186. If a patient is known to have serologic evidence of hepatitis C by a positive antibody test or evidence of infection by detection of hepatitis C viral RNA, that information must be recorded. If hepatitis C serostatus is unavailable or negative and performed more than one year prior to screening, a hepatitis C antibody test must be performed.
187. If a patient is known to be positive for hepatitis B, either with a positive hepatitis B surface antibody [Anti-HBsAg], indicating immunity, or with two positive measurements of hepatitis B surface antigen [HBsAg] separated by 6 months, indicating chronic infection, at any time in the past prior to screening, that information must be recorded and no further testing is required. Otherwise, the hepatitis B surface antigen [HBsAg], hepatitis B surface antibody [anti-HBsAg], and hepatitis B IgG core antibody [IgG anti-HBc] tests must be performed. If the IgG core antibody test [IgG anti-HBc] is not available locally, a total core antibody test [IgG+IgM anti-HBc] should be performed and documented in its place
188. To be shipped to central, CDC-approved laboratory. Fasting status will be collected. For patients in the Body Composition substudy, this sample will be collected under fasting requirements, as part of the substudy data collection.
189. At selected sites; requires additional consent.
190. All patients at selected sites
191. Educational status
192. All patients at sites that are equipped with study-provided ECG machines.
193. All patients at sites that have access to study-certified BIA equipment
194. Total household income, type of health care insurance
195. Tests using DNA amplification (performed locally)
196. To be shipped to and stored at a central facility. Tests will be performed at the end of the study.
197. At least 12 hours fasting. Sample to be shipped to and analyzed at a central, CDC approved laboratory.
198. Requirements for drawing and processing the specimens for venous lactate are specified in the SMART Manual of Operations.
