# Supplementary material for: Inflammatory and Coagulation Biomarkers and Mortality in Patients with HIV Infection
Source: PLoS Med. 2008 Oct 21;5(10):e203. doi: 10.1371/journal.pmed.0050203 (PMC2570418; doi:10.1371/journal.pmed.0050203)
Supplement: Supplementary file 9 [file pmed.0050203.sg001.doc]

276 patients who died or had an AIDS, CVD, renal or liver event (182 DC and 94 VS) and 561 controls (291 DC and 270 VS) matched on age, country, gender and date of randomization

85 deaths

(55 DC and 30 VS)

74 deaths

(48 DC and 26 VS)

with follow-up specimen

312 controls

(157 DC and 155 VS)

with follow-up specimen

340 controls

(173 DC and 167 VS)

4 per death matched on date of randomization
