# Supplementary material for: Inflammatory and Coagulation Biomarkers and Mortality in Patients with HIV Infection
Source: PLoS Med. 2008 Oct 21;5(10):e203. doi: 10.1371/journal.pmed.0050203 (PMC2570418; doi:10.1371/journal.pmed.0050203)
Supplement: Supplementary file 10 [file pmed.0050203.st001.doc]

**Supplementary Table 1: Underlying Cause of Death for Participants in SMART^+^**

|  |  | **Treatment Group** | | | | | | |
| --- | --- | --- | --- | --- | --- | --- | --- | --- |
|  |  | **DC** |  | **VS** |  | **Total** | | |
| **Cause of Death** |  | **No.** |  | **No.** |  | **No.** |  | **Percent** |
| AIDS |  | 4 |  | 3 |  | 7 |  | 8.2 |
| Cancer, excluding AIDS-defining cancers |  | 11 |  | 5 |  | 16 |  | 18.8 |
| Cardiovascular disease |  | 7 |  | 4 |  | 11 |  | 12.9 |
| Infection |  | 3 |  | 1 |  | 4 |  | 4.7 |
| Hepatic disease |  | 1 |  | 2 |  | 3 |  | 3.5 |
| Renal disease |  | 3 |  | 0 |  | 3 |  | 3.5 |
| Chronic obstructive lung disease |  | 1 |  | 1 |  | 2 |  | 2.3 |
| Hematological disease |  | 2 |  | 0 |  | 2 |  | 2.3 |
| Digestive system disease |  | 1 |  | 1 |  | 2 |  | 2.3 |
| CNS disease |  | 1 |  | 0 |  | 1 |  | 1.2 |
| Respiratory disease |  | 0 |  | 1 |  | 1 |  | 1.2 |
| Substance abuse |  | 3 |  | 5 |  | 8 |  | 9.4 |
| Accident/violent/suicide |  | 3 |  | 4 |  | 7 |  | 8.2 |
| Cause unknown (unwitnessed) |  | 15 (8) |  | 3 (2) |  | 18 (10) |  | 21.2 (11.7) |
| Total |  | 55 |  | 30 |  | 85 |  | 100.0 |

^+^ Deaths occurring through January 11, 2006

DC = Drug Conservation

VS = Viral Suppression
