# Supplementary material for: Inflammatory and Coagulation Biomarkers and Mortality in Patients with HIV Infection
Source: PLoS Med. 2008 Oct 21;5(10):e203. doi: 10.1371/journal.pmed.0050203 (PMC2570418; doi:10.1371/journal.pmed.0050203)
Supplement: Supplementary file 11 [file pmed.0050203.st002.doc]

**Supplementary Table 2:	Odds Ratio Associated with a One IQR Higher Level of Biomarker at Study Entry: Three Models with Log_10_ Transformed Biomarker**

**^Model 1+	Model 2++	Model 3++^**

|  |  | **OR^+^ (95% CI)** |  | **P-value^+^** |  | **OR^++^ (95% CI)** |  | **P-value^++^** |  | **OR^+++^ (95% CI)** |  | **P-value^+++^** |
| --- | --- | --- | --- | --- | --- | --- | --- | --- | --- | --- | --- | --- |
| hsCRP (μg/mL) |  | 2.0 (1.3 – 3.1) |  | 0.002 |  | 2.1 (1.4 - 3.2) |  | 0.0007 |  | 2.3 (1.4 - 3.8) |  | 0.0008 |
| Amyloid A (mg/L) |  | 1.3 (0.9 - 1.8) |  | 0.17 |  | 1.2 (0.9 – 1.7) |  | 0.19 |  | 1.3 (0.8 – 2.0) |  | 0.32 |
| Amyloid P (μg/mL) |  | 0.7 (0.5 – 1.0) |  | 0.07 |  | 0.8 (0.6 – 1.0) |  | 0.06 |  | 1.1 (0.7 – 1.7) |  | 0.77 |
| IL-6 (pg/mL) |  | 3.3 (2.0 – 5.5) |  | <0.0001 |  | 3.6 (2.2 – 5.9) |  | <0.0001 |  | 3.9 (2.0 – 7.4) |  | <0.0001 |
| D-dimer (μg/mL) |  | 4.1 (2.2 – 7.5) |  | <0.0001 |  | 3.4 (2.0 – 5.8) |  | <0.0001 |  | 4.7 (2.3 – 9.6) |  | <0.0001 |
| F1.2 (pmol/L) |  | 1.0 (0.7 – 1.5) |  | 0.92 |  | 1.1 (0.8 – 1.6) |  | 0.51 |  | 1.1 (0.7 – 1.7) |  | 0.83 |

^+^Conditional logistic with reduced set of covariates (age, hepatitis co-infection, smoking, prior CVD, and CD4+ count).

^++^Unconditional logistic (matching ignored) with the following covariates measured at study entry: age, race, ART use and HIV-RNA, CD4+, smoking, BMI, prior CVD, diabetes, BP-lowering drugs, lipid-lowering drugs, total/HDL cholesterol, and hepatitis co-infection.

^+++^ Conditional logistic with all covariates but excluding outliers: hsCRP (1 control); amyloid A (6 deaths and 6 controls); amyloid P (10 deaths and 5 controls); IL-6 (8 deaths); D-dimer (2 deaths and 7 controls); F1.2 (5 deaths and 7 controls). These outliers can be identified on Figures S2-S7.
