# Supplementary material for: Inflammatory and Coagulation Biomarkers and Mortality in Patients with HIV Infection
Source: PLoS Med. 2008 Oct 21;5(10):e203. doi: 10.1371/journal.pmed.0050203 (PMC2570418; doi:10.1371/journal.pmed.0050203)
Supplement: Supplementary file 12 [file pmed.0050203.st003.doc]

**Supplementary Table 3: Risk of Death Associated with Biomarker Levels at Baseline – 4 Controls Per Death**

|  |  | < 25^th^  Percentile  (reference) |  | 25 – 49^th^ Percentile | | |  | 50 – 74^th^ Percentile | | |  | ≥ 75^th^ Percentile | | |  | OR associated with one IQR higher biomarker level after log_10_ transformation | | |
| --- | --- | --- | --- | --- | --- | --- | --- | --- | --- | --- | --- | --- | --- | --- | --- | --- | --- | --- |
|  |  |  |  | OR  (95% CI) |  | P-value |  | OR  (95% CI) |  | P-value |  | OR  (95% CI) |  | P-value |  | OR  (95% CI) |  | P-value |
|  |  |  |  |  |  |  |  |  |  |  |  |  |  |  |  |  |  |  |
| hsCRP (μg/mL) |  |  |  |  |  |  |  |  |  |  |  |  |  |  |  |  |  |  |
| no. cases/controls |  | 16/73 |  | 9/90 |  |  |  | 20/74 |  |  |  | 40/103 |  |  |  |  |  |  |
| univariate |  | 1.0 (ref.) |  | 0.4 (0.2-1.1) |  | 0.07 |  | 1.2 (0.6-2.4) |  | 0.70 |  | 1.7 (0.9-3.3) |  | 0.10 |  | 1.6 (1.1-2.2) |  | 0.006 |
| adjusted |  | 1.0 (ref.) |  | 0.5 (0.2-1.4) |  | 0.19 |  | 1.5 (0.6-3.5) |  | 0.40 |  | 3.0 (1.3-6.9) |  | 0.009 |  | 2.0 (1.4-3.0) |  | 0.0006 |
|  |  |  |  |  |  |  |  |  |  |  |  |  |  |  |  |  |  |  |
| Amyloid A (mg/L) |  |  |  |  |  |  |  |  |  |  |  |  |  |  |  |  |  |  |
| no. cases/controls |  | 11/94 |  | 17/71 |  |  |  | 28/84 |  |  |  | 29/91 |  |  |  |  |  |  |
| univariate |  | 1.0 (ref.) |  | 2.1 (0.9-4.8) |  | 0.08 |  | 2.8 (1.3-6.0) |  | 0.007 |  | 2.6 (1.3-5.6) |  | 0.01 |  | 1.3 (1.0-1.6) |  | 0.03 |
| adjusted |  | 1.0 (ref.) |  | 2.0 (0.8-5.2) |  | 0.15 |  | 2.5 (1.0-6.1) |  | 0.05 |  | 3.0 (1.3-7.0) |  | 0.01 |  | 1.3 (1.0-1.7) |  | 0.04 |
|  |  |  |  |  |  |  |  |  |  |  |  |  |  |  |  |  |  |  |
| Amyloid P (μg/mL) |  |  |  |  |  |  |  |  |  |  |  |  |  |  |  |  |  |  |
| no. cases/controls |  | 25/80 |  | 20/62 |  |  |  | 16/94 |  |  |  | 24/104 |  |  |  |  |  |  |
| univariate |  | 1.0 (ref.) |  | 1.1 (0.5-2.1) |  | 0.85 |  | 0.6 (0.3-1.1) |  | 0.10 |  | 0.7 (0.4-1.4) |  | 0.35 |  | 0.7 (0.6-0.9) |  | 0.003 |
| adjusted |  | 1.0 (ref.) |  | 1.5 (0.6-3.3) |  | 0.38 |  | 0.9 (0.4-1.9) |  | 0.75 |  | 1.1 (0.5-2.3) |  | 0.84 |  | 0.8 (0.6-1.0) |  | 0.06 |
|  |  |  |  |  |  |  |  |  |  |  |  |  |  |  |  |  |  |  |
| IL-6 (pg/mL) |  |  |  |  |  |  |  |  |  |  |  |  |  |  |  |  |  |  |
| no. cases/controls |  | 8/103 |  | 10/79 |  |  |  | 26/78 |  |  |  | 40/80 |  |  |  |  |  |  |
| univariate |  | 1.0 (ref.) |  | 1.6 (0.6-4.2) |  | 0.35 |  | 4.2 (1.8-9.8) |  | 0.001 |  | 6.1 (2.7-13.6) |  | <0.0001 |  | 2.4 (1.7-3.2) |  | <0.0001 |
| adjusted |  | 1.0 (ref.) |  | 1.7 (0.6-5.0) |  | 0.33 |  | 4.8 (1.9-12.4) |  | 0.001 |  | 7.3 (2.8-19.1) |  | <0.0001 |  | 2.6 (1.8-3.8) |  | <0.0001 |
|  |  |  |  |  |  |  |  |  |  |  |  |  |  |  |  |  |  |  |
| D-dimer (μg/mL) |  |  |  |  |  |  |  |  |  |  |  |  |  |  |  |  |  |  |
| no. cases/controls |  | 8/93 |  | 22/93 |  |  |  | 18/94 |  |  |  | 37/60 |  |  |  |  |  |  |
| univariate |  | 1.0 (ref.) |  | 2.6 (1.1-6.1) |  | 0.03 |  | 2.2 (0.9-5.4) |  | 0.08 |  | 6.6 (2.9-14.9) |  | <0.0001 |  | 2.6 (1.8-3.8) |  | <0.0001 |
| adjusted |  | 1.0 (ref.) |  | 3.9 (1.5-10.6) |  | 0.007 |  | 2.4 (0.8-6.9) |  | 0.11 |  | 10.1 (3.5-29.2) |  | <0.0001 |  | 2.9 (1.8-4.7) |  | <0.0001 |
|  |  |  |  |  |  |  |  |  |  |  |  |  |  |  |  |  |  |  |
| F1.2 (pmol/L) |  |  |  |  |  |  |  |  |  |  |  |  |  |  |  |  |  |  |
| no. cases/controls |  | 17/61 |  | 21/95 |  |  |  | 15/88 |  |  |  | 31/96 |  |  |  |  |  |  |
| univariate |  | 1.0 (ref.) |  | 0.8 (0.4-1.6) |  | 0.53 |  | 0.6 (0.3-1.3) |  | 0.22 |  | 1.1 (0.6-2.2) |  | 0.73 |  | 1.1 (0.8-1.5) |  | 0.47 |
| adjusted |  | 1.0 (ref.) |  | 1.1 (0.5-2.6) |  | 0.74 |  | 0.8 (0.3-1.9) |  | 0.62 |  | 1.8 (0.8-4.1) |  | 0.15 |  | 1.2 (0.9-1.7) |  | 0.23 |
