# Supplementary material for: Inflammatory and Coagulation Biomarkers and Mortality in Patients with HIV Infection
Source: PLoS Med. 2008 Oct 21;5(10):e203. doi: 10.1371/journal.pmed.0050203 (PMC2570418; doi:10.1371/journal.pmed.0050203)
Supplement: Supplementary file 13 [file pmed.0050203.st004.doc]

**Supplementary Table 4:	Study Entry and Latest Levels of Six Biomarkers for Deaths and Matched Controls (Four Matched Controls for each Death)**

|  |  | **Deaths** |  | **Controls** |  | **Difference**  **(Case-Control)**  **after log_10_**  **transformation**  **(SE)** |  |  |
| --- | --- | --- | --- | --- | --- | --- | --- | --- |
|  |  | **Median (25^th^, 75^th^ %)** |  | **Median (25^th^, 75^th^ %)** |  |  |  | **P-value^+++^** |
| Study Entry^+^ |  |  |  |  |  |  |  |  |
| hsCRP (μg/mL) |  | 4.26 (2.12, 7.49) |  | 2.51 (1.04, 5.19) |  | 0.18 (0.06) |  | 0.006 |
| Amyloid A (mg/L) |  | 4.75 (2.80, 9.06) |  | 3.57 (1.87, 6.60) |  | 0.13 (0.06) |  | 0.03 |
| Amyloid P (μg/mL) |  | 58.8 (43.1, 82.3) |  | 67.2 (50.1, 87.0) |  | -0.08 (0.03) |  | 0.003 |
| IL-6 (pg/mL) |  | 3.80 (2.72, 7.20) |  | 2.26 (1.47, 3.80) |  | 0.26 (0.04) |  | <0.0001 |
| D-dimer (μg/mL) |  | 0.49 (0.27, 1.16) |  | 0.29 (0.17, 0.52) |  | 0.29 (0.05) |  | <0.0001 |
| F1.2 (pmol/L) |  | 344.0 (245.8, 565.8) |  | 326.6 (245.7, 504.4) |  | 0.03 (0.03) |  | 0.47 |
|  |  |  |  |  |  |  |  |  |
| Latest Level^++^ |  |  |  |  |  |  |  |  |
| hsCRP (μg/mL) |  | 5.26 (2.19, 19.3) |  | 2.29 (0.96, 4.91) |  | 0.45 (0.07) |  | <0.0001 |
| Amyloid A (mg/L) |  | 6.88 (2.40, 16.7) |  | 3.59 (2.00, 6.73) |  | 0.30 (0.07) |  | 0.0001 |
| Amyloid P (μg/mL) |  | 57.7 (34.9, 78.5) |  | 67.3 (47.7, 85.6) |  | -0.08 (0.03) |  | 0.002 |
| IL-6 (pg/mL) |  | 7.84 (3.08, 15.5) |  | 2.61 (1.66, 4.13) |  | 0.46 (0.05) |  | <0.0001 |
| D-dimer (μg/mL) |  | 0.70 (0.34, 1.64) |  | 0.35 (0.20, 0.63) |  | 0.36 (0.05) |  | <0.0001 |
| F1.2 (pmol/L) |  | 339.5 (260.6, 463.7) |  | 320.6 (223.3, 474.3) |  | 0.00 (0.03) |  | 0.89 |

^+^85 deaths and 340 controls.

++74 deaths and 312 controls.

+++P-value obtained from univariate conditional logistic model with log_10_ transformed biomarker level.
