# Supplementary material for: Inflammatory and Coagulation Biomarkers and Mortality in Patients with HIV Infection
Source: PLoS Med. 2008 Oct 21;5(10):e203. doi: 10.1371/journal.pmed.0050203 (PMC2570418; doi:10.1371/journal.pmed.0050203)
Supplement: Supplementary file 14 [file pmed.0050203.st005.doc]

**Supplementary Table 5: Estimates of the Odds Ratio for DC Versus VS Participants for All-Cause Mortality After Adjustment for Study Entry and Latest Levels of IL-6, D-dimer, CD4+ Cell count and HIV RNA level**

|  |  | **74 deaths and 141 controls** | | | | |  | **74 deaths and 296 controls** | | | | |
| --- | --- | --- | --- | --- | --- | --- | --- | --- | --- | --- | --- | --- |
|  |  | **Odds**  **Ratio** |  | **95% CI** |  | **P-value** |  | **Odds**  **Ratio** |  | **95% CI** |  | **P-value** |
| Unadjusted |  | 1.2^+^ |  | 0.7 – 2.1 |  | 0.59 |  | 1.8 |  | 1.1 – 3.1 |  | 0.02 |
|  |  |  |  |  |  |  |  |  |  |  |  |  |
| Adjusted for: |  |  |  |  |  |  |  |  |  |  |  |  |
| IL-6 (log_10_ pg/mL) |  | 1.3 |  | 0.6 – 2.8 |  | 0.53 |  | 1.6 |  | 0.9 – 3.0 |  | 0.11 |
| D-dimer (log_10_ μg/mL) |  | 0.9 |  | 0.5 – 1.9 |  | 0.85 |  | 1.5 |  | 0.9 – 2.7 |  | 0.15 |
| IL-6 and D-dimer |  | 1.4 |  | 0.6-3.3 |  | 0.43 |  | 1.6 |  | 0.9-3.1 |  | 0.13 |
| IL-6, D-dimer and CD4+ count (log_10_ cells/mm^3^) |  | 1.2 |  | 0.4-3.5 |  | 0.68 |  | 1.4 |  | 0.7-2.9 |  | 0.36 |
| IL-6, D-dimer, CD4+ cell count, and HIV RNA level (log_10_ copies/mL) |  | 1.3 |  | 0.4-4.1 |  | 0.66 |  | 1.6 |  | 0.7-3.4 |  | 0.23 |

^+^ By chance, among the 170 matched controls chosen for the 85 deaths, 99 were in the DC group and 71 were in the VS group. This resulted in an unadjusted OR using the 85 deaths of 1.3 (95% CI: 0.8 to 2.2), an estimate considerably smaller than that reported in reference 1: 1.8 (95% CI: 1.2 to 2.9). When the analysis was restricted to those with follow-up data, 7 DC and 4 VS participants who died and 14 DC controls and 15 VS controls were excluded and the resulting OR is shown in the table.
